# Checklist of hover flies (Diptera, Syrphidae) of the Republic of Georgia

**DOI:** 10.3897/zookeys.916.47824

**Published:** 2020-03-02

**Authors:** Ximo Mengual, Sander Bot, Tinatin Chkhartishvili, André Reimann, Jana Thormann, Laura von der Mark

**Affiliations:** 1 Zoologisches Forschungsmuseum Alexander Koenig, Leibniz-Institut für Biodiversität der Tiere, Adenauerallee 160, D-53113 Bonn, Germany Leibniz-Institut für Biodiversität der Tiere Bonn Germany; 2 Kerklaan 30E, 9751 NN Haren, the Netherlands Unaffiliated Haren Netherlands; 3 Insititute of Zoology, Ilia State University, Chavchavadze Avenue 32, 0179, Tbilisi, Georgia Ilia State University Tbilisi Georgia; 4 Senckenberg Naturhistorische Sammlungen Dresden, Museum für Tierkunde, Königsbrücker Landstraße 159, D-01109, Dresden, Germany Senckenberg Naturhistorische Sammlungen Dresden Dresden Germany

**Keywords:** DNA barcoding, faunistics, first record, flower flies, hover flies, new record, species list

## Abstract

A checklist of the Syrphidae species of the Republic of Georgia is presented. New hover fly (Diptera: Syrphidae) records from Georgia are provided as a result of field work conducted in 2018. At the same time, published syrphid records for the country are here reviewed and updated. A total of 357 species of hoverflies are now documented from Georgia, 40 of which are reported for the first time. Moreover, DNA barcodes were sequenced for 238 specimens, representing 74 species from this country.

## Introduction

With an almost worldwide distribution, absent from Antarctica and remote oceanic islands, Syrphidae is a very species-rich family of Diptera with more than 6,000 described species (Brown 2009; Thompson 2019). Commonly called flower flies or hover flies, adults are associated with flowers that are used as mating sites and energy food sources (pollen and nectar). They are considered essential pollinators of wild flowering plants and crops (Pérez-Bañón et al. 2003a; Ssymank and Kearns 2009; Inouye et al. 2015) and have been used as bioindicators in order to evaluate biodiversity loss and the efficiency of restoration and conservation policies (Sommaggio 1999; Tscharntke et al. 2005; Biesmeijer et al. 2006; Ricarte et al. 2011; Sommaggio and Burgio 2014). Syrphid immatures have a large array of natural histories and are variable in structure and feeding modes (Rotheray 1993, Rotheray and Gilbert 1999, 2011). Some of these larvae play an important role as biological control agents of pests (Schmidt et al. 2004; Bergh and Short 2008; Bugg et al. 2008; Nelson et al. 2012; Eckberg et al. 2015) or as decomposers of organic matter (Lardé 1989; Martínez-Falcón et al. 2012), but some phytophagous larvae may be considered plant pests under certain circumstances (Edwards and Bevan 1951; Stuckenberg 1956; Tompsett 2002).

**Figure 1.**
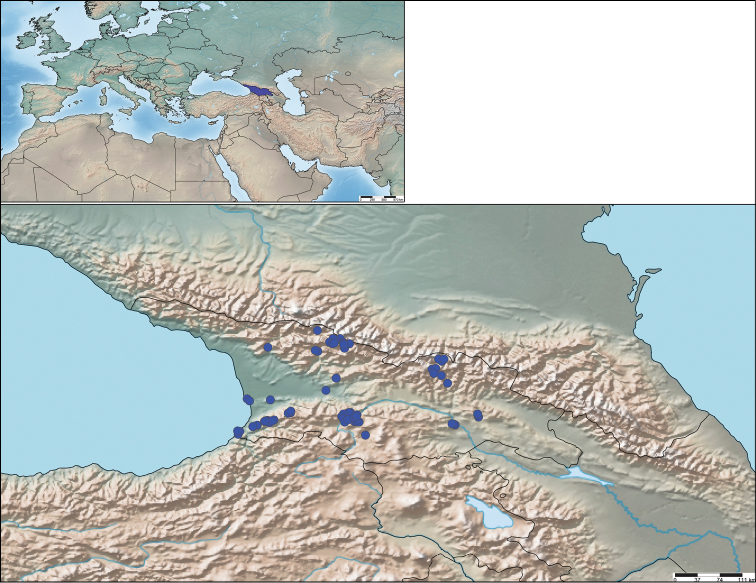
Sampling localities in Georgia during the field work in 2018.

The Caucasus Region is situated between the Black Sea and the Caspian Sea (Fig. 1) and is one of the global ‘biodiversity hotspots’ (Myers et al. 2000; Mittermeier et al. 2004; Zazanashvili et al. 2004). The region comprises the Republics of Armenia, Azerbaijan and Georgia (sometimes all together called Transcaucasia), parts of northwestern Turkey, northern Iran, and Russian republics and krais between the Sea of Azov and Black Sea on the west and the Caspian Sea on the east (area known as Ciscaucasia or Northern Caucasus). Georgia lies in the central part of the Caucasus Region and has two major mountainous ranges, i.e., the Greater Caucasus and the Lesser Caucasus.

Regarding species diversity, there is a geographic gap of knowledge in the Caucasus Region, especially on the dipteran fauna (Insecta: Diptera) (Wetzel et al. 2008; Oboňa et al. 2019). The hover fly fauna of Georgia has never been studied in detail. Previous works on the Syrphidae fauna of the Caucasus Region are predominantly done by Soviet authors, published mainly in Russian-language magazines, focusing in the fauna of the Northern Caucasus, although some include the fauna of Armenia and/or Azerbaijan (Portschinsky 1877; Radde 1899; Zaitzev 1912; Paramonov 1926a, 1926b, 1927a, 1927b; Stackelberg 1926, 1960, 1968; Zimina 1960, 1976; Skufjin 1967, 1976; Stackelberg and Richter 1968; Tóth and Günther 1992; Barkalov 1993; Kustov 2006, among others). More recently, Hauser (1998) presented new records for Azerbaijan with the description of two new species. Regarding Georgian syrphid fauna, Levitin (1962) and Tóth (1986) were the only authors who explicitly stated that their studies were entirely conducted in Georgia, and Gudjabidze (2002) is the only work known to the authors with a clear focus to document the syrphid fauna of Georgia. In addition, there are two general bibliographic references for the Palaearctic with notes on the Caucasus Region. The first is Peck (1988), who in her catalogue of Palaearctic Diptera barely mentioned Georgia explicitly, but used very often the term Transcaucasia (TC). Since then, no other work treated the entire Palaearctic syrphid fauna. The second reference is Speight (2018a), who focused on European species of hover flies. Speight (2018a) is a compilation of published works with some personal comments, but includes the distribution of the species with mentions to the Caucasus Region.

Since 2012, the Zoologisches Forschungsmuseum Alexander Koenig (ZFMK) has coordinated and led the project German Barcode of Life (GBOL; https://www.bolgermany.de/), an initiative to create a DNA barcode library (Hebert et al. 2003a, 2003b) of the German animals, plants and fungi (Geiger et al. 2016). In 2017, the German Federal Ministry of Education and Research (BMBF) granted a proposal to set up a Georgian-German Biodiversity Center (GGBC) as a multinational approach to explore the biodiversity of the Caucasus area (grant number 01DK17048; project’s website: https://ggbc.eu/). The experience of GBOL and its infrastructure is supposed to serve as a model for the development of a comparable structure in Georgia, together with other knowledge transfer and exchange of students and researchers between the ISU (Ilia State University, Tbilisi, Georgia) and the ZFMK (Thormann et al. 2019). A continuation of the GGBC is currently planned as a Georgian-Armenian-German initiative, the Caucasus Barcode of Life Platform (CaBOL) (see Thormann et al. 2019 for more details). In the present work, we report the results of a collection expedition between June and July of 2018, as part of the collaboration effort between the ISU and the ZFMK. Within the GGBC framework, we here provide the first DNA barcodes for the syrphid fauna of Georgia, a stepping-stone for ongoing (GGBC) and planned (CaBOL) projects.

## Materials and methods

### Literature records

Authors used Peck (1988) as the primary source. In this publication, we registered all the species listed in the Transcaucasia (TC), i.e., south of the main ridge of the Caucasus, including Georgia, Armenia and Azerbaijan. Unless the country was explicitly indicated, we have listed to occur in Georgia all the TC species from Peck’s (1988) catalogue, and added a note when the species was explicitly listed from Georgia. Based on that keystone publication, we critically reviewed published literature up to date in order to find Georgian records. We assumed that Peck (1988) summarized other important works on the syrphid fauna of the Caucasus such as Stackelberg and Richter (1968) or Levitin (1962), who provided the first Syrphidae records from the Borjomi area, in Lesser Caucasus. Nevertheless, we also studied Levitin (1962) and Stackelberg and Richter (1968) in case some more precise locality details were mentioned in the original works.

In addition, we consulted the Georgian Biodiversity Database (http://biodiversity-georgia.net/index.php), which is a digital compilation of field observations and a summary of the work by Gudjabidze (2002), and two more general publications that somehow were updates of Peck (1988): Barkalov and Mutin (2018) and Speight (2018a). From Speight (2018a), we incorporated species listed from Georgia or the Caucasus; while from Barkalov and Mutin (2018), we included all the species listed as Transcaucasia (TC), although no Georgian records were explicitly given. If a species was listed as TC but no Georgian record was explicitly given, we indicate our records as the first ones for Georgia. Other more specific articles devoted to single taxa were all checked for Georgian records.

We did not study type material for species with uncertain taxonomic status. A revision of the taxonomic status of such species is beyond the scope of the present work. When appropriate, we have indicated such uncertainty under the species remarks. In the same line, we did not study the material reported from Georgia or Transcaucasia from other authors or published works. Nevertheless, we have indicated some remarks about the identification of previous published material.

For the current distribution of the listed species we used Speight (2018a) as the most up-to-date reference, although other published works were consulted for specific taxa in order to obtain a more accurate distribution. We used three different categories in the current distribution with comments: 1) realms such as Palaearctic when the species has a very broad distribution; 2) regions such as Europe or Transcaucasia; and 3) countries, like Georgia, when the species is only known from those countries. For a more detailed geographic distribution, we refer to Speight (2018a).

We need to point out that Gudjabidze (2002) has several systematic and nomenclatural errors. For the sake of traceability, we indicated such nomenclatural errors in the text with a [sic] (*sic erat scriptum* = thus was it written, intentionally so written).

### New records

Field expedition took place between 15 June and 27 July 2018. Several Georgian provinces were visited (see Table 1) and all the specimens were collected using a hand-net except where indicated. Specimens collected by Sander Bot are deposited in the Sander Bot’s Personal Collection (**SBPC**; Haren, the Netherlands); specimens collected by André Reimann and Björn Rulik are deposited in the Senckenberg Museum für Tierkunde (**MTD**; Dresden, Germany) and in the Zoologisches Forschungsmuseum Alexander Koenig (**ZFMK**; Bonn, Germany); and specimens collected by Birthe Thormann, Jana Thormann, Benedikt Wipfler, David Tarkhnishvili, Jonas Astrin, Hans-Joachim Krammer, Marianne Espeland and Ximo Mengual are deposited in the Zoologisches Forschungsmuseum Alexander Koenig (**ZFMK**; Bonn, Germany). The flower flies of three malaise trap samples taken between 29 June and 14 July 2018 in the Kintrishi region as part of the GGBC project (Thormann et al. 2019) were sorted and studied by André Reimann and are also included in the present work. These specimens are deposited in the Zoologisches Forschungsmuseum Alexander Koenig (ZFMK; Bonn, Germany) and some duplicates are deposited in the Senckenberg Museum für Tierkunde (MTD; Dresden, Germany). In addition, material collected in Georgia by Jens-Hermann Stuke in 2001 and deposited at the ZFMK was studied by the first author and included as well.

A list of all the sampling localities with detailed information is given in Table 1 and illustrated in Fig. 1. Geographical coordinates were taken in the field and later corrected using Google Earth ®. SimpleMappr (Shorthouse 2010) was used to create Fig. 1. For the new records, the locality number is given following Table 1 and we indicate the number of specimens and sex and the unique identifier or number at the end. Specimens with unique identifiers starting with ZFMK-DIP or ZFMK-TIS are deposited in the ZFMK collections and are unique for each specimen, while identifiers starting with MTD denote single specimens or lots (group of specimens from the same collecting event) and are deposited in the MTD collections.

Specimens marked with an asterisk (*) are field observations only, so these fly/flies have not been collected. No additional photographic material exists for these field observations.

**Table 1.** Sampling localities in Georgia from the 2018 field work.

Locality number	Locality	Coordinates	Altitude [m]
L1	Imereti Region, near Borjomi-Kharagauli National Park, Likani	41°49.912'N, 43°20.725'E	850
L2	Imereti Region, Borjomi-Kharagauli National Park, Route 6	41°49.462'N, 43°18.01'E	1330
L3	Imereti Region, Borjomi-Kharagauli National Park, crossing Route 6 and 1	41°49.87'N, 43°16.12'E	1780
L4	Imereti Region, Borjomi-Kharagauli National Park, near Mt. Lomismta	41°52.002'N, 43°15.034'E	2000
L5	Imereti Region, Borjomi-Kharagauli National Park, Route 9	41°51.955'N, 43°13.265'E	1900
L6	Imereti Region, Borjomi-Kharagauli National Park, near Megruki river	41°50.76'N, 43°08.7'E	1800
L7	Imereti Region, Borjomi-Kharagauli National Park, Amarati tourist shelter	41°48.623'N, 43°07.127'E	2050
L8	Imereti Region, Borjomi-Kharagauli National Park, Route 3	41°46.628'N, 43°08.642'E	1720
L9	Samegrelo-Zemo Svaneti, along Enguri Reservoir	42°50.046'N, 42°01.343'E	600
L10	Samegrelo-Zemo Svaneti, slopes northeast of Mestia	43°05.04'N, 42°45.52'E	1800
L11	Samegrelo-Zemo Svaneti, Ushguli	42°55.022'N, 43°01.065'E	2100
L12	Samegrelo-Zemo Svaneti, between Ushguli and Shkhara glacier	42°57.034'N, 43°04.492'E	2275
L13	Samegrelo-Zemo Svaneti, Shkhara glacier	42°57.84'N, 43°05.605'E	2500
L14	Samegrelo-Zemo Svaneti, north of Ushguli	42°56.88'N, 43°01.26'E	2800
L15	Samegrelo-Zemo Svaneti, north of Ushguli	42°57.78'N, 42°59.4'E	2300
L16	Samegrelo-Zemo Svaneti, south of Ushguli	42°54.724'N, 42°56.29'E	2430
L17	Samegrelo-Zemo Svaneti, top of ridge, south of Ushguli	42°53.287'N, 42°58.736'E	2828
L18	Samegrelo-Zemo Svaneti, slopes south of Ushguli	42°53.82'N, 43°00.48'E	2630
L19	Racha-Lechkhumi and Kvemo Svaneti, along road north of Tsana	42°54.439'N, 43°08.52'E	1900
L20	Racha-Lechkhumi and Kvemo Svaneti, east of Zeskho	42°53.291'N, 43°13.978'E	1900
L21	Racha-Lechkhumi and Kvemo Svaneti, where Zeskho and Tskhenistskali rivers meet	42°49.273'N, 43°09.638'E	1400
L22	Racha-Lechkhumi and Kvemo Svaneti, just east of Lentekhi	42°47.218'N, 42°44.545'E	800
L23	Tbilisi, Tbilisi city, National Botanical Garden	41°41.04'N, 44°48.18'E	500
L24	Adjara Region, Mtirala National Park, Chesnut trail	41°40.725'N, 41°51.878'E	290
L25	Adjara Region, Kintrishi Nature Reserve	41°44.069'N, 41°59.332'E	460
L26	Adjara Region, Kintrishi Nature Reserve	41°44.282'N, 42°00.455'E	595
L27	Guria Region, north from Atsana, along the road	42°03.355'N, 42°03.563'E	275
L28	Guria Region, road to Barkhmaro, creek	41°51.655'N, 42°21.431'E	1935
L29	Guria Region, Barkhmaro, forest	41°51.46'N, 42°19.442'E	2050
L30	Guria Region, road to Barkhmaro, meadow	41°53.179'N, 42°21.685'E	1645
L31	Adjara Region, Kintrishi Nature Reserve, Khino	41°44.069'N, 41°59.498'E	980
L32	Samtskhe-Javakheti, road from Sakire to Tsikhisjvari	41°43.957'N, 43°18.49'E	1600
L33	Samtskhe-Javakheti, road from Sakire to Tsikhisjvari	41°43.82'N, 43°20.087'E	1910
L34	Samtskhe-Javakheti, road from Sakire to Tsikhisjvari	41°43.625'N, 43°22.637'E	2185
L35	Racha-Lechkhumi and Kvemo Svaneti, Tsana	42°53.332'N, 43°08.58'E	1760–1775
L36	Racha-Lechkhumi and Kvemo Svaneti, road to Tsana	42°52.436'N, 43°09'E	1600
L37	Racha-Lechkhumi and Kvemo Svaneti, Lentekhi towards antennae, meadow	42°46.646'N, 42°45.011'E	1370–1405
L38	Racha-Lechkhumi and Kvemo Svaneti, near Lentekhi, antennae, meadow	42°46.468'N, 42°45.814'E	1710
L39	Racha-Lechkhumi and Kvemo Svaneti, Lentekhi	42°47.42'N, 42°43.556'E	760
L40	Adjara Region, Kintrishi Nature Reserve, Didvake, forest	41°44.779'N, 42°01.001'E	1102
L41	Adjara Region, Kintrishi Nature Reserve	41°44.095'N, 41°58.262'E	445
L42	Adjara Region, Mtirala National Park, Mount Mtirala	41°39.484'N, 41°47.9'E	1320
L43	Racha-Lechkhumi and Kvemo Svaneti, Tskhmori, Chalistskali waterfall	42°31.875'N, 43°28.255'E	1225
L44	Imereti Region, NE of Tkibuli, Nakerala Pass	42°22.622'N, 43°02.209'E	1236
L45	Samtskhe-Javakheti, Akhaltsikhe, 3 km NE of Tsinubani	41°43.51'N, 43°09.81'E	904
L46	Samtskhe-Javakheti, Borjomi, mountain lake and surroundings, 9.5 km SSW of Borjomi	41°45.42'N, 43°20.71'E	1770
L47	Samtskhe-Javakheti, Borjomi, river valley 7 km SSW Chitakhevi	41°45.53'N, 43°12.59'E	910
L48	Samtskhe-Javakheti, Borjomi, river valley 8 km SW Borjomi	41°47.45'N, 43°18.22'E	840
L49	Mtskheta-Mtianeti, Dusheti, river valley 18 km SSE Kobi	42°24.94'N, 44°35.95'E	1245
L50	Mtskheta-Mtianeti, Dusheti, river valley 30 km SSE Kobi	42°18.13'N, 44°41.41'E	995
L51	Samtskhe-Javakheti, river valley 5 km SW Borjomi	41°48.61'N, 43°20.12'E	840
L52	Mtskheta-Mtianeti, Stepantsminda, shallow moor at highway	42°30.59'N, 44°27.65'E	2372
L53	Mtskheta-Mtianeti, Stepantsminda, river valley SW Stepantsminda	42°37.61'N, 44°36.38'E	1772
L54	Mtskheta-Mtianeti, Stepantsminda, glacial river 6 km west of Stepantsminda	42°39.61'N, 44°33.39'E	2800–3035
L55	Mtskheta-Mtianeti, Stepantsminda, gravel surface 3 km SSE of Kobi	42°31.21'N, 44°30.92'E	2885
L56	Mtskheta-Mtianeti, Stepantsminda, highway near Kumlistsikh	42°26.82'N, 44°29.18'E	1835
L57	Mtskheta-Mtianeti, Stepantsminda, slopes west of Stepantsminda	42°39.55'N, 44°38.20'E	2500
L58	Adjara Region, Khelvachauri, hill 2 km E of Gonio	41°32.66'N, 41°34.91'E	420
L59	Adjara Region, Khelvachauri, river delta 7 km SSW of Batumi	41°35.23'N, 41°36.28'E	7
L60	Adjara Region, Khelvachauri, river delta 8 km SW of Batumi	41°35.66'N, 41°34.37'E	2
L61	Tbilisi, Tbilisi city, park near TV tower	41°41.62'N, 44°47.20'E	715
L62	Tbilisi, Tbilisi city, Turtle Lake	41°42.11'N, 44°45.31'E	692
L63	Kakheti, Sagarejo, 28 km ENE Tbilisi, near Ujarma	41°48'N, 45°09'E	890
L64	Kakheti, Sagarejo, 30 km ENE Tbilisi, near Paldo	41°49.79'N, 45°08.44'E	845
L65	Kakheti, Sagarejo, 32 km ENE Tbilisi, near Iori Reservoir	41°50.73'N, 45°08.20'E	862
L66	Samegrelo-Zemo Svaneti, shore 8 km SSW of Poti	42°04.29'N, 41°42.85'E	0
L67	Guria, wetland 4 km W Ghrmaghele (E of Grigoleti)	42°02.40'N, 41°45.02'E	0
L68	Imereti Region, Zestaponi, river valley 12 km NW Zestaponi	42°11.77'N, 42°53.04'E	137
L69	Adjara Region, Kintrishi Nature Reserve, road side between Didvake and Khino	41°44.734'N, 42°0.844'E –41°43.039'N, 42°02.749'E	790 – 1100
L70	Adjara Region, Kintrishi Nature Reserve, Krummholtz forest, Malaise trap 13	41°45.31'N, 42°06.75'E	2268
L71	Adjara Region, Kintrishi Nature Reserve, above the waterfall, Malaise trap 6	41°44.6'N, 42°05.045'E	1235
L72	Adjara Region, Kintrishi Nature Reserve, woods at Khino monastry, Malaise trap 8	41°44.267'N, 41°58.715'E	403

### Adult identification

General works with identification keys were used for generic level, i.e., Thompson and Rotheray (1998), Van Veen (2010), Speight (2018b). More specific works were used to species identifications, for instance Speight and Sarthou (2017) for several genera, Barkalov (1993) for *Cheilosia* Meigen, 1822; Stuke and Nielsen (2002) and Barkalov and Nielsen (2007) for *Platycheirus* Le Peletier and Audinet-Serville, 1828; Doczkal (2002) for *Leucozona* Schiner, 1860; Van Steenis and Lucas (2011) for *Pipizella* Rondani, 1856; Vujić et al. (2013) for *Pipiza* Fallén, 1810; Van Steenis et al. (2016) for *Ceriana* Rafinesque, 1815; Hayat and Claußen (1997) and Sorokina (2009) for *Paragus* Latreille, 1804; Lyneborg and Barkemeyer (2005) for *Syritta* Le Peletier and Audinet-Serville, 1828; Violovitsh (1974), Vujić et al. (2017) and Kazerani et al. (2017) for *Chrysotoxum* Meigen, 1803; Reemer et al. (2005) for *Myolepta* Newman, 1838; Stackelberg (1958) and Van Steenis (2000) for *Spilomyia* Meigen, 1803; Krivosheina (2002, 2004, 2005) for *Temnostoma* Le Peletier and Audinet-Serville, 1828; and Hippa (1968) for *Xylota* Meigen, 1822.

The subgenus for each species, when this applies, has been indicated with the exception of the genus *Eristalinus*, as current subdivision of this genus based on morphological characteristics of the eyes in males (Thompson and Rotheray 1998) is not supported by molecular analyses (Pérez-Bañón et al. 2003b; Sonet et al. 2019).

For the author of the names published in Meigen (1822), the original work by Meigen was used. For the new species published in Meigen (1822), authorship was given to Hoffmannsegg when the abbreviation *Hgg*. appeared after the name; Megerle when abbreviation *Meg*. appeared after the name; Wiedemann when abbreviation *Wied*. appeared after the name; and to Meigen when no abbreviation was written after the name of the new species.

### Molecular methods

DNA barcodes (Hebert et al. 2003a, 2003b) were generated by following the standard protocols of the GBOL (German Barcode of Life) project (Geiger et al. 2016; http://www.bolgermany.de). Total genomic DNA was isolated from one or two legs using the DNeasy Blood and Tissue Kit and the BioSprint96 magnetic bead extractor by Qiagen (Hilden, Germany). Remnants of specimens were preserved and labelled as DNA voucher specimens for the purpose of morphological studies and deposited at the ZFMK. Polymerase chain reaction (PCR) for the mitochondrial cytochrome c oxidase subunit 1 (COI) gene was carried out in total reaction mixes of 20 μl, including 2 μl of undiluted DNA template, 0.8 μl of each primer (10 pmol/μl), and standard amounts of the reagents provided with the 'Multiplex PCR' kit from Qiagen (Hilden, Germany). LCO1490-JJ [5'-CHACWAAYCATAAAGATATYGG-3'] and HCO2198-JJ [5'-AWACTTCVGGRTGVCC AAARAATCA-3'] (Astrin and Stüben 2008) were used as standard primers.

Thermal cycling was performed on Applied Biosystems 2720 Thermal Cyclers (Life Technologies, Carlsbad, CA, USA), using a PCR program with two cycle sets, as a combination of a 'touchdown' and a 'step-up' routine as follows: hot start Taq activation: 15 min at 95 °C ; first cycle set (15 repeats): 35 s denaturation at 94°C, 90 s annealing at 55°C (−1°C per cycle) and 90 s extension at 72°C; second cycle set (25 repeats): 35 s denaturation at 94°C, 90 s annealing at 40°C, and 90 s extension at 72°C; final elongation 10 min at 72 °C. Unpurified PCR products were subsequently sent for bidirectional Sanger sequencing to BGI (Hong Kong, China). The sequences were edited for base-calling errors and assembled using Geneious R7 (version 7.1.3, Biomatters Ltd.) and all new sequences were submitted to GenBank (see accession numbers under each species).

We compared the newly obtained DNA barcodes from Georgian specimens with COI sequences present in GenBank (https://www.ncbi.nlm.nih.gov/genbank/) and in BOLD systems (http://www.boldsystems.org/index.php). We provided the Barcode Index Number (BIN) (Ratnasingham and Hebert 2013) for the sequenced taxa and for their nearest neighbour in BOLD systems when they had a BIN.

## Results

A total of 2,312 specimens were studied. We reported 357 different species belonging to 78 genera, with 40 species recorded from Georgia for the first time. Moreover, we were able to sequence DNA barcodes for 238 specimens (GenBank accession numbers for each species are provided under the section Genetics) representing 74 species from this country (see Suppl. material 1: Figure S1). The species are listed in alphabetic order.

### Checklist of Syrphidae of Georgia

***Anasimyia contracta* Torp & Claußen, 1980**

**New records.** GEORGIA • 1♀; L63, 23 Jul 2001, J.-H. Stuke leg.; ZFMK-DIP-00058254.

**Distribution.** Western Palaearctic.

**Remarks.** Reported for Georgia for the first time.

***Anasimyia lineata* (Fabricius, 1787)**

**Reference.**Gudjabidze (2002) as *Helophilus lineatus* (Fallén, 1787) [sic].

**New records.** GEORGIA • 1♀; L46, 24 Jul 2001, J.-H. Stuke leg.; ZFMK-DIP-00058023.

**Distribution.** Palaearctic.

***Anasimyia lunulata* (Meigen, 1822)**

**Reference.**Peck (1988); Barkalov and Mutin (2018).

**Distribution.** Northern and Central Europe, British Isles, and European parts of Russia.

***Anasimyia transfuga* (Linnaeus, 1758)**

**Reference.**Peck (1988).

**Distribution.** Northern and Central Europe, Balkan Peninsula and eastwards to European Russia, and as far as central Siberia.

***Baccha elongata* (Fabricius, 1775)**

**Reference.**Tóth (1986) as Baccha elongata (Fabricius, 1775) and as *Baccha obscuripennis* Meigen, 1822; Peck (1988); Peck (1988) as *B. obscuripennis*; Gudjabidze (2002) as *Baccha elongate* (Fallen, 1817) [sic]; Gudjabidze (2002) as *B. obscuripennis*; Seropian (2013a) as field observation.

**New records.** GEORGIA • 1♀; L1, 16 Jun 2018, S. Bot leg.; • 1♀; L6, 19 Jun 2018, S. Bot leg.; • 1♂; L20, 30 Jun 2018, S. Bot leg.; • 2♂; L24, 17 Jul 2018, X. Mengual leg.; ZFMK-DIP-00054019 = ZFMK-TIS-8000955, ZFMK-DIP-00054020 = ZFMK-TIS-8000964; • 1♀; L25, 18 Jul 2018, X. Mengual leg.; ZFMK-DIP-00053673 = ZFMK-TIS-8005557; • 1♂; L26, 18 Jul 2018, X. Mengual; ZFMK-DIP-00053672 = ZFMK-TIS-8005549.

**Genetics.** We sequenced three specimens (MN621895, MN621896, MN621897), which have identical COI barcode sequences, and represent the first barcodes of this species from Georgia. The BIN for these specimens is BOLD:ABA3006. The average distance within this BIN is 0.19% (p-dist) and the maximum distance is 1.99% (p-dist). The nearest neighbour in BOLD systems is the Nearctic species *Baccha cognata* Loew, 1863 (BOLD:AAG4682).

**Distribution.** Western Palaearctic.

**Remarks.** In recent literature (Speight 2018a) *Baccha obscuripennis* Meigen, 1822 is considered a junior synonym of *Baccha elongata*. We follow this synonym here.

***Brachyopa bicolor* (Fallén, 1817)**

**Reference.**Peck (1988).

**Distribution.** Palaearctic.

***Brachyopa insensilis* Collin, 1939**

**Reference.**Peck (1988); Barkalov and Mutin (2018).

**Distribution.** Western and Central Palaearctic.

***Brachyopa pilosa* Collin, 1939**

**Reference.**Peck (1988).

**New records.** GEORGIA • 1♀; L3, 17 Jun 2018, S. Bot leg.; 1♂; L8, 20 Jun 2018, S. Bot leg.

**Distribution.** Northern and Central Europe, and European parts of Russia.

***Brachypalpoides lentus* (Meigen, 1822)**

**Reference.**Levitin (1962) as *Zelima lenta* Mg.; Peck (1988); Gudjabidze (2002) as *Xylota lenta* Linnaeus, 1758 [sic]; Barkalov and Mutin (2018).

**Distribution.** Europe, European parts of Russia, and Asia Minor.

**Brachypalpus (Brachypalpus) chrysites Egger, 1859**

**Reference.**Peck (1988); Gudjabidze (2002); Mutin and Ichige (2018) from West Transcaucasia.

**New records.** GEORGIA • 1♂ 1♀; L4, 18 Jun 2018, S. Bot leg.

**Distribution.** Mountainous parts of central Europe and Pyrenees, Balkan Peninsula, Turkey, and European parts of Russia.

**Brachypalpus (Brachypalpus) nigrifacies Stackelberg, 1965**

**Reference.**Peck (1988); Gudjabidze (2002) as *Brachypalpus nigrifacies* Stackelberg, 1958 [sic]; Barkalov and Mutin (2018); Mutin and Ichige (2018).

**Distribution.** Transcaucasia.

***Caliprobola aurea* (Sack, 1910)**

**Reference.**Peck (1988) listed only from Azerbaijan; Speight (2018a).

**Distribution.** South of the Caucasus Mountains in Georgia and Azerbaijan.

***Caliprobola speciosa* (Rossi, 1790)**

**Reference.**Levitin (1962) as *Calliprobola specioza* Rossi [sic]; Peck (1988); Gudjabidze (2002).

**New records.** GEORGIA • 1♂; L3, 17 Jun 2018, S. Bot leg.; *1; L8, 20 Jun 2018, S. Bot obs.

**Distribution.** Palaearctic.

***Callicera aenea* (Fabricius, 1777)**

**Reference.**Peck (1988); Gudjabidze (2002).

**Distribution.** Palaearctic.

**Remarks.**Speight (2018a) affirmed that the range of this species needs a reassessment because it can be confused with *Callicera aurata* (Rossi, 1790).

***Callicera aurata* (Rossi, 1790)**

**Reference.**Barkalov and Mutin (2018) as *C. aurata*; Barkalov and Mutin (2018) as *Callicera zhelochovtsevi* Zimina, 1982; Speight (2018a).

**Distribution.** Western Palaearctic, including Transcaucasia.

**Remarks.**Speight (1991) synonymised *C. zhelochovtsevi* under *C. aurata* based on the study of specimens determined by Zimina. Distribution requires reassessment due to the confusion with *C. aenea* (Speight 2018a).

***Callicera rohdendorfi* Zimina, 1982**

**Reference.**Peck (1988); Barkalov and Mutin (2018); Speight (2018a).

**Distribution.** Crimea and Georgia.

**Remarks.** The taxonomic status of *C. rohdendorfi* is unclear. Speight (1991) suggested that this species is the same species as *Callicera macquarti* Rondani, 1844, but he did not see any material identified as *C. rohdendorfi*. Without further evidence, we keep it as a valid species.

***Ceriana conopsoides* (Linnaeus, 1758)**

**Reference.**Levitin (1962) as *Cerioides conopoides* L. [sic]; Peck (1988); Gudjabidze (2002) as *Ceriana caucasica* (Paramonov, 1925) [sic]; Barkalov and Mutin (2018).

**New records.** GEORGIA • 1♀; L64, 23 Jul 2001, J.-H. Stuke leg.; ZFMK-DIP-00058047.

**Distribution.** Palaearctic.

**Remarks.**Van Steenis et al. (2016) synonymised *Ceriana caucasica* (Paramonov, 1927) (Paramonov 1927a) under *C. conopsoides*.

**Chalcosyrphus (Xylotina) nemorum (Fabricius, 1805)**

**Reference.**Peck (1988).

**New records.** GEORGIA • 2♂; L24, 17 Jul 2018, X. Mengual leg.; ZFMK-DIP-00053787 = ZFMK-TIS-8005576, ZFMK-DIP-00053789; • 2♂ 2♀; L25, 18 Jul 2018, X. Mengual leg.; ZFMK-DIP-00054079, ZFMK-DIP-00054080 = ZFMK-TIS-8000966, ZFMK-DIP-00053791, ZFMK-DIP-00053792; • 2♂; L26, 18 Jul 2018, X. Mengual leg.; ZFMK-DIP-00053788 = ZFMK-TIS-8005568, ZFMK-DIP-00053790; • 1♂; L31, 23 Jul 2018, A. Reimann leg.; MTD-Dip-A-R-4523; • 2♂ 3♀; L71, 30 Jun–14 Jul 2018, malaise trap, GGBC-members leg.; ZFMK-TIS-8002740, ZFMK-TIS-8002741, ZFMK-TIS-8002742, ZFMK-TIS-8002743, ZFMK-DIP-00061261]; • 1♂; L71, 30 Jun–14 Jul 2018, malaise trap, GGBC-members leg.; MTD-Dip-A-R-4564; 2♀; L71, 30 Jun–14 Jul 2018, malaise trap, GGBC-members leg.; MTD-Dip-A-R-4593; • 1♀; L72, 29 Jun–13 Jul 2018, malaise trap, GGBC-members leg.; ZFMK-TIS-8002772.

**Genetics.** We obtained seven COI barcodes for this species (MN621898, MN621899, MN621900, MN621901, MN621902, MN621903, MN621904), all with identical COI sequence (0% p-dist). The BIN for these specimens is BOLD:AAG6762, with an average distance of 0.26% and a maximum distance of 2.41%. The nearest neighbour in BOLD systems is the Nearctic species Chalcosyrphus (Xylotomima) anomalus (Shannon, 1925) (BOLD:AAQ2056).

**Distribution.** Holarctic.

**Chalcosyrphus (Xylotodes) eunotus (Loew, 1873)**

**Reference.**Peck (1988) listed it from Armenia; Speight (2018a).

**Distribution.** Western Palaearctic.

**Chalcosyrphus (Xylotodes) piger (Fabricius, 1794)**

**Reference.**Peck (1988); Gudjabidze (2002) as *Xylota pigra* Fabricius, 1777 [sic].

**New records.** GEORGIA • 1♂; L3, 17 Jun 2018, S. Bot leg.; • 1♂; L5, 18 Jun 2018, S. Bot leg.

**Distribution.** Holarctic.

**Chalcosyrphus (Xylotomima) pannonicus (Oldenberg, 1916)**

**Reference.**Peck (1988).

**New records.** GEORGIA • 1♂; L71, 30 Jun–14 Jul 2018, malaise trap, GGBC-members leg.; ZFMK-TIS-8002758.

**Genetics.** We sequenced one specimen of this taxon (MN621905). BOLD has no data for *C. pannonicus*, so this sequence is the first one to be registered in BOLD. The closest taxon in BOLD systems is the Nearctic species Chalcosyrphus (Xylotomima) anthreas (Walker, 1849), BOLD:AAY8777.

**Distribution.** Poland, Carpathian Mountains, Balkan Peninsula, Greece, and Transcaucasia.

**Chalcosyrphus (Xylotomima) rufipes (Loew, 1873)**

**Reference.**Peck (1988); Speight (2018a).

**Distribution.** Palaearctic.

**Chalcosyrphus (Xylotomima) valgus (Gmelin, 1790)**

**Reference.**Tóth (1986) as *Xylotomima femoralis* (Linnaeus, 1758); Peck (1988) as Chalcosyrphus (Xylotomima) femoratus (Linnaeus, 1758); Barkalov and Mutin (2018).

**Distribution.** Palaearctic.

**Remarks.**Thompson et al. (1982) explained that the concept of Loew (1854) for “*femorata* Linnaeus” is a junior synonym of *Musca valga* Gmelin, 1790.

**Cheilosia (Cheilosia) abagoensis Skufjin, 1979**

**New records.** GEORGIA • 1♂; L12, 24 Jun 2018, S. Bot leg.; • 1♀; L19, 29 Jun 2018, S. Bot leg.

**Distribution.** Transcaucasia.

**Remarks.** Described from Krasnodar region and reported from this area by Barkalov (1993) and Barkalov and Mutin (2018). Reported for Georgia for the first time.

**Cheilosia (Cheilosia) aerea Dufour, 1848**

**Reference.**Peck (1988) as *Cheilosia zetterstedti* Becker, 1894; Gudjabidze (2002) as *C. zetterstendti* Becker, 1921 [sic]; Barkalov and Mutin (2018); Speight (2018a).

**Distribution.** Western Palaearctic.

**Remarks.**Claußen and Thompson (1996) synonymised *C. zetterstedti* under *C. aerea*.

**Cheilosia (Cheilosia) albipila Meigen, 1838**

**Reference.**Tóth (1986); Peck (1988); Gudjabidze (2002) as *Cheilosia albipina* Meigen, 1822 [sic].

**Distribution.** Western and Central Palaearctic.

**Cheilosia (Cheilosia) albitarsis (Meigen, 1822)**

**Reference.**Peck (1988); Gudjabidze (2002).

**New records.** GEORGIA • 1♀; L1, 15 Jun 2018, S. Bot leg.; • 1♀; L1, 16 Jun 2018, S. Bot leg.; • 1♂; L10, 22 Jun 2018, S. Bot leg.; • 1♂ 3♀; L11, 29 Jun 2018, S. Bot leg.; • 1♀; L19, 29 Jun 2018, S. Bot leg.; • 2♀; L20, 30 Jun 2018, S. Bot leg.; • 1♂; L19, 1 Jul 2018, S. Bot leg.; • 1♀; L21, 2 Jul 2018, S. Bot leg.

**Distribution.** Holarctic.

**Remarks.** Geographic distribution needs reassessment as old species records need reconfirmation after Doczkal (2000a).

**Cheilosia (Cheilosia) bergenstammi Becker, 1894**

**Reference.**Peck (1988); Barkalov and Mutin (2018).

**Distribution.** Western Palaearctic.

**Cheilosia (Cheilosia) bracusi Vujić & Claußen, 1994**

**Nem records.** GEORGIA • 2♀; L4, 18 Jun 2018, S. Bot leg; • 2♀; L6, 19 Jun 2018, S. Bot leg.

**Distribution.** Southern and Central Europe, and Balkan Peninsula.

**Remarks.** Reported for Georgia for the first time.

**Cheilosia (Cheilosia) brunnipennis Becker, 1894**

**Reference.**Peck (1988) as *Cheilosia sareptana* Becker, 1894.

**Distribution.** Western Palaearctic.

**Remarks.**Vujić (1996) synonymised *C. sareptana* under *C. brunnipennis*.

**Cheilosia (Cheilosia) canicularis (Panzer, 1801)**

**Reference.**Radde (1899); Levitin (1962); Peck (1988); Gudjabidze (2002) as *Cheilosia canicularis* Panzer, 1798 [sic].

**New records.** GEORGIA • 1♂; L16, 27 Jun 2018, S. Bot leg.; • 1♂ 1♀; L20, 30 Jun 2018, S. Bot leg.; • 2♂ 2♀; L20, 1 Jul 2018, S. Bot leg.; • 1♀; L20, 2 Jul 2018, S. Bot leg.; • 3♂; L21, 3 Jul 2018, S. Bot leg.; • 1♂; L24, 17 Jul 2018, X. Mengual leg.; ZFMK-DIP-00053871; • 1♂; L29, 19 Jul 2018, X. Mengual leg.; ZFMK-DIP-00053872; • 1♀; L31, 21 Jul 2018, J. Astrin leg.; ZFMK-TIS-8000118; • 2♂ 1♀; L31, 20 Jul 2018, X. Mengual leg.; ZFMK-DIP-00053873 = ZFMK-TIS-8005511, ZFMK-DIP-00053976 = ZFMK-TIS-8003440, ZFMK-DIP-00053897; • 1♂; L33, 22 Jul 2018, X. Mengual leg.; ZFMK-DIP-00053874; • 1♂; L33, 23 Jul 2018, X. Mengual leg.; ZFMK-DIP-00054023 = ZFMK-TIS-8000982; • 1♂; L34, 23 Jul 2018, J. and B. Thormann leg.; ZFMK-TIS-8004303; • 10♂ 3♀; L35, 24 Jul 2018, X. Mengual leg.; ZFMK-DIP-00053875, ZFMK-DIP-00053876, ZFMK-DIP-00053877, ZFMK-DIP-00053878, ZFMK-DIP-00053879, ZFMK-DIP-00053880, ZFMK-DIP-00053881, ZFMK-DIP-00053882, ZFMK-DIP-00053883, ZFMK-DIP-00053975 = ZFMK-TIS-8003452, ZFMK-DIP-00053898, ZFMK-DIP-00053901, ZFMK-DIP-00053906; • 4♂ 3♀; L36, 24 Jul 2018, X. Mengual leg.; ZFMK-DIP-00053884, ZFMK-DIP-00053886, ZFMK-DIP-00053887, ZFMK-DIP-00053893, ZFMK-DIP-00053899, ZFMK-DIP-00053900 = ZFMK-TIS-8005518, ZFMK-DIP-00053905; • 1♂; L37, 25 Jul 2018, B. Thormann leg.; ZFMK-TIS-8004124; • 8♂ 2 ♀;L37, 25 Jul 2018, X. Mengual leg.; ZFMK-DIP-00053885, ZFMK-DIP-00053888, ZFMK-DIP-00053889, ZFMK-DIP-00053890, ZFMK-DIP-00053891, ZFMK-DIP-00053892, ZFMK-DIP-00053896, ZFMK-DIP-00053977 = ZFMK-TIS-8003421, ZFMK-DIP-00053903, ZFMK-DIP-00053904; • 2♂ 1♀; L38, 25 Jul 2018, X. Mengual leg.; ZFMK-DIP-00053894, ZFMK-DIP-00053895, ZFMK-DIP-00053902; • 2♂; L69, 18 Jul 2018, A. Reimann leg.; MTD-Dip-A-R-4506, ZFMK-TIS-8002665; • 1♀; L71, 30 Jun–14 Jul 2018, malaise trap, GGBC-members leg.; ZFMK-TIS-8002757.

**Genetics.** The GenBank accession numbers for the seven sequenced specimens are MN621906, MN621907, MN621908, MN621909, MN621910, MN621911, MN621912. Our newly obtained DNA barcodes were virtually identical (0–0.1% p-dist). The BIN for this taxon is BOLD:ACI2500. Identification via DNA barcodes is not straightforward as *C. canicularis* and its nearest neighbour in BOLD systems, *Cheilosia himantopa* (Panzer, 1798), differ only 1.28% p-dist.

**Distribution.** Central Europe and Turkey.

**Remarks.** Geographic distribution needs reassessment after Stuke and Claußen (2000) reinstated *C. himantopa* (Panzer, 1798), a closely similar species of *C. canicularis*.

**Cheilosia (Cheilosia) chloris (Meigen, 1822)**

**Reference.**Gudjabidze (2002).

**Distribution.** Western and Central Palaearctic, into Siberia.

**Cheilosia (Cheilosia) flavipes (Panzer, 1798)**

**Reference.**Peck (1988); Barkalov and Mutin (2018).

**Distribution.** Western and Central Palaearctic, into Siberia.

**Cheilosia (Cheilosia) fraterna (Meigen, 1830)**

**Reference.**Peck (1988).

**Distribution.** Western and Central Palaearctic, into Siberia.

**Cheilosia (Cheilosia) gigantea (Zetterstedt, 1838)**

**Reference.**Peck (1988); Gudjabidze (2002) as *Cheilosia gigantean* Zetterstendt, 1843 [sic]; Barkalov and Mutin (2018); Speight (2018a).

**New records.** GEORGIA • 1♂ 1♀; L4, 18 Jun 2018, S. Bot leg.;• 2♂; L6, 19 Jun 2018, S. Bot leg.; • 1♀; L8, 20 Jun 2018, S. Bot leg.; • 1♂; L10, 22 Jun 2018, S. Bot leg.; • 1♂; L12, 24 Jun 2018, S. Bot leg.; • 1♂; L15, 26 Jun 2018, S. Bot leg.; • 1♂; L20, 1 Jul 2018, S. Bot leg.

**Distribution.** Palaearctic, except Mediterranean Basin.

**Cheilosia (Cheilosia) grossa (Fallén, 1817)**

**Reference.**Gudjabidze (2002) as *Cheilosia grossa* Meigen, 1822 [sic].

**Distribution.** Palaearctic and Uttah Pradesh in northern India.

**Cheilosia (Cheilosia) impressa Loew, 1840**

**Reference.**Peck (1988); Gudjabidze (2002) as *Cheilosia impressa* Loew, 1848 [sic]; Speight (2018a).

**New records.** GEORGIA • 1♀; L3, 17 Jun 2018, S. Bot leg.; • 1♂; L10, 22 Jun 2018, S. Bot leg.; • 2♀; L11, 23 Jun 2018, S. Bot leg.; • 1♀; L11, 29 Jun 2018, S. Bot leg.; • 1♂ 2♀; L12, 24 Jun 2018, S. Bot leg.; • 1♂ 1♀; L14, 25 Jun 2018, S. Bot leg.; • 2♂ 2♀; L15, 26 Jun 2018, S. Bot leg.; • 4♂; L19, 29 Jun 2018, S. Bot leg.; • 1♂ 2♀; L20, 30 Jun 2018, S. Bot leg.; • 1♀; L20, 1 Jul 2018, S. Bot leg.; • 2♀; L57, 3 Aug 2001, J.-H. Stuke leg.; ZFMK-DIP-00058257, ZFMK-DIP-00058258; • 1♀; L31, 23 Jul 2018, A. Reimann leg.; MTD-Dip-A-R-4530.

**Distribution.** Palaearctic.

**Cheilosia (Cheilosia) lasiopa Kowarz, 1855**

**Reference.**Tóth (1986) as *Cheilosia honesta* Rondani, 1868.

**Distribution.** Europe, Caucasus, and European parts of Russia.

**Remarks.**Peck (1988) listed Cheilosia (Cheilosia) lasiopa Kowarz, 1855 as junior synonym of *C. honesta*. Later, Claußen and Thompson (1996) considered *C. honesta* Rondani as junior synonym of Cheilosia (Cheilosia) barbata Loew, 1857 and the taxon known as *C. honesta* from other authors as synonym of Cheilosia (Cheilosia) lasiopa Kowarz, 1855. Speight (2018a) mentioned that Cheilosia (Cheilosia) lasiopa Kowarz, 1855 appears as *Cheilosia honesta* in recent literature. Thus, it seems reasonable that the taxon identified by Tóth (1986) as *Cheilosia honesta* was, indeed, *C. lasiopa*.

**Cheilosia (Cheilosia) latifrons (Zetterstedt, 1843)**

**Reference.**Peck (1988) as *Cheilosia intonsa* Loew, 1857.

**Distribution.** Palaearctic.

**Remarks.**Speight and Lucas (1992) synonymised *C. intonsa* under *C. latifrons*.

**Cheilosia (Cheilosia) lenis Becker, 1894**

**Reference.**Peck (1988) as *Cheilosia omissa* Becker, 1894.

**New records.** GEORGIA • 6♀; L4, 18 Jun 2018, S. Bot leg.; • 2♂; L5, 18 Jun 2018, S. Bot leg.; • 3♀; L6, 19 Jun 2018, S. Bot leg.; • 1♂; L7, 19 Jun 2018, S. Bot leg.; • 1♂; L8, 20 Jun 2018, S. Bot leg.; • 2♂ 3♀; L10, 22 Jun 2018, S. Bot leg.; • 1♂; L11, 23 Jun 2018, S. Bot leg.; • 1♂ 1♀; L15, 26 Jun 2018, S. Bot leg.; • 1♀; L16, 27 Jun 2018, S. Bot leg.; • 1♂; L17, 28 Jun 2018, S. Bot leg.; • 1♂ 3♀; L19, 29 Jun 2018, S. Bot leg.; • 2♂ 1♀; L20, 30 Jun 2018, S. Bot leg.; • 2♀; L20, 1 Jul 2018, S. Bot leg.; • 1♀; L20, 2 Jul 2018, S. Bot leg.

**Distribution.** Europe and European parts of Russia.

**Remarks.**Claußen and Speight (2007) synonymised *C. omissa* under *C. lenis*.

**Cheilosia (Cheilosia) melanopa (Zetterstedt, 1843)**

**New records.** GEORGIA • 5♂; L4, 18 Jun 2018, S. Bot leg.; • 1♀; L5, 18 Jun 2018, S. Bot leg.; • 1♂ 1♀; L6, 19 Jun 2018, S. Bot leg.; • 1♂ 2♀; L7, 19 Jun 2018, S. Bot leg.; • 2♂ 1♀; L12, 24 Jun 2018, S. Bot leg.; • 2♂ 2♀; L14, 25 Jun 2018, S. Bot leg.; • 2♀; L16, 27 Jun 2018, S. Bot leg.; • 1♂; L19, 29 Jun 2018, S. Bot leg.; • 1♀; L20, 30 Jun 2018, S. Bot leg.; • 1♀; L52, 30 Jul 2001, J.-H. Stuke leg.; ZFMK-DIP-00058102; • 1♀; L55, 31 Jul 2001, J.-H. Stuke leg.; ZFMK-DIP-00058103; • 1♀; L70, 30 Jun–14 Jul 2018, malaise trap, GGBC-members leg.; ZFMK-TIS-8002723.

**Genetics.** We sequenced one specimen (MN621914) and the obtained sequence is very similar (98.87%) to the sequence of *C. melanopa* in GenBank (AY533360 from Yugoslavia; BIN = BOLD:AAW3655). BOLD has a second BIN for *C. melanopa* (BOLD:ACE3977 with specimens from Central and Northern Europe), which is very close to BOLD:AAW3655 (2.36% p-dist).

**Distribution.** Europe.

**Remarks.**Barkalov (1993) reported this species from Northern Caucasus and Armenia and mentioned that this taxon was polymorphic, with one morph having almost black legs and black pilosity on scutum and scutellum, and a second pale morph with legs partly yellow and mostly yellow pilosity on scutum and scutellum. We sequenced only a single specimen, but there are two BINs in BOLD systems with a relatively high uncorrected pairwise distance (2.36% p-dist; in the range of the p-distance among different species in other species pairs), which might represent these two morphs. Reported for Georgia for the first time.

**Cheilosia (Cheilosia) melanura Becker, 1894**

**Reference.**Peck (1988); Gudjabidze (2002) as *Cheilosia melanura* Becker, 1921 [sic]; Speight (2018a).

**Distribution.** Mountain ranges in Central Europe, Balkans, and Caucasus Mountains, east to the Baikal Region.

**Cheilosia (Cheilosia) mutabilis (Fallén, 1817)**

**Reference.**Radde (1899); Tóth (1986); Peck (1988) as *C. mutabilis* and as *Cheilosia ruralis* (Meigen, 1822); Gudjabidze (2002) as *C. mutabilis* and as *C. ruralis*; Barkalov and Mutin (2018).

**New records.** GEORGIA • 1♀; L6, 19 Jun 2018, S. Bot leg.; • 4♂ 13♀; L57, 3 Aug 2001, J.-H. Stuke leg.; ZFMK-DIP-00058118, ZFMK-DIP-00058119, ZFMK-DIP-00058120, ZFMK-DIP-00058104, ZFMK-DIP-00058105, ZFMK-DIP-00058106, ZFMK-DIP-00058107, ZFMK-DIP-00058108, ZFMK-DIP-00058109, ZFMK-DIP-00058110, ZFMK-DIP-00058111, ZFMK-DIP-00058112, ZFMK-DIP-00058113, ZFMK-DIP-00058114, ZFMK-DIP-00058115, ZFMK-DIP-00058116, ZFMK-DIP-00058117.

**Distribution.** Western and Central Palaearctic, into western Siberia.

**Remarks.**Claußen and Speight (1999) synonymised *C. ruralis* under *C. mutabilis*.

**Cheilosia (Cheilosia) pagana (Meigen, 1822)**

**Reference.**Peck (1988); Gudjabidze (2002); Barkalov and Mutin (2018).

**Distribution.** Palaearctic.

**Cheilosia (Cheilosia) paragigantea Barkalov, 1993**

**New records.** GEORGIA • 2♀; L6, 19 Jun 2018, S. Bot leg.; • 1♀; L16, 27 Jun 2018, S. Bot leg.; • 1♀; L19, 29 Jun 2018, S. Bot leg.

**Distribution.** Transcaucasia.

**Remarks.**Barkalov (1993) described this species without indicating any type material, but mentioned that among the type material there were some specimens identified as *C. gigantea* by Stackelberg and Richter (1968). In his identification key, Barkalov (1993) stated that *C. paragigantea* is found in the Lesser Caucasus, but Barkalov and Mutin (2018) reported *C. paragigantea* only from Northern Caucausus. Thus, following Barkalov and Mutin (2018) as the most recent publication, we report this species for Georgia for the first time.

**Cheilosia (Cheilosia) proxima (Zetterstedt, 1843)**

**Reference.**Tóth (1986); Peck (1988).

**New records.** GEORGIA • 1♀; L6, 19 Jun 2018, S. Bot leg.; • 1♀; L11, 23 Jun 2018, S. Bot leg.; • 1♀; L16, 27 Jun 2018, S. Bot leg.; • 1♂; L19, 29 Jun 2018, S. Bot leg.; • 1♂; L20, 1 Jul 2018, S. Bot leg.; • 1♂; L20, 2 Jul 2018, S. Bot leg; • 1♂; L21, 3 Jul 2018, S. Bot leg.

**Distribution.** Palaearctic.

**Cheilosia (Cheilosia) pseudogrossa Stackelberg, 1968**

**Reference.**Gudjabidze (2002) as *Cheilosia pseudogrossa* Stackelberg, 1956 [sic].

**Distribution.** Transcaucasia.

**Remarks.**Stackelberg (1968) described the species from the Northern Caucasus (Teberda, Teberdinsky State Natural Biosphere Reserve), and Gudjabidze (2002) reported it from Tsebelda (Abkhazia region).

**Cheilosia (Cheilosia) rhynchops Egger, 1860**

**New records.** GEORGIA • 1♂; L5, 18 Jun 2018, S. Bot leg.; • 1♀; L6, 19 Jun 2018, S. Bot leg.; • 3♀; L11, 29 Jun 2018, S. Bot leg.; • 2♂; L12, 24 Jun 2018, S. Bot leg.; • 1♂; L14, 25 Jun 2018, S. Bot leg.; • 1♀; L18, 28 Jun 2018, S. Bot leg.; • 2♀; L20, 30 Jun 2018, S. Bot leg.; • 1♀; L21, 2 Jul 2018, S. Bot leg.

**Distribution.** Europe and Transcaucasia.

**Remarks.** Reported from the Northern Caucasus by Barkalov (1993), and subsequently by Barkalov and Mutin (2018). Reported for Georgia for the first time.

**Cheilosia (Cheilosia) schnabli Becker, 1894**

**Reference.**Peck (1988); Gudjabidze (2002) as *Cheilosia schnabli* Becker, 1921 [sic]; Speight (2018a) listed it only from Dagestan in the Caucasus.

**New records.** GEORGIA • 4♂ 2♀; L19, 29 Jun 2018, S. Bot leg.; • 1♂; L20, 30 Jun 2018, S. Bot leg.; • 3♂ 1♀; L20, 1 Jul 2018, S. Bot leg.; • 1♂; L21, 2 Jul 2018, S. Bot leg.; • 1♂; L21, 3 Jul 2018, S. Bot leg.; • 1♂ 1♀; L31, 20 Jul 2018, X. Mengual leg.; ZFMK-DIP-00053907 = ZFMK-TIS-8005512, ZFMK-DIP-00053909 = ZFMK-TIS-8005522; • 1♂; L35, 24 Jul 2018, X. Mengual leg.; ZFMK-DIP-00053908 = ZFMK-TIS-8005519; • 1♀; L36, 24 Jul 2018, X. Mengual leg.; ZFMK-DIP-00054026 = ZFMK-TIS-8000996.

**Genetics.** The GenBank accession numbers for the three sequenced specimens are MN621915, MN621916, MN621917. The BIN for these specimens is BOLD:ADX7783 and our sequences are very similar (98.62%) with the single previous record of this species in GenBank (LT707517 from Russia).

**Distribution.** Balkan Peninsula, Transcaucasia, and Kazakhstan.

**Cheilosia (Cheilosia) teberdensis Barkalov, 1993**

**New records.** GEORGIA • 1♀; L14, 25 Jun 2018, S. Bot leg.; • 1♂; L20, 1 Jul 2018, S. Bot leg.

**Distribution.** Transcaucasia.

**Remarks.**Barkalov (1993) described this species from Northern Caucasus and, consequently, Barkalov and Mutin (2018) listed it. Reported for Georgia for the first time.

**Cheilosia (Cheilosia) transcaucasica Stackelberg, 1960**

**Reference.**Peck (1988) listed it only from Armenia and Azerbaijan; Gudjabidze (2002) as *Cheilosia transcaucasika* Stackelberg, 1956 [sic]; Barkalov and Mutin (2018).

**New records.** GEORGIA • 1♀; L11, 23 Jun 2018, S. Bot leg.; • 1♂ 1♀; L12, 24 Jun 2018, S. Bot leg.; • 2♂ 4♀; L19, 29 Jun 2018, S. Bot leg.; • 2♂ 3♀; L20, 1 Jul 2018, S. Bot leg.; • 1♀; L21, 3 Jul 2018, S. Bot leg.; • 1♀; L53, 1 Aug 2001, J.-H. Stuke leg.; ZFMK-DIP-00058101.

**Distribution.** Transcaucasia.

**Cheilosia (Cheilosia) urbana (Meigen, 1822)**

**Reference.**Tóth (1986) as *Cheilosia praecox* (Zetterstedt, 1843); Peck (1988) as *Cheilosia praecox* (Zetterstedt, 1843).

**New records.** GEORGIA • 1♂; L5, 18 Jun 2018, S. Bot leg.; • 2♂ 3♀; L12, 24 Jun 2018, S. Bot leg.; • 1♂ 1♀; L14, 25 Jun 2018, S. Bot leg.; • 5♂ 2♀; L15, 26 Jun 2018, S. Bot leg.; • 1♀; L16, 27 Jun 2018, S. Bot leg.; • 1♀; L18, 28 Jun 2018, S. Bot leg.; • 1♀; L19, 29 Jun 2018, S. Bot leg.; • 2♂ 2♀; L20, 30 Jun 2018, S. Bot leg.; • 3♂ 1♀; L20, 1 Jul 2018, S. Bot leg.; • 1♂; L21, 2 Jul 2018, S. Bot leg.

**Distribution.** Western Palaearctic.

**Remarks.**Speight et al. (1998) suggested *C. praecox* as a junior synonym of *C. urbana*, and Claußen and Speight (1999) synonymised *C. ruralis* under *C. urbana*.

**Cheilosia (Cheilosia) variabilis (Panzer, 1798)**

**Reference.**Gudjabidze (2002).

**New records.** GEORGIA • 1♂; L15, 26 Jun 2018, S. Bot leg.; • 1♀; L18, 28 Jun 2018, S. Bot leg.

**Distribution.** Western and Central Palaearctic, into western Siberia.

**Cheilosia (Cheilosia) velutina Loew, 1840**

**Reference.**Peck (1988); Gudjabidze (2002) as *Cheilosia velutina* Loew, 1848 [sic].

**Distribution.** Palaearctic.

**Cheilosia (Cheilosia) vernalis (Fallén, 1817)**

**Reference.**Peck (1988); Gudjabidze (2002); Barkalov and Mutin (2018).

**New records.** GEORGIA • 1♂ 1♀; L54, 2 Aug 2001, J.-H. Stuke leg.; ZFMK-DIP-00058263, ZFMK-DIP-00058264; • 2♂ 2♀; L55, 31 Jul 2001, J.-H. Stuke leg.; ZFMK-DIP-00058259, ZFMK-DIP-00058260, ZFMK-DIP-00058261, ZFMK-DIP-00058262.

**Distribution.** Palaearctic.

**Cheilosia (Cheilosia) vulpina (Meigen, 1822)**

**Reference.**Levitin (1962) as *Cheilosia conops* Becker, 1894; Peck (1988) as *Cheilosia conops*.

**Distribution.** Western and Central Palaearctic, into western Siberia.

**Remarks.**Claußen and Speight (1988) synonymised *C. conops* under *C. vulpina*.

**Cheilosia (Convocheila) cumanica Szilády, 1938**

**Reference.**Peck (1988) as *Cheilosia verae* Stackelberg, 1968; Gudjabidze (2002) as *C. verae* Stackelberg, 1956 [sic]; Barkalov and Mutin (2018) as *C. verae*.

**New records.** GEORGIA • 1♀; L4, 18 Jun 2018, S. Bot leg.; • 1♀; L5, 18 Jun 2018, S. Bot leg.; • 2♀; • 1♂; L12, 24 Jun 2018, S. Bot leg; L16, 27 Jun 2018, S. Bot leg.; • 2♂ 2♀; L19, 29 Jun 2018, S. Bot leg.; • 1♀; L20, 30 Jun 2018, S. Bot leg.; • 3♂; L20, 1 Jul 2018, S. Bot leg.; • 1♂; L20, 2 Jul 2018, S. Bot leg.; • 1♂ 1♀; L57, 3 Aug 2001, J.-H. Stuke leg.; ZFMK-DIP-00058068, ZFMK-DIP-00058067.

**Distribution.** Balkan Peninsula, Carpathians Mountains, Iran, and Transcaucasia.

**Remarks.**Brădescu (1991) synonymised *C. verae* under *C. cumanica*.

**Cheilosia (Convocheila) laticornis Rondani, 1857**

**Reference.**Peck (1988) as *Cheilosia latifacies* Loew, 1857; Gudjabidze (2002) as *Cheilosia latifacies* Loew, 1846 [sic]; Barkalov and Mutin (2018); Speight (2018a).

**Distribution.** Western and Central Europe, including Transcaucasia.

**Remarks.**Claußen and Thompson (1996) synonymised *C. latifacies* under *C. laticornis*.

**Cheilosia (Eucartosyrphus) flavissima Becker, 1894**

**Reference.**Peck (1988) as *Cheilosia pallipes* Loew, 1863.

**Distribution.** Palaearctic.

**Remarks.**Peck (1988) listed *C. flavissima* as synonym of *C. pallipes*, most likely following Doesburg (1959), but Claußen and Ståhls (2007) separated both taxa and stated that *C. pallipes* applies to specimens from North America.

**Cheilosia (Eucartosyrphus) ruffipes (Preyssler, 1793)**

**Reference.**Peck (1988) as *Cheilosia rufipes* (Preyssler, 1793) [sic] [= *Cheilosia ruffipes* (Preyssler, 1793)]; Gudjabidze (2002) as *Cheilosia soror* Zetterstendt, 1843 [sic].

**Distribution.** Palaearctic.

**Remarks.** In 1982, *Eristalis soror* Zetterstedt, 1843 (= *Cheilosia soror*) was synonymised with *Syrphus ruffipes* Preyssler, 1793 (= *Cheilosia ruffipes*) by Rozkošný et al. (1982). This synonymy was followed by subsequent authors, i.e., Peck (1988) listed *C. soror* as synonym of *C. ruffipes* (written as *rufipes* in Peck) and Vujić and Glumac (1994) listed *C. soror* as junior synonym of *C. rufipes* [sic] based on Peck (1988). More recently and without justification, Vujić (1996) accepted *C. soror* as a valid name, as explained in Vujić et al. (2002). In the more recent literature this taxon appears as *Cheilosia soror* (e.g., Van Veen 2010; Speight 2018a; Bot and Van der Meutter 2019), except in Barkalov and Mutin (2018) that is cited as *Cheilosia rufipes*. We keep the original spelling, *Cheilosia ruffipes*, as we think that the spelling by Peck (1988), *Cheilosia rufipes*, is either an error or an unjustified emendation of the name.

**Cheilosia (Eucartosyrphus) scutellata (Fallén, 1817)**

**Reference.**Levitin (1962); Peck (1988); Gudjabidze (2002); Barkalov and Mutin (2018).

**New records.** GEORGIA • 1♂; L3, 17 Jun 2018, S. Bot leg.; • 1♀; L57, 2 Aug 2001, J.-H. Stuke leg.; ZFMK-DIP-00058077; • 6♂ 2♀; L57, 3 Aug 2001, J.-H. Stuke leg.; ZFMK-DIP-00058069, ZFMK-DIP-00058070, ZFMK-DIP-00058072, ZFMK-DIP-00058073, ZFMK-DIP-00058074, ZFMK-DIP-00058075, ZFMK-DIP-00058071, ZFMK-DIP-00058076; • 2♀; L64, 23 Jul 2001, J.-H. Stuke leg.; ZFMK-DIP-00058078, ZFMK-DIP-00058079.

**Distribution.** Palaearctic.

**Cheilosia (Floccocheila) illustrata portschinskiana Stackelberg, 1960**

**Reference.**Portschinsky (1877) as *Cheilosia oestracea* Linnaeus, 1761 [sic]; Radde (1899) as *Cheilosia oestracea*; Peck (1988) listed this subspecies only from Armenia, its type locality as defined by Stackelberg (1960); Gudjabidze (2002) as *C. oestracae* (Linnaeus, 1758) [sic], as *C. portschinskiana* Stackelberg, 1956 [sic], and as *Eristalis oestraceus* Linnaeus, 1758 [sic]; Barkalov and Mutin (2018) listed this subspecies only from Northern Caucasus.

**New records.** GEORGIA • 1♂ 1♀; L4, 18 Jun 2018, S. Bot leg.; • 1♀; L5, 18 Jun 2018, S. Bot leg.; • 2♂; L14, 25 Jun 2018, S. Bot leg.; • *3; L14, 25 Jun 2018, S. Bot obs.; • 1♂; L16, 27 Jun 2018, S. Bot leg.; • *4♂; L17, 28 Jun 2018, S. Bot obs.; • 1♂ 1♀; L19, 29 Jun 2018, S. Bot leg.; • *4♀; L20, 30 Jun 2018, S. Bot obs.; • 1♀; L34, 22 Jul 2018, X. Mengual leg.; ZFMK-DIP-00053869 = ZFMK-TIS-8005510; • 3♀; L57, 2 Aug 2001, J.-H. Stuke leg.; ZFMK-DIP-00058098, ZFMK-DIP-00058099, ZFMK-DIP-00058100; • 3♂ 12♀; L57, 3 Aug 2001, J.-H. Stuke leg.; ZFMK-DIP-00058083, ZFMK-DIP-00058088, ZFMK-DIP-00058097, ZFMK-DIP-00058084, ZFMK-DIP-00058085, ZFMK-DIP-00058086, ZFMK-DIP-00058087, ZFMK-DIP-00058089, ZFMK-DIP-00058090, ZFMK-DIP-00058091, ZFMK-DIP-00058092, ZFMK-DIP-00058093, ZFMK-DIP-00058094, ZFMK-DIP-00058095, ZFMK-DIP-00058096.

**Genetics.** We sequenced one specimen (MN621913) of *C. illustrata portschinskiana* from Georgia, and its COI sequence has high similarity (99.85%) with previously published sequences of *C. illustrata illustrata* (Harris, 1779) from other Palaearctic countries. The BIN for this species is BOLD:AAK1092.

**Distribution.** Transcaucasia.

**Remarks.**Stackelberg (1960) stated that the taxon listed as *Cheilosia oestracea* (and its varieties b, c, d, e, and f) by Portschinsky (1877) was his new species *Cheilosia portschinskiana*. Our specimens fit the description of *Cheilosia illustrata portschinskiana* by Stackelberg (1960). According to Barkalov (1993), *C. illustrata portschinskiana* is the only subspecies of *Cheilosia illustrata* (Harris, 1779) occurring in the Caucasus.

The year of publication for *Cheilosia illustrata* was a convention. The original work by Harris (1776–1780) was published in five ‘decads’ or parts. Peck (1988) used the conventional date of 1780? with a question mark for decads 3, 4, and 5 based on Lisney (1960). Evenhuis (1997: page 343) established that the decad 4, where *Musca illustratus* is described on page 104, was dated as 1779 based on the latest date of the plates. Thus, the year of publication should be 1779, i.e., *Cheilosia illustrata* (Harris, 1779).

**Cheilosia (Montanocheila) alpina (Zetterstedt, 1838)**

**Reference.**Gudjabidze (2002) as *Cheilosia alpine* Zetterstendt, 1846 [sic].

**Distribution.** Germany, Northern Europe, Siberia, Mongolia to the Pacific.

**Remarks.**Gudjabidze (2002) reported this species from Lagodekhi, Batsara canyon (Georgia), but this material was not available to our study. We think it would be necessary to compare the material from Gudjabidze (2002) with specimens from northern latitudes to confirm the presence of this taxon in Georgia.

**Cheilosia (Montanocheila) caucasogenita Kuznetzov, 1997**

**Reference.**Kuznetzov (1997); Barkalov and Mutin (2018) listed it from Northern Caucasus and Armenia.

**Distribution.** Transcaucasia.

**Remarks.**Kuznetzov (1997) described this species based on specimens from Armenia, North Ossetia-Alania (Northern Caucasus, Russia) and Georgia.

**Cheilosia (Montanocheila) chrysocoma (Meigen, 1822)**

**Reference.**Gudjabidze (2002).

**Distribution.** Europe, European parts of Russia, and Siberia.

**Cheilosia (Montanocheila) pictipennis Egger, 1860**

**Reference.**Peck (1988); Gudjabidze (2002).

**Distribution.** Europe, European parts of Russia, and Siberia.

**Cheilosia (Taeniocheilosia) armeniaca Stackelberg, 1960**

**Reference.**Stackelberg (1960) described it from Armenia; Peck (1988) listed it only from Armenia; Gudjabidze (2002) as *Cheilosia armeniaca* Stackelberg, 1956 [sic]; Ståhls and Barkalov (2017).

**New records.** GEORGIA • 1♂; L13, 24 Jun 2018, S. Bot leg.

**Distribution.** Transcaucasia.

**Cheilosia (Taeniocheilosia) bakurianiensis Kuznetzov, 1987**

**Reference.**Kuznetzov (1987); Barkalov (1993) listed from Lesser Caucaus; Barkalov and Ståhls (1997).

**Distribution.** Only known from Georgia.

**Cheilosia (Taeniochilosia) grisella Becker, 1894**

**Reference.**Peck (1988); Gudjabidze (2002); Speight (2018a).

**Distribution.** Central Europe, Carpathians Mountains, Balkan Peninsula, and Transcaucasia.

**Remarks.**Barkalov (1993) stated that *C. grisella* does not occur in the Caucasus, and previous records of this taxon belong to *Cheilosia aenigmatosa* Barkalov, 1993. Ståhls and Barkalov (1997) listed *C. aenigmatosa* as a junior synonym of *C. pollinifacies* Stackelberg, 1968 and considered *C. grisella* as a valid species.

**Cheilosia (Taeniochilosia) impudens Becker, 1894**

**Reference.**Peck (1988); Speight (2018a).

**Distribution.** Europe and Transcaucasia.

**Cheilosia (Taeniochilosia) nigripes (Meigen, 1822)**

**Reference.**Tóth (1986); Peck (1988); Gudjabidze (2002).

**Distribution.** Palaearctic.

**Cheilosia (Taeniochilosia) pollinifacies Stackelberg, 1968**

**Reference.**Peck (1988) listed it from Northern Caucasus and Azerbaijan; Gudjabidze (2002) as *Cheilosia pollinifacies* Stackelberg 1956 [sic]; Ståhls and Barkalov (1997) listed it from Transcaucasia.

**New records.** GEORGIA • 2♀; L5, 18 Jun 2018, S. Bot leg.; • 1♂; L6, 19 Jun 2018, S. Bot leg.; • 3♂ 4♀; L12, 24 Jun 2018, S. Bot leg.; • 6♀; L15, 26 Jun 2018, S. Bot leg.; • 2♀; L18, 28 Jun 2018, S. Bot leg.; • 3♂ 8♀; L19, 29 Jun 2018, S. Bot leg.; • 1♂ 2♀; L20, 30 Jun 2018, S. Bot leg.; • 2♂ 1♀; L20, 1 Jul 2018, S. Bot leg.; • 1♂ 2♀; L55, 31 Jul 2001, J.-H. Stuke leg.; ZFMK-DIP-00058122, ZFMK-DIP-00058121, ZFMK-DIP-00058123.

**Distribution.** Transcaucasia.

**Cheilosia (Taeniochilosia) sahlbergi Becker, 1894**

**Reference.**Peck (1988); Speight (2018a).

**Distribution.** Europe, European parts of Russia, and Transcaucasia.

**Cheilosia (Taeniochilosia) vicina (Zetterstedt, 1849)**

**Reference.**Tóth (1986) as *Cheilosia nasutula* Becker 1894.

**Distribution.** Europe, European parts of Russia, Turkey, and Siberia.

**Remarks.**Lucas et al. (1995) synonymised *C. nasutula* under *C. vicina*.

***Chrysogaster cemiteriorum* (Linnaeus, 1758)**

**Reference.**Peck (1988) as *Chrysogaster chalybeata* Meigen, 1822; Gudjabidze (2002) as *C. chalybeata*.

**Distribution.** Palaearctic.

***Chrysogaster musatovi* Stackelberg, 1952**

**Reference.**Levitin (1962); Peck (1988); Gudjabidze (2002); Barkalov and Mutin (2018); Speight (2018a).

**New records.** GEORGIA • 4♀; L53, 1 Aug 2001, J.-H. Stuke leg.; ZFMK-DIP-00057932, ZFMK-DIP-00057933, ZFMK-DIP-00057934, ZFMK-DIP-00057935.

**Distribution.** Ukraine, Transcaucasia, Kazakhstan, Kyrgyzstan, and Tajikistan.

**Remarks.** The taxonomic status of *C. musatovi* is unclear. Maibach et al. (1994a) and Speight (2018a) suggested the possibility that *C. musatovi* and *Chrysogaster basalis* Loew, 1857 could be the same species, and Thompson (2019) listed *C. musatovi* as synonym of *C. basalis*. More taxonomic work and the study of the type material are needed to solve the taxonomic status of *C. musatovi*. The four females here reported key out to *C. musatovi* using the identification key by Stackleberg (1989).

***Chrysogaster solstitialis* (Fallén, 1817)**

**Reference.**Levitin (1962); Peck (1988); Gudjabidze (2002); Speight (2018a).

**New records.** GEORGIA • 1♂; L21, 2 Jul 2018, S. Bot leg.; • 1♀; L33, 23 Jul 2018, X. Mengual leg.; ZFMK-DIP-00054186 = ZFMK-TIS-8000981.

**Genetics.** We obtained one DNA barcode for this taxon (MN621918). The BIN for this specimen is BOLD:AAJ4882. The nearest neighbour in BOLD systems (5.31% p-dist) is another BIN (BOLD:AAY8878) identified also as *C. solstitialis* with specimens from Morocco and Spain.

**Distribution.** Western Palaearctic, European parts of Russia, and Transcaucasia.

***Chrysotoxum arcuatum* (Linnaeus, 1758)**

**Reference.**Peck (1988); Gudjabidze (2002).

**New records.** GEORGIA • 1♀; L3, 17 Jun 2018, S. Bot leg.; • 1♀; L15, 26 Jun 2018, S. Bot leg.; • 1♂; L16, 27 Jun 2018, S. Bot leg.; • 1♀; L19, 29 Jun 2018, S. Bot leg.; • 1♀; L20, 30 Jun 2018, S. Bot leg.; • 1♀; L20, 1 Jul 2018, S. Bot leg.; • 1♂; L21, 3 Jul 2018, S. Bot leg.; • 1♀; L37, 25 Jul 2018, X. Mengual leg.; ZFMK-DIP-00053998 = ZFMK-TIS-8003420; • 2♀; L57, 3 Aug 2001, J.-H. Stuke leg.; ZFMK-DIP-00058245, ZFMK-DIP-00058246.

**Genetics.** We sequenced one specimen (MN621919) and its COI barcode has 99.18% similarity with a private sequence identified as *Chrysotoxum intermedium* Meigen, 1822.

**Distribution.** Palaearctic.

***Chrysotoxum bicinctum* (Linnaeus, 1758)**

**Reference.**Levitin (1962); Peck (1988); Gudjabidze (2002).

**New records.** GEORGIA • 1♂; L4, 18 Jun 2018, S. Bot leg.; • 1♂; L15, 26 Jun 2018, S. Bot leg.; • 1♂; L18, 28 Jun 2018, S. Bot leg.; • 2♂; L19, 29 Jun 2018, S. Bot leg.; • 3♂; L20, 30 Jun 2018, S. Bot leg.; • 1♂; L20, 1 Jul 2018, S. Bot leg.; • *10; L20, 1 Jul 2018, S. Bot obs.; • *20; L21, 2 Jul 2018, S. Bot obs.; • 1♀; L35, 24 Jul 2018, X. Mengual leg.; ZFMK-DIP-00054015 = ZFMK-TIS-8000951; • 2♀; L37, 25 Jul 2018, X. Mengual leg.; ZFMK-DIP-00053636 = ZFMK-TIS-8005536, ZFMK-DIP-00053637 = ZFMK-TIS-8005544; • 1♀; L39, 23–26 Jul 2018, malaise trap, X. Mengual, M. Espeland, B. Thormann leg.; ZFMK-DIP-00054016; • 1♂; L49, 4 Aug 2001, J.-H. Stuke leg.; ZFMK-DIP-00057479; • 1♀; L50, 4 Aug 2001, J.-H. Stuke leg.; ZFMK-DIP-00057480.

**Genetics.** Two specimens were sequenced (MN621920, MN621921) and their COI sequences showed an uncorrected pairwise distance of 0.152%, very similar to other published sequences of this species (> 99%). The BIN for these specimens is BOLD:AAJ0967.

**Distribution.** Western and Central Palaearctic into central Siberia.

***Chrysotoxum caucasicum* Sack, 1930**

**Reference.**Becker (1921) as *Chrysotoxum derivatum* Becker, 1921; Peck (1988); Gudjabidze (2002) as *Chrysotoxum caucasicum* Linnaeus, 1758 [sic]; Barkalov and Mutin (2018).

**Distribution.** Ukraine, Transcaucasia, Central Palaearctic, into Afghanistan.

***Chrysotoxum cautum* (Harris, 1778)**

**Reference.**Levitin (1962); Peck (1988); Gudjabidze (2002); Barkalov and Mutin (2018).

**New records.** GEORGIA • 1♀; L21, 3 Jul 2018, S. Bot leg.; • 1♀; L72, 29 Jun–13 Jul 2018, malaise trap, GGBC-members leg.; ZFMK-TIS-8002767.

**Genetics.** One specimen was sequenced (MN621922; BIN = BOLD:AAJ0972), with identical COI sequence to specimens of other countries. The nearest neighbour in BOLD systems is *Chrysotoxum tuberculatum* Shannon, 1926 (BOLD:ACH8118), a species known from the Far East Region and Sichuan province (China).

**Distribution.** Europe, Turkey, European parts of Russia, and into Altai Mountains.

**Remarks.** The year of publication for this species was a convention. Peck (1988) used the conventional dates based on Lisney (1960): 1776 for decad 1, 1776? for decad 2, and 1780? for decads 3, 4, and 5. Evenhuis (1997: page 342) found that the decad 2, where *Musca cautus* is described on page 60, was dated as 1778 in the “*Discours préliminaires*” to the *Encyclopédie méthodique par ordre des matières* – *Insectes*. Thus, the year of publication should be 1778.

***Chrysotoxum cisalpinum* Rondani, 1926.**

**Reference.**Peck (1988); Sommaggio 2007.

**Distribution.** France, Mediterranean Basin, Balkan Peninsula, Transcaucasia, eastwards into Tajikistan and Uzbekistan.

***Chrysotoxum elegans* Loew, 1841**

**Reference.**Tóth (1986); Peck (1988); Gudjabidze (2002) as *Chrysotoxum elegans* Loew, 1848 [sic]; Barkalov and Mutin (2018); Speight (2018a).

**New records.** GEORGIA • 1♂; L55, 31 Jul 2001, J.-H. Stuke leg.; ZFMK-DIP-00057481.

**Distribution.** Western Palaearctic, including Transcaucasia and Turkey.

***Chrysotoxum fasciolatum* (De Geer, 1776)**

**Reference.**Peck (1988); Gudjabidze (2002) as *Chrysotoxum fasciolatum* De egger, 1776 [sic]; Speight (2018a).

**New records.** GEORGIA • 1♂; L20, 30 Jun 2018, S. Bot leg.; • 2♀; L20, 1 Jul 2018, S. Bot leg.; • 1♂ 1♀; L20, 2 Jul 2018, S. Bot leg.

**Distribution.** Palaearctic, but not present in southern Europe.

***Chrysotoxum festivum* (Linnaeus, 1758)**

**Reference.**Levitin (1962); Peck (1988); Gudjabidze (2002).

**New records.** GEORGIA • 2♂; L19, 29 Jun 2018, S. Bot leg.; • 3♂; L20, 30 Jun 2018, S. Bot leg.; • 1♂ 1♀; L20, 1 Jul 2018, S. Bot leg.; • 1♂; L20, 2 Jul 2018, S. Bot leg.; • 2♀; L57, 2 Aug 2001, J.-H. Stuke leg.; ZFMK-DIP-00057483, ZFMK-DIP-00057484; • 1♂ 4♀; L57, 3 Aug 2001, J.-H. Stuke leg.; ZFMK-DIP-00057487, ZFMK-DIP-00057482, ZFMK-DIP-00057485, ZFMK-DIP-00057488, ZFMK-DIP-00057489.

**Distribution.** Palaearctic and northern India.

***Chrysotoxum intermedium* Meigen, 1822**

**Reference.**Peck (1988).

**Distribution.** Europe.

**Remarks.** The material from the Caucasus Region referred as *C. intermedium* needs re-examination to reassess its taxonomic identity as *C. lessonae* is reported here and the two species are very similar (see Speight 2018a).

***Chrysotoxum lessonae* Giglio-Tos, 1890**

**New records.** GEORGIA • 1♂; L37, 25 Jul 2018, X. Mengual leg.; ZFMK-DIP-00053638 = ZFMK-TIS-8005537; • 2♀; L38, 25 Jul 2018, X. Mengual leg.; ZFMK-DIP-00053639, ZFMK-DIP-00053640 = ZFMK-TIS-8005545; • 1♂; L71, 30 Jun–14 Jul 2018, malaise trap, GGBC-members leg.; ZFMK-TIS-8002732.

**Genetics.** We sequenced three specimens (MN621923, MN621924, MN621925); all with identical COI barcode. This species is not present in BOLD and we are providing the first COI sequences. The obtained sequences have a high similarity with sequences of *Chrysotoxum intermedium* (99.33%; BOLD:AAE9233).

**Distribution.** Europe, Turkey and Iran (Kazerani et al. 2017; Vujić et al. 2017).

**Remarks.** Reported for Georgia for the first time.

***Chrysotoxum octomaculatum* Curtis, 1837**

**Reference.**Levitin (1962); Peck (1988); Gudjabidze (2002) as *Chrysotoxum octomacullatum* Curtis [sic]; Barkalov and Mutin (2018).

**Distribution.** Western and Central Palaearctic.

***Chrysotoxum orthostylum* Vujić in Nedeljković et al., 2015**

**New records.** GEORGIA • 1♂; L10, 22 Jun 2018, S. Bot leg.; • 1♀; L12, 24 Jun 2018, S. Bot leg.

**Distribution.** Balkan Peninsula, Turkey, and Kyrgyzstan.

**Remarks.** Reported for Georgia for the first time.

***Chrysotoxum parmense* Rondani, 1845**

**Reference.**Peck (1988); Speight (2018a).

**Distribution.** Mediterranean Basin, Iran, Transcaucasia, and Central Palaearctic.

***Chrysotoxum parvulum* Violovitsh, 1973**

**Reference.**Violovitsh (1973); Peck (1988); Barkalov and Mutin (2018).

**New records.** GEORGIA • 1♀; L57, 3 Aug 2001, J.-H. Stuke leg.; ZFMK-DIP-00057510; • 2♂; L10, 22 Jun 2018, S. Bot leg.; • 1♀; L12, 24 Jun 2018, S. Bot leg.; • 1♂; L15, 26 Jun 2018, S. Bot leg.; • 1♂; L16, 27 Jun 2018, S. Bot leg.; • 1♂ 1♀; L19, 29 Jun 2018, S. Bot leg.; • 6♂; L20, 30 Jun 2018, S. Bot leg.; • 2♂ 1♀; L20, 1 Jul 2018, S. Bot leg.; • 1♂; L21, 2 Jul 2018, S. Bot leg.

**Distribution.** Transcaucasia.

***Chrysotoxum robustum* Portschinsky, 1887**

**Reference.**Peck (1988).

**Distribution.** Transcaucasia and Iran.

***Chrysotoxum vernale* Loew, 1841**

**Reference.**Tóth (1986); Peck (1988); Barkalov and Mutin (2018).

**New records.** GEORGIA • 5♂; L16, 27 Jun 2018, S. Bot leg.; • 2♂; L17, 28 Jun 2018, S. Bot leg.; • 1♂; L20, 30 Jun 2018, S. Bot leg.; • 2♂; L57, 3 Aug 2001, J.-H. Stuke leg.; ZFMK-DIP-00057508, ZFMK-DIP-00057509.

**Distribution.** Palaearctic.

***Chrysotoxum verralli* Collin, 1940**

**Reference.**Peck (1988); Gudjabidze (2002) as *Chrysotoxum verralli* Collin, 1931 [sic]; Speight (2018a).

**Distribution.** Europe, European parts of Russia, Transcaucasia, and into Siberia.

***Criorhina berberina* (Fabricius, 1805)**

**Reference.**Levitin (1962) as *Penthesilea berberina* F.; Tóth (1986) as *Brachymyia berberina* (Fabricius, 1805); Peck (1988) as *Brachymyia berberina*; Gudjabidze (2002) as *Criorrhina berberiana* (Fallén, 1817) [sic]; Barkalov and Mutin (2018).

**New records.** GEORGIA • 1♂; L16, 27 Jun 2018, S. Bot leg.; • 1♂; L20, 1 Jul 2018, S. Bot leg.; • 1♀; L72, 29 Jun–13 Jul 2018, malaise trap, GGBC-members leg.; ZFMK-TIS-8002769.

**Genetics.** We sequenced one specimen (MN621926), with BIN BOLD:AAZ5304 (BOLD:AAZ5304). The nearest neighbour in BOLD systems is a specimen of *Criorhina talyshensis* (Stackelberg, 1960) from Azerbaijan (2.6% p-dist).

**Distribution.** Europe, European parts of Russia, and Transcaucasia.

***Criorhina floccosa* (Meigen, 1822)**

**Reference.**Peck (1988) as *Brachymyia floccosa* (Meigen, 1822); Barkalov and Mutin (2018); Speight (2018a).

**New records.** GEORGIA • 1♀; L3, 17 Jun 2018, S. Bot leg.

**Distribution.** Europe, European parts of Russia, and Transcaucasia.

***Criorhina portschinskyi* (Stackelberg, 1955)**

**Reference.**Peck (1988); Gudjabidze (2002) as *Criorrhina portshinski* Stackelberg, 1956 [sic]; Barkalov and Mutin (2018).

**New records.** GEORGIA • 1♂; L16, 27 Jun 2018, S. Bot leg.

**Distribution.** Transcaucasia and Northern Caucasus.

***Criorhina ranunculi* (Panzer, 1804)**

**Reference.**Peck (1988).

**Distribution.** Europe, European parts of Russia, and Transcaucasia.

***Dasysyrphus albostriatus* (Fallén, 1817)**

**Reference.**Levitin (1962) as *Syrphus albostriatus* Mg. [sic]; Tóth (1986); Gudjabidze (2002) as *Syrphus albostriatus* (Fallén, 1817); Barkalov and Mutin (2018); Speight (2018a).

**New records.** GEORGIA • 1♀; L1, 16 Jun 2018, S. Bot leg.; • 1♀; L29, 19 Jul 2018, X. Mengual leg.; ZFMK-DIP-00053840 = ZFMK-TIS-8005584; • 1♀; L31, 20 Jul 2018, X. Mengual leg.; ZFMK-DIP-00054056 = ZFMK-TIS-8000873; • 1♀; L36, 24 Jul 2018, X. Mengual leg.; ZFMK-DIP-00054055 = ZFMK-TIS-8000998.

**Genetics.** We successfully sequenced one specimen (MN621927), with BIN BOLD:AAL1242. This BIN has an average variation of 0.17% (p-distance) within the BIN (0.48% max) and 2.41% (p-distance) with the nearest neighbour in BOLD systems, *Dasysyrphus eggeri* (Schiner, 1861) (BOLD:AAO9822).

**Distribution.** Palaearctic.

***Dasysyrphus eggeri* (Schiner, 1861)**

**Reference.**Peck (1988); Gudjabidze (2002) as *Syrphus eggeri* Schiner, 1860 [sic]; Barkalov and Mutin (2018); Speight (2018a).

**Distribution.** Palaearctic.

***Dasysyrphus friuliensis* (Van der Goot, 1960)**

**New records.** GEORGIA • 1♀; L20, 1 Jul 2018, S. Bot leg.

**Distribution.** Palaearctic.

**Remarks.** Reported for Georgia for the first time.

***Dasysyrphus pinastri* (De Geer, 1776)**

**Reference.**Barkalov and Mutin (2018) from Transcaucasia.

**New records.** GEORGIA • 1♂ 1♀; L18, 28 Jun 2018, S. Bot leg.

**Distribution.** Palaearctic.

**Remarks.** The name *pinastri* De Geer, 1776 here is applied *sensu*Locke and Skevington (2013), and it might refer to *Dasysyrphus lunulatus* (Meigen, 1822) of recent European authors (Doczkal 1996a; Speight 2018a).

***Dasysyrphus tricinctus* (Fallén, 1817)**

**Reference.**Tóth (1986); Peck (1988); Gudjabidze (2002) as *Syrphus tricinctus* (Fallén, 1817).

**New records.** GEORGIA • *1; L20, 30 Jun 2018, S. Bot obs.

**Distribution.** Palaearctic.

***Dasysyrphus venustus* (Meigen, 1822)**

**Reference.**Tóth (1986) as *Dasysyrphus lunulatus* (Meigen, 1822) and *D. venustus*; Peck (1988) as *D. lunulatus* and as *D. venustus*; Gudjabidze (2002) as *Syrphus lunutatus* Meigen, 1822 [sic] and *Syrphus venustus* Meigen, 1822.

**New records.** GEORGIA • 2♂ 2♀; L16, 27 Jun 2019, S. Bot leg.; • 1♂; L20, 1 Jul 2018, S. Bot leg.

**Distribution.** Holarctic.

**Remarks.**Locke and Skevington (2013) explained that the name *lunulatus* Meigen, 1822 has been used for two different taxa: *venustus* Meigen (of authors nec. Meigen) and *pinastri* De Geer (auctt.; Vockeroth 1986). In their work, Locke and Skevington (2013) stated that Vockeroth (1969) used the name *lunulatus* as *pinastri*; however, its correct usage should be as a synonym of *venustus* (Vockeroth 1986). Thus, we follow Locke and Skevington (2013) and consider all the previous citations of *Dasysyrphus lunulatus* as synonyms of *Dasysyrphus venustus*.

***Didea fasciata* Macquart, 1834**

**Reference.**Levitin (1962) as *Didea fasciata* Mg. [sic]; Tóth (1986); Peck (1988); Gudjabidze (2002).

**New records.** GEORGIA • 2♂; L1, 16 Jun 2018, S. Bot leg.; • 1♂; L3, 17 Jun 2018, S. Bot leg.; • 2♂ 3♀; L24, 17 Jul 2018, X. Mengual leg.; ZFMK-DIP-00053736, ZFMK-DIP-00053737, ZFMK-DIP-00053739, ZFMK-DIP-00053740, ZFMK-DIP-00054163 = ZFMK-TIS-8000954; • 1♂; L25, 18 Jul 2018, X. Mengual leg.; ZFMK-DIP-00053738 = ZFMK-TIS-8005586; • 1♀; L37, 25 Jul 2018, X. Mengual leg.; ZFMK-DIP-00053741 = ZFMK-TIS-8005592; • 1♂; L37, 25 Jul 2018, X. Mengual leg.; ZFMK-DIP-00054162.

**Genetics.** The two sequenced specimens (MN621928, MN621929) differ only 1.67% (p-dist). The BIN for these specimens is BOLD:AAI9912, with an average distance of 0.54% (p-distance) within BIN (2.6% max) and 5.3% (p-distance) with the nearest neighbour in BOLD systems, *Didea intermedia* Loew, 1846 (BOLD:ABW1162).

**Distribution.** Holarctic and Indomalayan Region (northern India and Taiwan).

***Didea intermedia* Loew, 1846**

**Reference.**Peck (1988); Gudjabidze (2002).

**New records.** GEORGIA • 1♂; L4, 18 Jun 2018, S. Bot leg.; • 1♂; L70, 30 Jun–14 Jul 2018, malaise trap, GGBC-members leg.; ZFMK-TIS-8002702.

**Genetics.** A single male specimen was sequenced (MN621930), BIN BOLD:ABW1162. This specimen differs 5.93–6.08% (uncorrected pair-wise distance) from the previous specimens of *D. fasciata*.

**Distribution.** Palaearctic.

**Remarks.**Stackelberg and Richter (1968) stated that Levitin (1962) recorded this species from Borjomi area. In the original publication, Levitin (1962) did not list this species.

***Doros profuges* (Harris, 1779)**

**Reference.**Peck (1988) as *Doros conopseus* (Fabricius, 1775).

**Distribution.** Palaearctic.

**Remarks.**Thompson et al. (1982) explained the application of the name *Doros profuges* to this taxon.

The year of publication for this species was a convention. The original work by Harris (1776–1780) was published in five ‘decads’ or parts. Peck (1988) used the conventional date of 1780? with a question mark for decads 3, 4, and 5 based on Lisney (1960). Evenhuis (1997: page 343) established that the decad 3, where *Musca profuges* is described on page 81, was dated as 1779 based on the latest date of the plates. Thus, the year of publication should be 1779.

***Epistrophe diaphana* (Zetterstedt, 1843)**

**Reference.**Peck (1988); Barkalov and Mutin (2018).

**New records.** GEORGIA • 1♀; L21, 3 Jul 2018, S. Bot leg.

**Distribution.** Palaearctic.

***Epistrophe eligans* (Harris, 1779)**

**Reference.**Tóth (1986); Peck (1988); Gudjabidze (2002) as *Syrphus bifasciatus* Fallén, 1817 [sic] (= *Syrphus bifasciata* Fabricius, 1794); Speight (2018a).

**Distribution.** Europe, European parts of Russia, Transcaucasia, and Turkey.

**Remarks.** The year of publication for this species is a convention. The original work by Harris (1776–1780) was published in five ‘decads’ or parts. Peck (1988) used the conventional date of 1780? with a question mark for decads 3, 4, and 5 based on Lisney (1960). Evenhuis (1997: page 343) established that the decad 4, where *Musca eligans* is described on page 105, was dated as 1779 based on the latest date of the plates. Thus, the year of publication should be 1779.

***Epistrophe flava* Doczkal & Schmid, 1994**

**New records.** GEORGIA • 1♂; L53, 1 Aug 2001, J.-H. Stuke leg.; ZFMK-DIP-00057585; • 1♂; L57, 3 Aug 2001, J.-H. Stuke leg.; ZFMK-DIP-00057586.

**Remarks.** Reported for Georgia for the first time.

**Distribution.** Palaearctic.

***Epistrophe grossulariae* (Meigen, 1822)**

**Reference.**Levitin (1962) as *Syrphus grossulariae* Mg.; Tóth (1986); Peck (1988); Gudjabidze (2002) as *Syrphus grossulariae* .

**New records.** GEORGIA • 3♂ 1♀; L21, 3 Jul 2018, S. Bot leg.; • 1♂ 1♀; L36, 24 Jul 2018, X. Mengual leg.; ZFMK-DIP-00053854 = ZFMK-TIS-8005587, ZFMK-DIP-00053995 = ZFMK-TIS-8003453; • 4♂ 1♀; L57, 3 Aug 2001, J.-H. Stuke leg.; ZFMK-DIP-00057587, ZFMK-DIP-00057588, ZFMK-DIP-00057589, ZFMK-DIP-00057590, ZFMK-DIP-00057591.

**Genetics.** One specimen was sequenced (MN621931) with BIN BOLD:AAI5313. The obtained COI sequence is very similar (> 99.6%) to other published sequences of this species from Europe. The nearest neighbour in BOLD systems (1.76% p-dist) is another BIN of *E. grossulariae* with specimens only from Canada (BOLD:ABY7460).

**Distribution.** Holarctic.

***Epistrophe leiophthalma* (Schiner & Egger, 1853)**

**Reference.**Violovitsh (1979) as *Stackelbergina amicorum* Violovitsh, 1979; Peck (1988); Barkalov and Mutin (2018); Speight (2018a).

**Distribution.** Europe and Transcaucasia.

***Epistrophe nitidicollis* (Megerle in Meigen, 1822)**

**Reference.**Gudjabidze (2002) as *Syrphus nitidicollis* Meigen, 1822 [sic].

**New records.** GEORGIA • 1♂; L2, 16 Jun 2018, S. Bot leg.

**Distribution.** The geographic range of this species needs reassessment due to the confusion with related species *Epistrophe melanostoma* (Zetterstedt, 1943) and *Epistrophe ochrostoma* (Zetterstedt, 1849) until recently (Doczkal and Schmid 1994).

***Epistrophe ochrostoma* (Zetterstedt, 1849)**

**Reference.**Peck (1988).

**Distribution.** The geographic range of this species needs reassessment due to the confusion with related species *Epistrophe melanostoma* (Zetterstedt, 1943) and *Epistrophe nitidicollis* until recently (Doczkal and Schmid 1994).

***Epistrophella euchroma* (Kowarz, 1885)**

**Reference.**Peck (1988) as Epistrophe (Epistrophella) euchroma (Kowarz, 1885); Speight (2018a) as *Meligramma euchroma* (Kowarz, 1885).

**Distribution.** Europe, European parts of Russia, Transcaucasia, and into Siberia.

***Episyrphus balteatus* (De Geer, 1776)**

**Reference.**Levitin (1962) as *Syrphus balteatus* Deg.; Tóth (1986); Gudjabidze (2002) as *Syrphus balteatus* (De Geer, 1776); Barjadze and Gratiashvili (2010).

**New records.** GEORGIA • 1♂; L1, 15 Jun 2018, S. Bot leg.; • 1♂ 2♀; L1, 16 Jun 2018, S. Bot leg.; • *5; L1, 16 Jun 2018, S. Bot obs.; • 1♀; L7, 19 Jun 2018, S. Bot leg.; • 1♂; L10, 22 Jun 2018, S. Bot leg.; • *4; L10, 22 Jun 2018, S. Bot obs.; • *5; L19, 29 Jun 2018, S. Bot obs.; • *4; L21, 1 Jul 2018, S. Bot obs.; • 1♀; L21, 2 Jul 2018, S. Bot leg.; • *1; L22, 3 Jul 2018, S. Bot obs.; • 3♂ 2♀; L24, 17 Jul 2018, X. Mengual leg.; ZFMK-DIP-00053641, ZFMK-DIP-00054081 = ZFMK-TIS-8000959, ZFMK-DIP-00054084 = ZFMK-TIS-8000967, ZFMK-DIP-00053642, ZFMK-DIP-00053643; • 3♀; L25, 18 Jul 2018, X. Mengual leg.; ZFMK-DIP-00053644 = ZFMK-TIS-8005565, ZFMK-DIP-00053645, ZFMK-DIP-00053646; • 1♀; L28, 19 Jul 2018, X. Mengual leg.; ZFMK-DIP-00053647; • 1♀; L29, 19 Jul 2018, X. Mengual leg.; ZFMK-DIP-00053648; • 1♀; L31, 20 Jul 2018, X. Mengual leg.; ZFMK-DIP-00053649; • 1♀; L31, 20 Jul 2018, X. Mengual leg.; ZFMK-DIP-00054083; • 1♀; L32, 22 Jul 2018, X. Mengual leg.; ZFMK-DIP-00053650; • 1♂; L35, 24 Jul 2018, X. Mengual leg.; ZFMK-DIP-00053651; • 1♀; L36, 24 Jul 2018, X. Mengual leg.; ZFMK-DIP-00054082 = ZFMK-TIS-8000997; • 1♀; L37, 25 Jul 2018, X. Mengual leg.; ZFMK-DIP-00053652; • 1♂; L38, 25 Jul 2018, X. Mengual leg.; ZFMK-DIP-00053653 = ZFMK-TIS-8005573; • 1♀; L45, 25 Jul 2001, J.-H. Stuke leg.; ZFMK-DIP-00057473; • 1♀; L49, 4 Aug 2001, J.-H. Stuke leg.; ZFMK-DIP-00057475; • 1♂; L51, 24 Jul 2001, J.-H. Stuke leg.; ZFMK-DIP-00057471; • 2♀; L57, 3 Aug 2001, J.-H. Stuke leg.; ZFMK-DIP-00057477, ZFMK-DIP-00057478; • 1♂; L58, 27 Jul 2001, J.-H. Stuke leg.; ZFMK-DIP-00057472, ZFMK-DIP-00057476; • 1♀; L31, 23 Jul 2018, A. Reimann leg.; MTD-Dip-A-R-4527; • 1♂ 5♀; L70, 30 Jun–14 Jul 2018, malaise trap, GGBC-members leg.; ZFMK-TIS-8002703, ZFMK-TIS-8002704, ZFMK-TIS-8002705, ZFMK-DIP-00061260, ZFMK-DIP-00061262, ZFMK-DIP-00061263; • 5♀; L70, 30 Jun–14 Jul 2018, malaise trap, GGBC-members leg.; MTD-Dip-A-R-4579; • 2♂ 9♀; L71, 30 Jun–14 Jul 2018, malaise trap, GGBC-members leg.; ZFMK-TIS-8002728, ZFMK-TIS-8002729, ZFMK-TIS-8002730, ZFMK-DIP-00061264, ZFMK-DIP-00061265, ZFMK-DIP-00061266, ZFMK-DIP-00061267, ZFMK-DIP-00061268, ZFMK-DIP-00061269, ZFMK-DIP-00061270, ZFMK-DIP-00061271; • 4♂ 3♀; L72, 29 Jun–13 Jul 2018, malaise trap, GGBC-members leg.; MTD-Dip-A-R-4554, ZFMK-TIS-8002764, ZFMK-TIS-8002765, ZFMK-DIP-00061272, ZFMK-DIP-00061273, ZFMK-DIP-00061274, ZFMK-DIP-00061275.

**Genetics.** Eight specimens were sequenced (MN621932, MN621933, MN621934, MN621935, MN621936, MN621937, MN621938, MN621939) and their COI barcodes showed little variation (0–0.27%). The BIN is BOLD:AAC6833, but this BIN has several species besides *E. balteatus*; in other words, the p-dist among different taxa is smaller than among specimens of *E. balteatus*.

**Distribution.** Palaearctic and Indomalayan Region. The records from the Indomalayan Region need confirmation due to the confusion with other morphologically similar *Episyrphus* species. Speight (2018a) lists this species from Australia, but Wright and Skevington (2013) do not report it in their revision of the Australian species of *Episyrphus*.

***Eriozona syrphoides* (Fallén, 1817)**

**Reference.**Tóth (1986); Peck (1988); Gudjabidze (2002).

**New records.** GEORGIA • 1♀; L20, 30 Jun 2018, S. Bot leg.

**Distribution.** Palaearctic.

***Eristalinus aeneus* (Scopoli, 1763)**

**Reference.**Peck (1988); Gudjabidze (2002) as *Lathyrophthalmus aenus* (Scopoli, 1763).

**New records.** GEORGIA • 1♀; L60, 26 Jul 2001, J.-H. Stuke leg.; ZFMK-DIP-00058022.

**Distribution.** Holarctic, Afrotropical region, Indomalayan Region, Hawaii, and Australasian Region.

***Eristalinus megacephalus* (Rossi, 1794)**

**Reference.**Levitin (1962) as *Lathyrophthalmus quinquelineatus* F.; Peck (1988) as *Eristalinus quinquelineatus* (Fabricius, 1781); Gudjabidze (2002) as *Lathyrophthalmus quinqueliniatus* Fabricius, 1805 [sic]; Khachidze (2013) as field observation. Dirickx (1998) pointed out that European records of *E. quinquelineatus* are erroneous and refer to *E. megacephalus*.

**New records.** GEORGIA • 1♀; L7, 19 Jun 2018, S. Bot leg.; • 1♂; L11, 23 Jun 2018, S. Bot leg.; • 4♂ 4♀; L59, 28 Jul 2001, J.-H. Stuke leg.; ZFMK-DIP-00058004, ZFMK-DIP-00058005, ZFMK-DIP-00058006, ZFMK-DIP-00058007, ZFMK-DIP-00058008, ZFMK-DIP-00058009, ZFMK-DIP-00058010, ZFMK-DIP-00058011; • 3♂ 5♀; L60, 26 Jul 2001, J.-H. Stuke leg.; ZFMK-DIP-00058017, ZFMK-DIP-00058018, ZFMK-DIP-00058019, ZFMK-DIP-00058012, ZFMK-DIP-00058013, ZFMK-DIP-00058014, ZFMK-DIP-00058015, ZFMK-DIP-00058016; • 2♂; L67, 29 Jul 2001, J.-H. Stuke leg.; ZFMK-DIP-00058020, ZFMK-DIP-00058021.

**Distribution.** Mediterranean Basin, Turkey, Transcaucasia, and the Afrotropical Region.

***Eristalinus sepulchralis* (Linnaeus, 1758)**

**Reference.**Tóth (1986); Peck (1988); Gudjabidze (2002) as *Eristalis sepulchralis* Linnaeus, 1758 [sic] and *Eristalinus sepulclaris* (Linnaeus, 1758) [sic].

**New records.** GEORGIA • 1♂; L53, 1 Aug 2001, J.-H. Stuke leg.; ZFMK-DIP-00058046; • 1♂ 4♀; L60, 26 Jul 2001, J.-H. Stuke leg.; ZFMK-DIP-00058044, ZFMK-DIP-00058039, ZFMK-DIP-00058040, ZFMK-DIP-00058041, ZFMK-DIP-00058042; • 1♂ 2♀; L67, 29 Jul 2001, J.-H. Stuke leg.; ZFMK-DIP-00058045, ZFMK-DIP-00058038, ZFMK-DIP-00058043.

**Distribution.** Palaearctic and India.

***Eristalinus taeniops* (Wiedemann, 1818)**

**Reference.**Peck (1988); Speight (2018a).

**New records.** GEORGIA • 1♂; L42, 25 Jul 2018, A. Reimann leg.; MTD-Dip-A-R-4537.

**Distribution.** Palaearctic, Indomalayan Region, Afrotropical Region, and South America (introduced).

**Eristalis (Eoseristalis) alpina (Panzer, 1798)**

**Reference.**Levitin (1962) as *Eristalis alpinus* Pz.; Peck (1988); Speight (2018a).

**Distribution.** Palaearctic.

**Remarks.**Portschinsky (1892) described Eristalis alpinus var. caucasicus Portschinsky, 1892 from the valley of the river Akstafa (also known as Aghstev) in Armenia and Azerbaijan. The taxonomic status of *caucasica* needs re-examination.

**Eristalis (Eoseristalis) arbustorum (Linnaeus, 1758)**

**Reference.**Radde (1899); Levitin (1962); Tóth (1986) as *Eoseristalis arbustorum* (Linnaeus, 1758); Peck (1988); Gudjabidze (2002).

**New records.** GEORGIA • 1♂ 1♀; L10, 22 Jun 2018, S. Bot leg.; • 2♀; L11, 23 Jun 2018, S. Bot leg.; • 1♂ 1♀; L11, 29 Jun 2018, S. Bot leg.; • 1♀; L15, 26 Jun 2018, S. Bot leg.; • 1♂ 1♀; L24, 17 Jul 2018, X. Mengual leg.; ZFMK-DIP-00053712 = ZFMK-TIS-8005552, ZFMK-DIP-00053720; • 1♂; L26, 18 Jul 2018, X. Mengual leg.; ZFMK-DIP-00054178; • 3♂ 3♀; L28, 19 Jul 2018, X. Mengual leg.; ZFMK-DIP-00053713, ZFMK-DIP-00053714, ZFMK-DIP-00053715, ZFMK-DIP-00053721, ZFMK-DIP-00053722, ZFMK-DIP-00054180 = ZFMK-TIS-8000975; • 1♀; L29, 19 Jul 2018, X. Mengual leg.; ZFMK-DIP-00053716; • 1♂ 6♀; L31, 20 Jul 2018, X. Mengual leg.; ZFMK-DIP-00054183, ZFMK-DIP-00053718 = ZFMK-TIS-8005560, ZFMK-DIP-00053719, ZFMK-DIP-00054177, ZFMK-DIP-00054182, ZFMK-DIP-00054184, ZFMK-DIP-00054185; • 1♂; L31, 23 Jul 2018, A. Reimann leg.; MTD-Dip-A-R-4524; • 3♂ 1♀; L33, 22 Jul 2018, X. Mengual leg.; ZFMK-DIP-00053709, ZFMK-DIP-00053710, ZFMK-DIP-00053711, ZFMK-DIP-00053723; • 1♀; L34, 22 Jul 2018, X. Mengual leg.; ZFMK-DIP-00053724; • 1♂ 1♀; L35, 24 Jul 2018, X. Mengual leg.; ZFMK-DIP-00053707, ZFMK-DIP-00053717; • 2♀; L37, 25 Jul 2018, B. Thormann leg.; ZFMK-DIP-00054179 = ZFMK-TIS-8004100, ZFMK-DIP-00054181 = ZFMK-TIS-8004106; • 2♂; L37, 25 Jul 2018, X. Mengual leg.; ZFMK-DIP-00053708, ZFMK-DIP-00053706; • 1♀; L42, 25 Jul 2018, A. Reimann leg.; MTD-Dip-A-R-4542; • 1♂ 1♀; L46, 24 Jul 2001, J.-H. Stuke leg.; ZFMK-DIP-00058037, ZFMK-DIP-00058027; • 1♂; L49, 4 Aug 2001, J.-H. Stuke leg.; ZFMK-DIP-00058035, ZFMK-DIP-00058036; • 3♂ 1♀; L53, 1 Aug 2001, J.-H. Stuke leg.; ZFMK-DIP-00058032, ZFMK-DIP-00058033, ZFMK-DIP-00058034, ZFMK-DIP-00058024; • 2♀; L57, 3 Aug 2001, J.-H. Stuke leg.; ZFMK-DIP-00058025, ZFMK-DIP-00058026; • 3♂ 1♀; L59, 28 Jul 2001, J.-H. Stuke leg.; ZFMK-DIP-00058029, ZFMK-DIP-00058030, ZFMK-DIP-00058031, ZFMK-DIP-00058028; • 2♂; L69, 18 Jul 2018, A. Reimann leg.; MTD-Dip-A-R-4498, ZFMK-TIS-8002662; • 1♀; L70, 30 Jun–14 Jul 2018, malaise trap, GGBC-members leg.; ZFMK-TIS-8002683.

**Genetics.** We sequenced six specimens (MN621940, MN621941, MN621942, MN621943, MN621944, MN621945) with BIN BOLD:ADK2468. The uncorrected pairwise distance among them was very low (0–0.16%). This BIN also has some specimens of the Nearctic species *Eristalis brousii* Williston, 1882. The nearest neighbour in BOLD systems is the Palaearctic species *Eristalis abusiva* Collin, 1931 (BOLD:ADK2468, 1.97% p-dist).

**Distribution.** Holarctic and northern India.

**Eristalis (Eoseristalis) horticola (De Geer, 1776)**

**Reference.**Peck (1988); Gudjabidze (2002).

**Distribution.** Palaearctic and India.

**Eristalis (Eoseristalis) intricaria (Linnaeus, 1758)**

**Reference.**Peck (1988); Gudjabidze (2002) as *Eristalis intrikarius* Linnaeus, 1758 [sic].

**Distribution.** Europe, European parts of Russia, Transcaucasia, into Siberia.

**Eristalis (Eoseristalis) jugorum Egger, 1858**

**Reference.**Peck (1988); Gudjabidze (2002); Speight (2018a).

**Distribution.** Europe, European parts of Russia, Transcaucasia, Turkey, and Iran.

**Eristalis (Eoseristalis) nemorum (Linnaeus, 1758)**

**Reference.**Levitin (1962); Peck (1988); Gudjabidze (2002).

**Distribution.** Holarctic.

**Eristalis (Eoseristalis) pertinax (Scopoli, 1763)**

**Reference.**Levitin (1962); Peck (1988); Gudjabidze (2002) as *Eristalis pertinax* Scopoli, 1763 [sic].

**New records.** GEORGIA • 1♂; L3, 17 Jun 2018, S. Bot leg.; • 1♂; L11, 23 Jun 2018, S. Bot leg.; • 1♀; L19, 29 Jun 2018, S. Bot leg.; • 1♂; L20, 30 Jun 2018, S. Bot leg.; • 1♂; L21, 2 Jul 2018, S. Bot leg.; • 6♂ 1♀; L28, 19 Jul 2018, X. Mengual leg.; ZFMK-DIP-00053728, ZFMK-DIP-00053729, ZFMK-DIP-00053730, ZFMK-DIP-00053731, ZFMK-DIP-00053732, ZFMK-DIP-00054176 = ZFMK-TIS-8000973, ZFMK-DIP-00053727; • 1♂; L29, 19 Jul 2018, X. Mengual leg.; ZFMK-DIP-00053733 = ZFMK-TIS-8005553; • 1♂; L30, 19 Jul 2018, X. Mengual leg.; ZFMK-DIP-00053725; • 2♂; L37, 25 Jul 2018, X. Mengual leg.; ZFMK-DIP-00053726 = ZFMK-TIS-8005561, ZFMK-DIP-00053992 = ZFMK-TIS-8003422; • 2♂; L42, 25 Jul 2018, A. Reimann leg.; MTD-Dip-A-R-4534; • 1♀; L42, 25 Jul 2018, A. Reimann leg.; MTD-Dip-A-R-4540; • 1♂; L57, 3 Aug 2001, J.-H. Stuke leg.; ZFMK-DIP-00057986.

**Genetics.** One specimen was sequenced (MN621946), with BIN BOLD:AAQ3585 (average p-dist 0.17%; max p-dist 1.41%). The nearest neighbour in BOLD systems is *Eristalis obscura* Loew, 1866 (BOLD:AAA6459, 5.53% p-dist).

**Distribution.** Europe, European parts of Russia, Transcaucasia, and Turkey.

**Eristalis (Eoseristalis) rupium Fabricius, 1805**

**Reference.**Levitin (1962); Peck (1988); Gudjabidze (2002) as *E. rapium* Fabricius, 1777 [sic].

**Distribution.** Holarctic.

**Eristalis (Eoseristalis) similis (Fallén, 1817)**

**Reference.**Tóth (1986) as *Eoseristalis pratorum* Meigen, 1822; Peck (1988) as *Eristalis pratorum* Meigen, 1822; Gudjabidze (2002) as *Eristalis pratorum*.

**New records.** GEORGIA • 1♀; L28, 19 Jul 2018, X. Mengual leg.; ZFMK-DIP-00053735 = ZFMK-TIS-8005562; • 1♂; L29, 19 Jul 2018, X. Mengual leg.; ZFMK-DIP-00053734 = ZFMK-TIS-8005554; • 1♀; L33, 22 Jul 2018, X. Mengual leg.; ZFMK-DIP-00053993 = ZFMK-TIS-8003429; • 1♂; L42, 25 Jul 2018, A. Reimann leg.; MTD-Dip-A-R-4541; • 1♂; L70, 30 Jun–14 Jul 2018, malaise trap, GGBC-members leg.; ZFMK-TIS-8002684.

**Genetics.** Four specimens were successfully sequenced (MN621947, MN621948, MN621949, MN621950); BIN BOLD:AAY9892. The similarity among these sequences was very high (99.85–100%). The nearest neighbour in BOLD systems is *Eristalis obscura* Loew, 1866 (BOLD:AAA6459, 6.39% p-dist).

**Distribution.** Palaearctic.

**Remarks.**Nielsen (1995) synonymised *Eristalis pratorum* Megerle in Meigen, 1822 under *E. similis*.

**Eristalis (Eoseristalis) transcaucasica Kuznetzov, 1994**

**Reference.**Kuznetzov (1994).

**Distribution.** Northern Caucasus and Transcaucasia.

**Eristalis (Eristalis) tenax (Linnaeus, 1758)**

**Reference.**Radde (1899); Levitin (1962) as *Eristalomyia tenax* L.; Tóth (1986); Peck (1988); Gudjabidze (2002).

**New records.** GEORGIA • *1; L1, 16 Jun 2018, S. Bot obs.; • 1♂; L2, 16 Jun 2018, S. Bot leg.; • 2♂ 1♀; L3, 17 Jun 2018, S. Bot leg.; • *200; L4, 18 Jun 2018, S. Bot obs.; • *50; L8, 20 Jun 2018, S. Bot obs.; • 1♂; L10, 22 Jun 2018, S. Bot leg.; • *50; L10, 22 Jun 2018, S. Bot obs.; • *10; L11, 23 Jun 2018, S. Bot obs.; • 1♀; L11, 23 Jun 2018, S. Bot leg.; • 1♀; L11, 29 Jun 2018, S. Bot leg.; • *5; L12, 24 Jun 2018, S. Bot obs.; • *20; L19, 29 Jun 2018, S. Bot obs.; • *10; L21, 1 Jul 2018, S. Bot obs.; • *5; L22, 3 Jul 2018, S. Bot obs.; • 5♂ 3♀; L24, 17 Jul 2018, X. Mengual leg.; ZFMK-DIP-00053685, ZFMK-DIP-00053686, ZFMK-DIP-00053687, ZFMK-DIP-00053688, ZFMK-DIP-00054172 = ZFMK-TIS-8000971, ZFMK-DIP-00053684, ZFMK-DIP-00053689, ZFMK-DIP-00054165 = ZFMK-TIS-8000960; • 3♀; L25, 18 Jul 2018, X. Mengual leg.; ZFMK-DIP-00053690, ZFMK-DIP-00053981 = ZFMK-TIS-8003446, ZFMK-DIP-00053984 = ZFMK-TIS-8003445; • 3♀; L26, 18 Jul 2018, X. Mengual leg.; ZFMK-DIP-00053691 = ZFMK-TIS-8005551, ZFMK-DIP-00053986 = ZFMK-TIS-8003439, ZFMK-DIP-00053989 = ZFMK-TIS-8003438; • 5♂ 2♀; L28, 19 Jul 2018, X. Mengual leg.; ZFMK-DIP-00053692, ZFMK-DIP-00053693, ZFMK-DIP-00053694, ZFMK-DIP-00054164 = ZFMK-TIS-8000974, ZFMK-DIP-00054175 = ZFMK-TIS-8000980, ZFMK-DIP-00053697, ZFMK-DIP-00054174 = ZFMK-TIS-8000977; • 1♀; L30, 19 Jul 2018, X. Mengual leg.; ZFMK-DIP-00053696; • 2♂ 1♀; L31, 20 Jul 2018, X. Mengual leg.; ZFMK-DIP-00053698, ZFMK-DIP-00053985 = ZFMK-TIS-8003443, ZFMK-DIP-00053980 = ZFMK-TIS-8003441; • 1♂; L31, 23 Jul 2018, A. Reimann leg.; ZFMK-TIS-8002670; • 1♂ 3♀; L31, 23 Jul 2018, A. Reimann leg.; MTD-Dip-A-R-4508; • 3♂ 6♀; L33, 22 Jul 2018, X. Mengual leg.; ZFMK-DIP-00053699, ZFMK-DIP-00053982 = ZFMK-TIS-8003433, ZFMK-DIP-00053991 = ZFMK-TIS-8003432, ZFMK-DIP-00053695, ZFMK-DIP-00053700, ZFMK-DIP-00053983 = ZFMK-TIS-8003436, ZFMK-DIP-00053987 = ZFMK-TIS-8003428, ZFMK-DIP-00053988 = ZFMK-TIS-8003431, ZFMK-DIP-00053990 = ZFMK-TIS-8003430; • 1♀; L34, 22 Jul 2018, X. Mengual leg.; ZFMK-DIP-00053701; • 1♂; L35, 24 Jul 2018, X. Mengual leg.; ZFMK-DIP-00053702; • 1♀; L36, 24 Jul 2018, X. Mengual leg.; ZFMK-DIP-00053703; • 4♂; L37, 25 Jul 2018, B. Thormann leg.; ZFMK-DIP-00054168 = ZFMK-TIS-8004107, ZFMK-DIP-00054170 = ZFMK-TIS-8004105, ZFMK-DIP-00054171 = ZFMK-TIS-8004104, ZFMK-DIP-00054173 = ZFMK-TIS-8004096; • 1♂; L37, 25 Jul 2018, J. Thormann leg.; ZFMK-DIP-00054167 = ZFMK-TIS-8004080; • 1♀; L37, 25 Jul 2018, X. Mengual leg.; ZFMK-DIP-00053704; • 1♂ 1♀; L38, 25 Jul 2018, J. Thormann leg.; ZFMK-DIP-00054169 = ZFMK-TIS-8004029, ZFMK-DIP-00054166 = ZFMK-TIS-8004027; • 1♂; L38, 25 Jul 2018, X. Mengual leg.; ZFMK-DIP-00053705 = ZFMK-TIS-8005559; • 2♂ 1♀; L42, 25 Jul 2018, A. Reimann leg.; MTD-Dip-A-R-4539; • 1♂; L44, 18 Jul 2018, J. Astrin leg.; ZFMK-TIS-8000055; • 1♂; L46, 24 Jul 2001, J.-H. Stuke leg.; ZFMK-DIP-00057989; • 1♂; L51, 24 Jul 2001 J.-H. Stuke leg.; ZFMK-DIP-00057990; • 1♂; L53, 1 Aug 2001, J.-H. Stuke leg.; ZFMK-DIP-00057992; • 1♂; L57, 3 Aug 2001, J.-H. Stuke leg.; ZFMK-DIP-00057993; • 1♂; L58 27 Jul 2001, J.-H. Stuke leg.; ZFMK-DIP-00057991; • 2♂; L64 23 Jul 2001 J.-H. Stuke leg.; ZFMK-DIP-00057987, ZFMK-DIP-00057988; • 1♂ 2♀; L69, 18 Jul 2018, A. Reimann leg.; MTD-Dip-A-R-4496; • 1♀; L69, 18 Jul 2018, A. Reimann leg.; ZFMK-TIS-8002660; • 4♂; L70, 30 Jun–14 Jul 2018, malaise trap, GGBC-members leg.; ZFMK-TIS-8002679, ZFMK-TIS-8002680, ZFMK-DIP-00061276, ZFMK-DIP-00061277; • 3♂; L70, 30 Jun–14 Jul 2018, malaise trap, GGBC-members leg.; MTD-Dip-A-R-4571; • 4♀; L70, 30 Jun–14 Jul 2018, malaise trap, GGBC-members leg.; ZFMK-TIS-8002681, ZFMK-TIS-8002682, ZFMK-DIP-00061278, ZFMK-DIP-00061279; • 3♀; L70, 30 Jun–14 Jul 2018, malaise trap, GGBC-members leg.; MTD-Dip-A-R-4572; • 1♀; L71, 30 Jun–14 Jul 2018, malaise trap, GGBC-members leg.; ZFMK-TIS-8002733.

**Genetics.** Nine specimens were sequenced (MN621951, MN621952, MN621953, MN621954, MN621955, MN621956, MN621957, MN621958, MN621959), with BIN BOLD:AAB0391. The obtained sequences varied little (0–0.76%), and the nearest neighbour in BOLD systems is *Eristalis obscura* Loew, 1866 (4.19% p-dist).

**Distribution.** Almost cosmopolitan, known from all regions except the Antarctica.

***Eumerus amoenus* Loew, 1848**

**Reference.**Peck (1988); Speight (2018a).

**New records.** GEORGIA • 2♀; L39, 23–26 Jul 2018, malaise trap, X. Mengual, M. Espeland, B. Thormann leg.; ZFMK-DIP-00054120 = ZFMK-TIS-8000882; ZFMK-DIP-00054122 = ZFMK-TIS-8000883.

**Genetics.** We sequenced two specimens (MN621960, MN621961), which show an uncorrected pairwise distance of 0.46%. In BOLD systems there are two BINs with specimens identified as *E. amoenus*BOLD:ACO7316 and BOLD:AAY8911.

**Distribution.** Central and Southern Europe, Transcaucasia, Central Palaearctic to Mongolia.

***Eumerus argyropus* Loew, 1848**

**Reference.**Peck (1988); Gudjabidze (2002); Barkalov and Mutin (2018); Speight (2018a).

**Distribution.** Mediterranean Europe, Turkey, Bulgaria, Romania, Ukraine, and Transcaucasia.

***Eumerus armenorum* Stackelberg, 1960**

**Reference.**Peck (1988) listed it only from Armenia; Barkalov and Mutin (2018) listed it from Transcaucasia.

**Remarks.** There is not a specific record from Georgia.

**Distribution.** Described from Armenia.

***Eumerus caucasicus* Stackelberg, 1952**

**Reference.**Stackelberg (1952); Stackelberg (1961); Peck (1988); Gudjabidze (2002).

**New records.** GEORGIA • 5♂ 3♀; L53, 1 Aug 2001, J.-H. Stuke leg.; ZFMK-DIP-00058236, ZFMK-DIP-00058237, ZFMK-DIP-00058238, ZFMK-DIP-00058239, ZFMK-DIP-00058240, ZFMK-DIP-00058241, ZFMK-DIP-00058242, ZFMK-DIP-00058243.

**Distribution.** Georgia.

**Remarks.** This species was described based on a single male. We did not study the holotype, but our specimens fit the original description and key out to this species using the identification key by Stackelberg (1961). The females are identified as *E. caucasicus* based on the sampling event, plus we could not key them our properly using Stackelberg (1961). The studied specimens are the first records for this species since its original description. Moreover, this is the first report of a female of this species.

***Eumerus clavatus* Becker, 1921**

**Reference.**Gudjabidze (2002); Speight (2018a).

**Distribution.** Europe, Transcaucasia, and North Africa.

***Eumerus falsus* Becker, 1922**

**Reference.**Peck (1988).

**Distribution.** Transcaucasia, Turkey, Israel, Iran, and Central Palaearctic.

***Eumerus flavitarsis* Zetterstedt, 1843**

**Reference.**Tóth (1986); Peck (1988).

**New records.** GEORGIA • 1♀; L39, 23–26 Jul 2018, malaise trap, X. Mengual, M. Espeland, B. Thormann leg.; ZFMK-DIP-00054125 = ZFMK-TIS-8000884; • 1♀; L71, 30 Jun–14 Jul 2018, malaise trap, GGBC-members leg.; ZFMK-TIS-8002790.

**Genetics.** We sequenced two specimens (MN621962, MN621963) and their COI sequences have an uncorrected pairwise distance of 0.15%. The BIN for these specimens is BOLD:AAQ1830.

**Distribution.** Palaearctic.

***Eumerus funeralis* Megerle in Meigen, 1822**

**Reference.**Stackelberg (1961) from Caucasus as *Eumerus tuberculatus* Rondani, 1857; Peck (1988) as *E. tuberculatus*.

**Distribution.** Palaearctic; but introduced in North and South America, Australia, and New Zealand.

**Remarks.**Speight et al. (1998) reinstated the name *E. funeralis* for the taxon previously known as *E. tuberculatus* in recent literature.

***Eumerus graecus* Becker, 1921**

**Reference.**Peck (1988); Speight (2018a).

**Distribution.** Malta, Bulgaria, Greece, Turkey, and Transcaucasia.

***Eumerus grandis* Meigen, 1822**

**Reference.**Stackelberg (1961) from Armenia as *Eumerus annulatus* (Panzer, 1798); Peck (1988) from Armenia; Speight (2018a) from Armenia.

**New records.** GEORGIA • 1♂; L65, 23 Jul 2001, J.-H. Stuke leg.; ZFMK-DIP-00058235.

**Distribution.** Europe, Transcaucasia, and known from Mongolia and China.

**Remarks.** Reported for Georgia for the first time.

***Eumerus longicornis* Loew, 1855**

**Reference.**Peck (1988); Speight (2018a).

**Distribution.** Central Europe and Transcaucasia.

Remarks: Records from the Caucasus require confirmation after Doczkal (1996b) (Speight (2018a).

***Eumerus niveitibia* Becker, 1921**

**Reference.**Peck (1988); Speight (2018a).

**Distribution.** Bulgaria, Greece, Egypt, and Caucasus Mountains.

***Eumerus ornatus* Meigen, 1822**

**Reference.**Tóth (1986); Peck (1988); Gudjabidze (2002); Barkalov and Mutin (2018).

**New records.** GEORGIA • 1♀; L2, 16 Jun 2018, S. Bot leg.

**Distribution.** Western Palaearctic.

***Eumerus ovatus* Loew, 1848**

**Reference.**Gudjabidze (2002); Speight (2018a).

**Distribution.** Southern and Eastern Europe and Caucasus Mountains.

***Eumerus sogdianus* Stackelberg, 1952**

**Reference.**Stackelberg (1952); Stackelberg (1961) from Transcaucasia; Peck (1988) from Georgia; Gudjabidze (2002) as *Eumerus sogdianus* Shtakleberg, 1956 [sic]; Barkalov and Mutin (2018).

**Distribution.** Palaearctic.

***Eumerus strigatus* (Fallén, 1817)**

**Reference.**Gudjabidze (2002) as *Eumerus strigatus* (Fallen, 1917) [sic].

**Distribution.** Palaearctic; introduced in North America, Australia, and New Zealand.

***Eumerus sulcitibius* Rondani, 1868**

**Reference.**Peck (1988) listed it only from Azerbaijan; Barkalov and Mutin (2018) listed it from Transcaucasia.

**New records.** GEORGIA • 9♂; L39, 23–26 Jul 2018, malaise trap, X. Mengual, M. Espeland, B. Thormann leg.; ZFMK-DIP-00054114, ZFMK-DIP-00054115 = ZFMK-TIS-8000881, ZFMK-DIP-00054116, ZFMK-DIP-00054117, ZFMK-DIP-00054118, ZFMK-DIP-00054119, ZFMK-DIP-00054121, ZFMK-DIP-00054123, ZFMK-DIP-00054124.

**Genetics.** We were able to sequence one specimen (MN621964), and its COI barcode sequence has 99.83% similarity with another COI barcode of a specimen of *E. sulcitibius* from Greece (KX083387). The BIN for these specimens is BOLD:ADW8728.

**Distribution.** Mediterranean Basin, Turkey to Azerbaijan.

**Remarks.** Reported for Georgia for the first time.

***Eumerus tricolor* (Fabricius, 1798)**

**Reference.**Stackelberg (1961) from Transcaucasia; Peck (1988) from Armenia; Gudjabidze (2002) as *Eumerus tricolor* Meigen, 1822 [sic]; Barkalov and Mutin (2018).

**Distribution.** Palaearctic.

***Eumerus turanicus* Stackelberg, 1952**

**New records.** GEORGIA • 1♂; L39, 23–26 Jul 2018, malaise trap, X. Mengual, M. Espeland, B. Thormann leg.; ZFMK-DIP-00054126 = ZFMK-TIS-8000880.

**Genetics.** We sequenced the single collected male (MN621965), whose COI barcode has a similarity of 94.65% with *E. amoenus*. This species was not previously registered in BOLD systems.

**Distribution.** Kyrgyzstan and Tajikistan.

**Remarks.** Reported for Georgia for the first time.

**Eupeodes (Eupeodes) bucculatus (Rondani, 1857)**

**New records.** GEORGIA • 1♀; L3, 17 Jun 2018, S. Bot leg.

**Distribution.** Europe.

**Remarks.** Reported for Georgia for the first time.

**Eupeodes (Eupeodes) corollae (Fabricius, 1794)**

**Reference.**Levitin (1962) as *Syrphus corollae* F.; Tóth (1986) as *Metasyrphus corollae* (Fabricius, 1794); Gudjabidze (2002) as *Syrphus corollae* (Fallen, 1817) [sic].

**New records.** GEORGIA • 1♂; L2, 16 Jun 2018, S. Bot leg.; • 1♂; L3, 17 Jun 2018, S. Bot leg.; • 1♀; L8, 20 Jun 2018, S. Bot leg.; • 2♂; L10, 22 Jun 2018, S. Bot leg.; • 2♀; L11, 23 Jun 2018, S. Bot leg.; • 1♀; L12, 24 Jun 2018, S. Bot leg.; • 1♀; L20, 30 Jun 2018, S. Bot leg.; • 1♂ 1♀; L20, 2 Jul 2018, S. Bot leg.; • 1♂; L27, 19 Jul 2018, X. Mengual leg.; ZFMK-DIP-00053850 = ZFMK-TIS-8005593; • 2♀; L31, 20 Jul 2018, X. Mengual leg.; ZFMK-DIP-00054096 = ZFMK-TIS-8000875, ZFMK-DIP-00054097 = ZFMK-TIS-8000876; • 1♀; L49, 4 Aug 2001, J.-H. Stuke leg.; ZFMK-DIP-00057498; • 1♂ 1♀; L52, 30 Jul 2001, J.-H. Stuke leg.; ZFMK-DIP-00057491, ZFMK-DIP-00057495; • 2♀; L53, 1 Aug 2001, J.-H. Stuke leg.; ZFMK-DIP-00057496, ZFMK-DIP-00057497; • 2♂ 3♀; L57, 3 Aug 2001, J.-H. Stuke leg.; ZFMK-DIP-00057490, ZFMK-DIP-00057492, ZFMK-DIP-00057493, ZFMK-DIP-00057494, ZFMK-DIP-00057499;• 1♀; L69, 18 Jul 2018, A. Reimann leg.; MTD-Dip-A-R-4502; • 6♂ 11♀; L70, 30 Jun–14 Jul 2018, malaise trap, GGBC-members leg.; ZFMK-TIS-8002713, ZFMK-TIS-8002714, ZFMK-TIS-8002715, ZFMK-TIS-8002716, ZFMK-DIP-00061280, ZFMK-DIP-00061281, ZFMK-DIP-00061282, ZFMK-DIP-00061283, ZFMK-DIP-00061284, ZFMK-DIP-00061285, ZFMK-DIP-00061286, ZFMK-DIP-00061287, ZFMK-DIP-00061288, ZFMK-DIP-00061289, ZFMK-DIP-00061290, ZFMK-DIP-00061291, ZFMK-DIP-00061292; • 2♂ 10♀; L70, 30 Jun–14 Jul 2018, malaise trap, GGBC-members leg.; MTD-Dip-A-R-4570, MTD-Dip-A-R-4578; • 5♀; L71, 30 Jun–14 Jul 2018, malaise trap, GGBC-members leg.; ZFMK-TIS-8002738, ZFMK-TIS-8002739, ZFMK-DIP-00061293, ZFMK-DIP-00061294, ZFMK-DIP-00061295; • 1♂ 3♀; L71, 30 Jun–14 Jul 2018, malaise trap, GGBC-members leg.; MTD-Dip-A-R-4568.

**Genetics.** We sequenced seven specimens (MN621966, MN621967, MN621968, MN621969, MN621970, MN621971, MN621972). The obtained sequences differ from 0 to 2.06% among them. The BIN for this species has a problem in BOLD systems and refers to a hemipteran species.

**Distribution.** Palaearctic, Afrotropical Region, and Taiwan.

**Eupeodes (Eupeodes) flaviceps (Rondani, 1857)**

**Reference.**Peck (1988); Gudjabidze (2002) as *Syrphus braueri* Egger, 1858; Speight (2018a).

**Distribution.** Europe and Transcaucasia.

**Eupeodes (Eupeodes) goeldlini Mazánek, Láska & Bičík, 1999**

**New records.** GEORGIA • 1♂; L21, 2 Jul 2018, S. Bot leg.; • 4♂; L51, 24 Jul 2001, J.-H. Stuke leg.; ZFMK-DIP-00057506, ZFMK-DIP-00057507, ZFMK-DIP-00057507, ZFMK-DIP-00057506.

**Distribution.** Europe and European parts of Russia.

**Remarks.** Reported for Georgia for the first time.

**Eupeodes (Eupeodes) latifasciatus (Macquart, 1829)**

**Reference.**Levitin (1962) as *Syrphus latifasciatus* Macq.; Tóth (1986) as *Metasyrphus latifasciatus* (Macquart, 1829); Gudjabidze (2002) as *Syrphus latifasciatus* Macquart, 1827 [sic].

**New records.** GEORGIA • *1♂; L9, 21 Jun 2018, S. Bot obs.; • 1♂; L10, 22 Jun 2018, S. Bot leg.; • 1♂; L17, 28 Jun 2018, S. Bot leg.; • 2♂; L49, 4 Aug 2001, J.-H. Stuke leg.; ZFMK-DIP-00057595, ZFMK-DIP-00057596; • 1♂ 3♀; L52, 30 Jul 2001, J.-H. Stuke leg.; ZFMK-DIP-00057594, ZFMK-DIP-00057597, ZFMK-DIP-00057598, ZFMK-DIP-00057599; • 2♂; L64, 23 Jul 2001, J.-H. Stuke leg.; ZFMK-DIP-00057592, ZFMK-DIP-00057593.

**Distribution.** Holarctic and India.

**Eupeodes (Eupeodes) luniger (Meigen, 1822)**

**Reference.**Levitin (1962) as *Syrphus luniger* Mg.; Tóth (1986) as *Metasyrphus luniger* (Meigen, 1822); Gudjabidze (2002) as *Syrphus luniger* Meigen, 1822.

**New records.** GEORGIA • 1♀; L11, 29 Jun 2018, S. Bot leg.; • 1♀; L34, 22 Jul 2018, X. Mengual leg.; ZFMK-DIP-00053851 = ZFMK-TIS-8005594; • 1♀; L54, 2 Aug 2001, J.-H. Stuke leg.; ZFMK-DIP-00057502; • 1♀; L55, 31 Jul 2001, J.-H. Stuke leg.; ZFMK-DIP-00057503; • 1♂; L57, 3 Aug 2001, J.-H. Stuke leg.; ZFMK-DIP-00057504.

**Genetics.** One specimen was sequenced (MN621973), with identical COI sequence to other published specimens of the same species (MF446537 from Germany; KF939552 and KF939551 from Spain). In BOLD systems, the BIN for *E. luniger* (BOLD:AAB2384) comprises specimens identified as different species of the same genus.

**Distribution.** Palaearctic and northern India.

**Eupeodes (Eupeodes) nitens (Zetterstedt, 1843)**

**Reference.**Gudjabidze (2002) as *Syrphus nitens* Zetterstendt, 1843 [sic].

**Distribution.** Palaearctic.

**Eupeodes (Eupeodes) nuba (Wiedemann, 1830)**

**Reference.**Peck (1988) as Metasyrphus (Metasyrphus) nuba (Wiedemann, 1830); Barkalov and Mutin (2018).

**New records.** GEORGIA • 1♀; L57, 3 Aug 2001, J.-H. Stuke leg.; ZFMK-DIP-00057505.

**Distribution.** Canary Isles, Europe, Transcaucasia, Central Palaearctic to Mongolia.

***Fagisyrphus cinctus* (Fallén, 1817)**

**Reference.**Levitin (1962) as *Syrphus cinctus* Flln.; Tóth (1986) as *Meligramma cinctus* Fallén, 1817; Peck (1988) as Melangyna (Meligramma) cincta (Fallén, 1817); Gudjabidze (2002) as *Syrphus cinctus* Fallén, 1817.

**New records.** GEORGIA • 1♂; L31, 23 Jul 2018, A. Reimann leg.; MTD-Dip-A-R-4528; • 1♂; L38, 25 Jul 2018, X. Mengual leg.; ZFMK-DIP-00054099 = ZFMK-TIS-8001001; • 1♀; L70, 30 Jun–14 Jul 2018, malaise trap, GGBC-members leg.; ZFMK-TIS-8002712.

**Genetics.** We sequenced two specimens (MN621974, MN621975) which have exactly the same COI barcode. The Barcode Index Number Registry lists one BIN for this taxon (BOLD:AAQ4086) with an average variation of p-distance of 0.05% within the BIN (0.58% max) and 6.05% p-distance to the nearest neighbour, *Meligramma triangulifera* (Zetterstedt, 1843) (BOLD:AAZ1912).

**Distribution.** Europe, European parts of Russia, and Crimea.

***Ferdinandea aurea* Rondani, 1844**

**Reference.**Peck (1988); Gudjabidze (2002) as *Ferdinandea aurea* Rondani, 1861 [sic].

**Distribution.** Southern Europe and Transcaucasia.

***Ferdinandea cuprea* (Scopoli, 1763)**

**Reference.**Levitin (1962); Peck (1988); Gudjabidze (2002).

**Distribution.** Palaearctic.

***Ferdinandea ruficornis* (Fabricius, 1775)**

**Reference.**Peck (1988); Speight (2018a).

**Distribution.** Palaearctic.

***Hammerschmidtia ferruginea* (Fallén, 1817)**

**Reference.**Peck (1988); Gudjabidze (2002).

**New records.** GEORGIA • 3♂; L8, 20 Jun 2018, S. Bot leg.

**Distribution.** Palaearctic, Western Coast of North America, and Alaska.

***Helophilus continuus* Loew, 1854**

**Reference.**Peck (1988); Gudjabidze (2002) as *Helophilus continuus* Loew, 1846 [sic]; Speight (2018a).

**New records.** GEORGIA • 1♂; L24, 17 Jul 2018, X. Mengual leg.; ZFMK-DIP-00053802 = ZFMK-TIS-8005572; • 1♀; L25, 18 Jul 2018, X. Mengual leg.; ZFMK-DIP-00054000 = ZFMK-TIS-8003448; • 1♂; L31, 20 Jul 2018, X. Mengual leg.; ZFMK-DIP-00054208; • 2♂ 2♀; L33, 22 Jul 2018, X. Mengual leg.; ZFMK-DIP-00053804, ZFMK-DIP-00053807, , ZFMK-DIP-00053999 = ZFMK-TIS-8003437, ZFMK-DIP-00054013; • 4♂ 1♀; L33, 23 Jul 2018, X. Mengual leg.; ZFMK-DIP-00053801, ZFMK-DIP-00053805, ZFMK-DIP-00053806, ZFMK-DIP-00053809 = ZFMK-TIS-8005579, ZFMK-DIP-00054012 = ZFMK-TIS-8000983; • 1♀; L37, 25 Jul 2018, B. Thormann leg.; ZFMK-DIP-00054010 = ZFMK-TIS-8004108; • 1♀; L38, 25 Jul 2018, J. Thormann leg.; ZFMK-DIP-00054011 = ZFMK-TIS-8004028; • 1♂; L38, 25 Jul 2018, X. Mengual leg.; ZFMK-DIP-00053803.

**Genetics.** We sequenced two specimens (MN621976, MN621977), whose COI barcodes differ 0.15%. One sequence (MN621977) matches another sequence present in BOLD from Altai Mountains, Russia. The BIN for all these specimens is BOLD:ACO6169 and the nearest neighbour in BOLD systems is *Helophilus lapponicus* Wahlberg, 1844 (BOLD:ACE4226).

**Distribution.** Eastern Europe, Transcaucasia, and through Russia to Kamchatka.

***Helophilus pendulus* (Linnaeus, 1758)**

**Reference.**Peck (1988); Gudjabidze (2002).

**New records.** GEORGIA • 1♂L4, 18 Jun 2018, S. Bot leg.; • *1♂; L21, 1 Jul 2018, S. Bot obs.; • 1♀; L24, 17 Jul 2018, X. Mengual leg.; ZFMK-DIP-00053793 = ZFMK-TIS-8005570.

**Genetics.** We sequenced one specimen (MN621978) and its COI barcode has 100% similarity with other published sequences of this species. The Barcode Index Number Registry lists 1 BIN for this taxon (BOLD:AAI6747) with an average p-distance of 0.25% within BIN (1.65% max.) and a p-distance of 1.96% to the nearest neighbour in BOLD systems, *Helophilus sapporensis* Matsumura, 1911 (BOLD:ACO5411).

**Distribution.** Palaearctic.

***Helophilus trivittatus* (Fabricius, 1805)**

**Reference.**Radde (1899); Peck (1988) as *Helophilus parallelus* (Harris, 1776); Gudjabidze (2002) as *Helophilus trivitatus* (Fabricius, 1777) [sic].

**New records.** GEORGIA • 1♀; L8, 20 Jun 2018, S. Bot leg.; • 2♂ 1♀; L31, 20 Jul 2018, X. Mengual leg.; ZFMK-DIP-00053794 = ZFMK-TIS-8005571, ZFMK-DIP-00054003 = ZFMK-TIS-8003442, ZFMK-DIP-00054002 = ZFMK-TIS-8003444; • 1♀; L31, 23 Jul 2018, A. Reimann leg.; MTD-Dip-A-R-4510; • 1♀; L35, 24 Jul 2018, X. Mengual leg.; ZFMK-DIP-00053800; • 1♂ 2♀; L37, 25 Jul 2018, B. Thormann leg.; ZFMK-DIP-00054009 = ZFMK-TIS-8004099, ZFMK-DIP-00054006 = ZFMK-TIS-8004113, ZFMK-DIP-00054007 = ZFMK-TIS-8004098; • 3♂ 4♀; L37, 25 Jul 2018, X. Mengual leg.; ZFMK-DIP-00053795, ZFMK-DIP-00053796, ZFMK-DIP-00054004 = ZFMK-TIS-8003425, ZFMK-DIP-00054001 = ZFMK-TIS-8003424, ZFMK-DIP-00053797, ZFMK-DIP-00053798, ZFMK-DIP-00053799 = ZFMK-TIS-8005578; • 2♀; L38, 25 Jul 2018, B. Thormann leg.; ZFMK-DIP-00054005 = ZFMK-TIS-8004228, ZFMK-DIP-00054008 = ZFMK-TIS-8004230; • 1♀; L60, 26 Jul 2001, J.-H. Stuke leg.; ZFMK-DIP-00057999; • 1♂; L69, 18 Jul 2018, A. Reimann leg.; MTD-Dip-A-R-4497 = ZFMK-TIS-8002661.

**Genetics.** We sequenced three specimens (MN621979, MN621980, MN621981) and the COI barcodes were very similar (0–0.53% p-distance difference). The BIN for these specimens is BOLD:ABY6684.

**Distribution.** Palaearctic.

**Remarks.**Van der Goot (1981) used *Helophilus trivittatus* for the taxon also known as *Helophilus parallelus*. Later, Van der Goot (1986) explained that the name *Helophilus parallelus* was wrongly applied to the taxon *Helophilus trivittatus*.

***Heringia heringi* (Zetterstedt, 1843)**

**Reference.**Peck (1988).

**Distribution.** Palaearctic.

***Heringia senilis* Sack, 1938**

**Reference.**Tóth (1986); Peck (1988).

**Remarks.** Specimens of this taxon are often identified as *Heringia heringi* due to the lack of diagnosable differences (Claußen et al. 1994).

***Ischiodon scutellaris* (Fabricius, 1805)**

**Reference.**Peck (1988); Gudjabidze (2002) as *Ischiodon scutellare* Fabricius, 1794 [sic]; Mengual (2018).

**Distribution.** Greece, eastwards to Caucasus, Kazakhstan, Iran, Arabian Peninsula south to Indomalayan Region, Taiwan, Australasian and Oceanian regions except Hawaii, China, and Japan.

***Lapposyrphus lapponicus* (Zetterstedt, 1838)**

**Reference.**Levitin (1962) as *Syrphus eapponicus* Zett. [sic]; Tóth (1986); Peck (1988) as Metasyrphus (Lapposyrphus) lapponicus (Zetterstedt, 1838); Gudjabidze (2002) as *Syrphus lapponicus* Zetterstendt, 1843 [sic].

**New records.** GEORGIA • 1♂ 1♀; L2, 16 Jun 2018, S. Bot leg.; • 1♀; L3, 17 Jun 2018, S. Bot leg.; • 1♂; L4, 18 Jun 2018, S. Bot leg.; • 1♀; L6, 19 Jun 2018, S. Bot leg.; • 1♂; L8, 20 Jun 2018, S. Bot leg.; • 1♂; L16, 27 Jun 2018, S. Bot leg.; • 1♂; L17, 28 Jun 2018, S. Bot leg.; • 1♂; L19, 29 Jun 2018, S. Bot leg.

**Distribution.** Palaearctic, also mentioned from Alaska to California.

***Lejogaster metallina* (Fabricius, 1781)**

**Reference.**Peck (1988); Gudjabidze (2002) as *Liogaster metallina* Fabricius, 1777 [sic].

**New records.** GEORGIA • 1♂; L1, 16 Jun 2018, S. Bot leg.; • 10♂ 5♀; L46, 24 Jul 2001, J.-H. Stuke leg.; ZFMK-DIP-00057936, ZFMK-DIP-00057937, ZFMK-DIP-00057938, ZFMK-DIP-00057939, ZFMK-DIP-00057940, ZFMK-DIP-00057941, ZFMK-DIP-00057942, ZFMK-DIP-00057943, ZFMK-DIP-00057944, ZFMK-DIP-00057949, ZFMK-DIP-00057945, ZFMK-DIP-00057946, ZFMK-DIP-00057947, ZFMK-DIP-00057948, ZFMK-DIP-00057950; • 1♀; L49, 4 Aug 2001, J.-H. Stuke leg.; ZFMK-DIP-00057952; • 9♂ 6♀; L50, 4 Aug 2001, J.-H. Stuke leg.; ZFMK-DIP-00057953, ZFMK-DIP-00057954, ZFMK-DIP-00057955, ZFMK-DIP-00057956, ZFMK-DIP-00057957, ZFMK-DIP-00057958, ZFMK-DIP-00057959, ZFMK-DIP-00057960, ZFMK-DIP-00057961, ZFMK-DIP-00057962, ZFMK-DIP-00057963, ZFMK-DIP-00057964, ZFMK-DIP-00057965, ZFMK-DIP-00057966, ZFMK-DIP-00057967; • 1♀; L53, 1 Aug 2001, J.-H. Stuke leg.; ZFMK-DIP-00057951.

**Distribution.** Palaeartic.

***Lejogaster tarsata* (Megerle in Meigen, 1822)**

**Reference.**Peck (1988) as *Lejogaster splendida* (Meigen, 1822); Gudjabidze (2002) as *Liogaster splendida* Meigen, 1822 [sic].

**New records.** GEORGIA • 3♂ 1♀; L63, 23 Jul 2001, J.-H. Stuke leg.; ZFMK-DIP-00057968, ZFMK-DIP-00057969, ZFMK-DIP-00057970, ZFMK-DIP-00057971.

**Distribution.** Palaeartic.

**Remarks.**Maibach et al. (1994b) reinstated the name *Lejogaster tarsata* (Megerle in Meigen, 1822) for the taxon referred as *Lejogaster splendida* (Megerle in Meigen, 1822) in recent literature.

**Leucozona (Ischyrosyrphus) glaucia (Linnaeus, 1758)**

**Reference.**Gudjabidze (2002) as *Ischyrosyrphus glaucius* Linnaeus, 1758 [sic].

**New records.** GEORGIA • 1♂; L16, 27 Jun 2018, S. Bot leg.; 1♀; • L20, 30 Jun 2018, S. Bot leg.

**Distribution.** Palaearctic.

**Leucozona (Ischyrosyrphus) laternaria (Müller, 1776)**

**Reference.**Gudjabidze (2002) as *Ischyrosyrphus laternarius* Muller.

**Distribution.** Palaearctic.

**Leucozona (Leucozona) lucorum (Linnaeus, 1758)**

**Remarks.** Since the morphological characters, which help to distinguish between *L. lucorum* and *L. nigripila*, have only recently been clarified by Doczkal (2000b), it is unclear whether *L. lucorum* occurs in sympatry with *L. nigripila* in the Caucasus or whether all records should refer to *L. nigripila*.

**Leucozona (Leucozona) nigripila Mik, 1888**

**Reference.**Tóth (1986) as *Leucozona lucorum* Linnaeus, 1758 [sic]; Peck (1988) as *Leucozona lucorum* (Linnaeus, 1758); Gudjabidze (2002) as *Leucozona lucorom* (Linnaeus, 1758) [sic].

**New records.** GEORGIA • 1♂; L6, 19 Jun 2018, S. Bot leg.; • 1♀; L19, 29 Jun 2018, S. Bot leg.; • 1♀; L35, 24 Jul 2018, X. Mengual leg.; ZFMK-DIP-00054014 = ZFMK-TIS-8000861; • 2♀; L57, 3 Aug 2001, J.-H. Stuke leg.; ZFMK-DIP-00057500, ZFMK-DIP-00057501.

**Genetics.** We were able to sequence one specimen (MN621982). The obtained COI barcode has a similiraty of > 99.4% with published sequences of *L. lucorum*. The BIN in BOLD systems comprising *L. nigripila* is BOLD:AAK9203, which also has specimens of *Leucozona inopinata* Doczkal, 2000 and *L. lucorum* (average distance within BIN of 0.5%, and 0.19% of maximum distance). The nearest neighbour in BOLD systems is *Leucozona americana* Curran, 1923 (BOLD:ACE4604; 1.26% p-distance).

**Distribution.** Northern Caucasus and Transcaucasia.

**Remarks.** Here we have listed under *L. nigripila* all the previous records of *L. lucorum*. Since the morphological characters, which help to distinguish between *L. lucorum* and *L. nigripila*, have only recently been clarified by Doczkal (2000b), it is unclear if *L. lucorum* occurs in sympatry with *L. nigripila* in the Caucasus, but it is very unlikely. This species was described from Northern Caucasus (Circassia) and Doczkal (2000b) reported it from there and from Kussari (= Qusar, Azerbaijan). The specimen from Kussari is very likely to be the female that Mik (1888) mentioned from the Caucasus. Reported for Georgia for the first time.

***Mallota fuciformis* (Fabricius, 1794)**

**Reference.**Peck (1988).

**Distribution.** Central and Southern Europe, European parts of Russia, Transcaucasia, and Iran.

***Megasyrphus erraticus* (Linnaeus, 1758)**

**Reference.**Peck (1988) as *Megasyrphus annulipes* (Zetterstedt, 1838); Gudjabidze (2002) as *Syrphus annulipes* (Zetterstedt, 1838).

**Distribution.** Palaearctic, including Nepal.

**Remarks.**Thompson et al. (1982) synonymised *Scaeva annulipes* Zetterstedt, 1838 under *Musca erratica* Linnaeus, 1758.

**Melangyna (Melangyna) compositarum (Verrall, 1873)**

**Reference.**Peck (1988); Gudjabidze (2002) as *Syrphus compositarum* Verall [sic].

**Distribution.** Holarctic.

**Melangyna (Melangyna) lasiophthalma (Zetterstedt, 1843)**

**Reference.**Peck (1988); Gudjabidze (2002) as *Syrphus lasiophthalmus* Zetterstend, 1843 [sic].

**Distribution.** Holarctic.

**Melangyna (Melangyna) umbellatarum (Fabricius, 1794)**

**Reference.**Peck (1988); Gudjabidze (2002) as *Syrphus umbellatarum* (Fallen, 1817) [sic].

**New records.** GEORGIA • 2♂; L16, 27 Jun 2018, S. Bot leg.; • 1♂; L17, 28 Jun 2018, S. Bot leg.

**Distribution.** Holarctic.

***Melanogaster nuda* (Macquart, 1829)**

**Reference.**Peck (1988) as *Chrysogaster viduata* (Linnaeus, 1758); Gudjabidze (2002) as *Chrysogaster viduata* Linnaeus, 1758 [sic].

**New records.** GEORGIA • 1♂; L10, 22 Jun 2018, S. Bot leg.; • 2♂; L11, 23 Jun 2018, S. Bot leg.; • 2♀; L11, 29 Jun 2018, S. Bot leg.

**Distribution.** Western Palaearctic.

**Remarks.** While fixing the name *Musca viduata* Linnaeus, 1758 with a lectotype designation, Thompson et al. (1982) suggested to apply the name *Musca lucida* Scopoli, 1763 for a taxon known as *Chrysogaster viduata* (Linnaeus, 1758) by various authors. Maibach et al. (1994b) established that the name *Musca lucida* was wrongly applied to this species and introduced *Melanogaster nuda* (Macquart, 1829) as replacement name for this taxon.

***Melanogaster tumescens* (Loew, 1873)**

**Reference.**Peck (1988).

**Distribution.** European parts of Russia and Transcaucasia.

**Remarks.**Maibach et al. (1994a) placed this taxon under the genus *Melanogaster* Rondani, 1857. This species is not referred to in recent literature and its status is unclear (Speight 2018a).

***Melanostoma mellinum* (Linnaeus, 1758)**

**Reference.**Radde (1899); Levitin (1962); Tóth (1986); Gudjabidze (2002).

**New records.** GEORGIA • 1♂ 2♀; L3, 17 Jun 2018, S. Bot leg.; • 1♀; L4, 18 Jun 2018, S. Bot leg.; • 1♀; L6, 19 Jun 2018, S. Bot leg.; • 1♀; L7, 19 Jun 2018, S. Bot leg.; • 1♂ 2♀; L10, 22 Jun 2018, S. Bot leg.; • 1♀; L10, 22 Jun 2018, S. Bot leg.; • 1♂ 2♀; L12, 24 Jun 2018, S. Bot leg.; • 1♀; L16, 27 Jun 2018, S. Bot leg.; • 1♀; L17, 28 Jun 2018, S. Bot leg.; • 1♂; L18, 28 Jun 2018, S. Bot leg.; • 1♀; L20, 1 Jul 2018, S. Bot leg.; • 2♀; L28, 19 Jul 2018, X. Mengual leg.; ZFMK-DIP-00053936; ZFMK-DIP-00054134 = ZFMK-TIS-8000976; • 3♂; L29, 19 Jul 2018, X. Mengual leg.; ZFMK-DIP-00053921, ZFMK-DIP-00053925, ZFMK-DIP-00054132 = ZFMK-TIS-8001011; • 1♂; L30, 19 Jul 2018, X. Mengual leg.; ZFMK-DIP-00053937 = ZFMK-TIS-8005525; • 5♂ 1♀; L31, 20 Jul 2018, X. Mengual leg.; ZFMK-DIP-00053923, ZFMK-DIP-00053924 = ZFMK-TIS-8005524, ZFMK-DIP-00054137, ZFMK-DIP-00054138, ZFMK-DIP-00054141, ZFMK-DIP-00054140; • 4♂ 1♀; L32, 22 Jul 2018, X. Mengual leg.; ZFMK-DIP-00053922, ZFMK-DIP-00053927, ZFMK-DIP-00053928, ZFMK-DIP-00053929, ZFMK-DIP-00054135 = ZFMK-TIS-8001006; • 2♂ 2♀; L33, 22 Jul 2018, X. Mengual leg.; ZFMK-DIP-00053920, ZFMK-DIP-00053926, ZFMK-DIP-00054139, ZFMK-DIP-00054143; • 1♀; L34, 23 Jul 2018, J. and B. Thormann leg.; ZFMK-DIP-00054136 = ZFMK-TIS-8004305 • 1♀; L34, 22 Jul 2018, X. Mengual leg.; ZFMK-DIP-00054131 = ZFMK-TIS-8000992; • 3♂ 1♀; L35, 24 Jul 2018, X. Mengual leg.; ZFMK-DIP-00053931, ZFMK-DIP-00053932, ZFMK-DIP-00053933, ZFMK-DIP-00053935 = ZFMK-TIS-8005530; • 1♀; L36, 24 Jul 2018, X. Mengual leg.; ZFMK-DIP-00053934; • 1♂; L37, 25 Jul 2018, X. Mengual leg.; ZFMK-DIP-00053930; • 1♂; L38, 25 Jul 2018, J. Thormann leg.; ZFMK-DIP-00054133 = ZFMK-TIS-8004025; • 2♀; L39, 23–26 Jul 2018, malaise trap, X. Mengual, M. Espeland, B. Thormann leg.; ZFMK-DIP-00054142, ZFMK-DIP-00054144; • 4♀; L41, 19 Jul 2018, B. Thormann leg.; ZFMK-DIP-00054127 = ZFMK-TIS-8000268, ZFMK-DIP-00054128 = ZFMK-TIS-8000271, ZFMK-DIP-00054129 = ZFMK-TIS-8000270, ZFMK-DIP-00054130 = ZFMK-TIS-8000269; • 1♀; L42, 25–28 Jul 2018, B. Wipfler leg.; ZFMK-DIP-00054210; • 8♂ 10♀; L46, 24 Jul 2001, J.-H. Stuke leg.; ZFMK-DIP-00057756, ZFMK-DIP-00057757, ZFMK-DIP-00057758, ZFMK-DIP-00057759, ZFMK-DIP-00057760, ZFMK-DIP-00057761, ZFMK-DIP-00057762, ZFMK-DIP-00057763, ZFMK-DIP-00057524, ZFMK-DIP-00057525, ZFMK-DIP-00057526, ZFMK-DIP-00057527, ZFMK-DIP-00057528, ZFMK-DIP-00057529, ZFMK-DIP-00057530, ZFMK-DIP-00057531, ZFMK-DIP-00057532, ZFMK-DIP-00057576; • 14♂ 13♀; L47, 25 Jul 2001, J.-H. Stuke leg.; ZFMK-DIP-00057764, ZFMK-DIP-00057765, ZFMK-DIP-00057766, ZFMK-DIP-00057767, ZFMK-DIP-00057768, ZFMK-DIP-00057769, ZFMK-DIP-00057770, ZFMK-DIP-00057771, ZFMK-DIP-00057772, ZFMK-DIP-00057773, ZFMK-DIP-00057774, ZFMK-DIP-00057775, ZFMK-DIP-00057776, ZFMK-DIP-00057777, ZFMK-DIP-00057511, ZFMK-DIP-00057512, ZFMK-DIP-00057513, ZFMK-DIP-00057514, ZFMK-DIP-00057515, ZFMK-DIP-00057516, ZFMK-DIP-00057517, ZFMK-DIP-00057518, ZFMK-DIP-00057519, ZFMK-DIP-00057520, ZFMK-DIP-00057521, ZFMK-DIP-00057522, ZFMK-DIP-00057523; • 7♂ 13♀; L48, 24 Jul 2001, J.-H. Stuke leg.; ZFMK-DIP-00057614, ZFMK-DIP-00057615, ZFMK-DIP-00057616, ZFMK-DIP-00057617, ZFMK-DIP-00057618, ZFMK-DIP-00057619, ZFMK-DIP-00057740, ZFMK-DIP-00057544, ZFMK-DIP-00057545, ZFMK-DIP-00057546, ZFMK-DIP-00057547, ZFMK-DIP-00057548, ZFMK-DIP-00057549, ZFMK-DIP-00057550, ZFMK-DIP-00057551, ZFMK-DIP-00057552, ZFMK-DIP-00057553, ZFMK-DIP-00057554, ZFMK-DIP-00057555, ZFMK-DIP-00057556; • 1♂ 4♀; L49, 4 Aug 2001, J.-H. Stuke leg.; ZFMK-DIP-00057795, ZFMK-DIP-00057540, ZFMK-DIP-00057541, ZFMK-DIP-00057542, ZFMK-DIP-00057543; • 1♂ 1♀; L50, 4 Aug 2001, J.-H. Stuke leg.; ZFMK-DIP-00057808, ZFMK-DIP-00057575; • 11♂ 7♀; L51, 24 Jul 2001, J.-H. Stuke leg.; ZFMK-DIP-00057741, ZFMK-DIP-00057742, ZFMK-DIP-00057743, ZFMK-DIP-00057744, ZFMK-DIP-00057745, ZFMK-DIP-00057746, ZFMK-DIP-00057748, ZFMK-DIP-00057749, ZFMK-DIP-00057750, ZFMK-DIP-00057751, ZFMK-DIP-00057752, ZFMK-DIP-00057564, ZFMK-DIP-00057565, ZFMK-DIP-00057567, ZFMK-DIP-00057568, ZFMK-DIP-00057569, ZFMK-DIP-00057570, ZFMK-DIP-00057571; • 3♂ 6♀; L52, 30 Jul 2001, J.-H. Stuke leg.; ZFMK-DIP-00057753, ZFMK-DIP-00057754, ZFMK-DIP-00057755, ZFMK-DIP-00057558, ZFMK-DIP-00057559, ZFMK-DIP-00057560, ZFMK-DIP-00057561, ZFMK-DIP-00057562, ZFMK-DIP-00057563; • 15♂ 7♀; L53, 1 Aug 2001, J.-H. Stuke leg.; ZFMK-DIP-00057778, ZFMK-DIP-00057779, ZFMK-DIP-00057780, ZFMK-DIP-00057781, ZFMK-DIP-00057782, ZFMK-DIP-00057783, ZFMK-DIP-00057784, ZFMK-DIP-00057785, ZFMK-DIP-00057786, ZFMK-DIP-00057787, ZFMK-DIP-00057788, ZFMK-DIP-00057790, ZFMK-DIP-00057791, ZFMK-DIP-00057792, ZFMK-DIP-00057793, ZFMK-DIP-00057533, ZFMK-DIP-00057534, ZFMK-DIP-00057535, ZFMK-DIP-00057536, ZFMK-DIP-00057537, ZFMK-DIP-00057538, ZFMK-DIP-00057539; • 14♂ 3♀; L56, 30 Jul 2001, J.-H. Stuke leg.; ZFMK-DIP-00057600, ZFMK-DIP-00057601, ZFMK-DIP-00057602, ZFMK-DIP-00057603, ZFMK-DIP-00057604, ZFMK-DIP-00057605, ZFMK-DIP-00057606, ZFMK-DIP-00057607, ZFMK-DIP-00057608, ZFMK-DIP-00057609, ZFMK-DIP-00057610, ZFMK-DIP-00057611, ZFMK-DIP-00057612, ZFMK-DIP-00057613, ZFMK-DIP-00057572, ZFMK-DIP-00057573, ZFMK-DIP-00057574; • 2♂ 1♀; L57, 2 Aug 2001, J.-H. Stuke leg.; ZFMK-DIP -00057797, ZFMK-DIP-00057798, ZFMK-DIP-00057578; • 9♂ 1♀; L57, 3 Aug 2001, J.-H. Stuke leg.; ZFMK-DIP-00057799, ZFMK-DIP-00057800, ZFMK-DIP-00057801, ZFMK-DIP-00057802, ZFMK-DIP-00057803, ZFMK-DIP-00057804, ZFMK-DIP-00057805, ZFMK-DIP-00057806, ZFMK-DIP-00057807, ZFMK-DIP-00057557; • 1♀; L58, 27 Jul 2001, J.-H. Stuke leg.; ZFMK-DIP-00057581; • 2♀; L60, 26 Jul 2001 J.-H. Stuke leg.; ZFMK-DIP-00057579, ZFMK-DIP-00057580; • 1♀; L62, 22 Jul 2001 J.-H. Stuke leg.; ZFMK-DIP-00057584; • 2♀; L63, 23 Jul 2001, J.-H. Stuke leg.; ZFMK-DIP-00057582, ZFMK-DIP-00057583; • 1♀; L64, 23 Jul 2001, J.-H. Stuke leg.; ZFMK-DIP-00057577; • 1♂; L70, 30 Jun–14 Jul 2018, malaise trap, GGBC-members leg.; ZFMK-TIS-8002719; • 2♀; L71, 30 Jun–14 Jul 2018, malaise trap, GGBC-members leg.; ZFMK-TIS-8002753, ZFMK-TIS-8002754; • 1♂ 2♀; L72, 29 Jun–13 Jul 2018, malaise trap, GGBC-members leg.; ZFMK-TIS-8002781, ZFMK-TIS-8002783, ZFMK-TIS-8002784.

**Genetics.** We sequenced nine specimens (MN621983, MN621984, MN621985, MN621986, MN621987, MN621988, MN621989, MN621990, MN621991) and the obtained COI barcodes differ 0–1.22%.

**Distribution.** Holarctic.

**Remarks.**Haarto and Ståhls (2014) proved that different *Melanostoma* species share the same COI haplotypes among them and that this mitochondrial gene is not very useful for species identification. Speight (2018a) mentioned the possibility of a species complex under this name because it has a large phenotypic variability and ecological amplitude.

***Melanostoma orientale* (Wiedemann, 1824)**

**Reference.**Mutin and Barkalov (1999); Barkalov and Mutin (2018).

**Distribution.** Transcaucasia, Indomalayan Region, and Eastern Palaearctic.

***Melanostoma scalare* (Fabricius, 1794)**

**Reference.**Tóth (1986); Peck (1988); Gudjabidze (2002) as *Melanostoma scalare* Fabricius, 1805 [sic].

**New records.** GEORGIA • *1♂; L1, 16 Jun 2018, S. Bot obs.; • 2♀; L1, 16 Jun 2018, S. Bot leg.; • 2♀; L6, 19 Jun 2018, S. Bot leg.; • 1♀; L14, 25 Jun 2018, S. Bot leg.; • 2♀; L15, 26 Jun 2018, S. Bot leg.; • 1♂; L20, 1 Jul 2018, S. Bot leg.; • 1♂; L21, 3 Jul 2018, S. Bot leg.; • 1♂; L24, 17 Jul 2018, X. Mengual leg.; ZFMK-DIP-00053941; • 1♀; L26, 18 Jul 2018, X. Mengual leg.; ZFMK-DIP-00054161; • 2♂; L28, 19 Jul 2018, X. Mengual leg.; ZFMK-DIP-00053939 = ZFMK-TIS-8005526, ZFMK-DIP-00053940; • 4♂ 5♀; L29, 19 Jul 2018, X. Mengual leg.; ZFMK-DIP-00053938, ZFMK-DIP-00053942, ZFMK-DIP-00053943, ZFMK-DIP-00054154 = ZFMK-TIS-8001012, ZFMK-DIP-00053959, ZFMK-DIP-00053961, ZFMK-DIP-00053963, ZFMK-DIP-00053964, ZFMK-DIP-00054151 = ZFMK-TIS-8001014; • 1♀; L31, 20 Jul 2018, X. Mengual leg.; ZFMK-DIP-00053962 = ZFMK-TIS-8005531; • 1♂ 1♀; L32, 22 Jul 2018, X. Mengual leg.; ZFMK-DIP-00053950, ZFMK-DIP-00053965; • 9♂ 1♀; L33, 22 Jul 2018, X. Mengual leg.; ZFMK-DIP-00053949, ZFMK-DIP-00053951, ZFMK-DIP-00053952, ZFMK-DIP-00053953, ZFMK-DIP-00053954, ZFMK-DIP-00054153 = ZFMK-TIS-8000986, ZFMK-DIP-00054158, ZFMK-DIP-00054159, ZFMK-DIP-00054160, ZFMK-DIP-00053960; • 1♂ 1♀; L33, 23 Jul 2018, X. Mengual leg.; ZFMK-DIP-00053945, ZFMK-DIP-00053946, ZFMK-DIP-00053947, ZFMK-DIP-00054156 = ZFMK-TIS-8000984; • 2♂ 1♀; L34, 23 Jul 2018, J. and B. Thormann leg.; ZFMK-DIP-00054147 = ZFMK-TIS-8004304, ZFMK-DIP-00054152 = ZFMK-TIS-8004306, ZFMK-DIP-00054145 = ZFMK-TIS-8004307; • 2♂ 3♀; L34, 22 Jul 2018, X. Mengual leg.; ZFMK-DIP-00053944, ZFMK-DIP-00053955, ZFMK-DIP-00053957, ZFMK-DIP-00053958, ZFMK-DIP-00054148 = ZFMK-TIS-8000990; • 1♂; L35, 24 Jul 2018, X. Mengual leg.; ZFMK-DIP-00053948; • 1♂ 2♀; L37, 25 Jul 2018, J. Thormann leg.; ZFMK-DIP-00054149 = ZFMK-TIS-8004079, ZFMK-DIP-00054150 = ZFMK-TIS-8004078 ZFMK-DIP-00054155 = ZFMK-TIS-8004076; • 1♀; L38, 25 Jul 2018, X. Mengual leg.; ZFMK-DIP-00053956; • 1♂; L39, 23–26 Jul 2018, X. Mengual, M. Espeland, B. Thormann leg.; ZFMK-DIP-00054157; • 1♀; L40, 18 Jul 2018, B. Thormann leg.; ZFMK-DIP-00054146 = ZFMK-TIS-8003799; • 1♀; L42, 25 Jul 2018, B. Rulik leg.; MTD-Dip-A-R-4548; • 6♂; L70, 30 Jun–14 Jul 2018, malaise trap, GGBC-members leg.; ZFMK-TIS-8002717, ZFMK-TIS-8002718, ZFMK-DIP-00061296, ZFMK-DIP-00061297, ZFMK-DIP-00061298, ZFMK-DIP-00061299; • 6♂; L70, 30 Jun–14 Jul 2018, malaise trap, GGBC-members leg.; MTD-Dip-A-R-4552; • 7♂ 2♀; L71, 30 Jun–14 Jul 2018, malaise trap, GGBC-members leg.; ZFMK-TIS-8002748, ZFMK-TIS-8002749, ZFMK-DIP-00061300, ZFMK-DIP-00061301, ZFMK-DIP-00061302, ZFMK-DIP-00061303, ZFMK-DIP-00061304, ZFMK-TIS-8002751, ZFMK-TIS-8002750; • 5♂; L71, 30 Jun–14 Jul 2018, malaise trap, GGBC-members leg.; MTD-Dip-A-R-4576; • 3♂ 3♀; L72, 29 Jun–13 Jul 2018, malaise trap, GGBC-members leg.; ZFMK-TIS-8002780, ZFMK-DIP-00061307, ZFMK-DIP-00061308, ZFMK-TIS-8002782, ZFMK-DIP-00061305, ZFMK-DIP-00061306; • 2♂; L72, 29 Jun–13 Jul 2018, malaise trap, GGBC-members leg.; MTD-Dip-A-R-4558.

**Genetics.** We sequenced nine specimens (MN621992, MN621993, MN621994, MN621995, MN621996, MN621997, MN621998, MN621999, MN622000). They differ between 0% and 1.37% among them. These intraspecific distances overlap with interspecific distance; for example, our sequences of *M. mellinum* differ from 0% to 1.22% from sequences of *M. scalare*.

**Distribution.** Palaearctic, eastern Afrotropics, and Indomalayan Region.

**Remarks.**Speight (2018a) cited this species “throughout the Oriental region to New Guinea”, but Ramage et al. (2018) did not report it from Australasian and Oceanian Regions. Haarto and Ståhls (2014) proved that different *Melanostoma* species share the same COI haplotypes among them and that this mitochondrial gene is not very useful for species identification.

***Meligramma guttata* (Fallén, 1817)**

**Reference.**Peck (1988) as Melangyna (Meligramma) guttata (Fallén, 1817); Gudjabidze (2002) as *Syrphus gutatus* (Fallen, 1817) [sic].

**New records.** GEORGIA • 1♂ 1♀; L19, 29 Jun 2018, S. Bot leg.

Distributoin. Holarctic.

***Meliscaeva auricollis* (Meigen, 1822)**

**Reference.**Tóth (1986); Peck (1988); Gudjabidze (2002) as *Syrphus auricollis* Meigen, 1822; Barkalov and Mutin (2018).

**New records.** GEORGIA • 1♀; L2, 16 Jun 2018, S. Bot leg.; • 2♂; L3, 17 Jun 2018, S. Bot leg.; • 1♂; L8, 20 Jun 2018, S. Bot leg.; • 1♂ 1♀; L10, 22 Jun 2018, S. Bot leg.; • 1♀; L12, 24 Jun 2018, S. Bot leg.; • 1♂; L16, 27 Jun 2018, S. Bot leg.; • 1♂; L20, 30 Jun 2018, S. Bot leg.; • 1♀; L35, 24 Jul 2018, X. Mengual leg.; ZFMK-DIP-00054017; • 1♀; L37, 25 Jul 2018, X. Mengual leg.; ZFMK-DIP-00053811; • 1♀; L38, 25 Jul 2018, X. Mengual leg.; ZFMK-DIP-00053810 = ZFMK-TIS-8005580; • 1♀; L38, 25 Jul 2018, X. Mengual leg.; ZFMK-DIP-00053812 = ZFMK-TIS-8005588.

**Genetics.** The two sequenced specimens (MN622001, MN622002) differ on 0.3% in the COI barcode. The BIN for these specimens is BOLD:AAZ5262, with a maximum uncorrected pairwise distance f 2.08% within the BIN.

**Distribution.** Western Palaearctic, including Canary Isles.

***Meliscaeva cinctella* (Zetterstedt, 1843)**

**Reference.**Levitin (1962) as *Syrphus cinctellus* Zett.; Peck (1988); Gudjabidze (2002) as *Syrphus cinctellus* Zetterstendt, 1843 [sic].

**New records.** GEORGIA • 1♀; L31, 23 Jul 2018, A. Reimann leg.; MTD-Dip-A-R-4513.

**Distribution.** Holarctic.

**Merodon (Merodon) aberrans Egger, 1860**

**Reference.**Radde (1899); Peck (1988); Gudjabidze (2002) as *Merodon abarrans* Egger [sic]; Barkalov and Mutin (2018).

**New records.** GEORGIA • 1♂; L7, 19 Jun 2018, S. Bot leg.

**Distribution.** Western Palaearctic.

**Merodon (Merodon) albifrons Meigen, 1822**

**Reference.**Peck (1988) listed it only from Azerbaijan; Gudjabidze (2002); Barkalov and Mutin (2018).

**New records.** GEORGIA • 1♂; L51, 24 Jul 2001, J.-H. Stuke leg.; ZFMK-DIP-00058256.

**Distribution.** Central and Southern Europe, northern Africa, Crimea, and Transcaucasia.

**Merodon (Merodon) annulatus (Fabricius, 1794)**

**Reference.**Peck (1988).

**Remarks.** This species was described from France but it has never been recorded again from this country. Other records were reported from Italy, Greece, and Israel (Speight 2018a) but some of these records need confirmation (Vujić et al. 2020). The last identification key where this species was included was done by Sack (1928–1932). This species needs a redefinition/redescription to help distinguish it from other *Merodon* species (Speight 2018a).

**Merodon (Merodon) aureus Fabricius, 1805**

**Reference.**Radde (1899) as *Merodon aeneus* Meigen, 1822; Peck (1988) as *Merodon aeneus* Meigen, 1822 from Armenia, and as *Merodon aureus* Fabricius, 1805 from Germany and Yugoslavia; Gudjabidze (2002) as *Merodon aeneus* Meigen, 1822.

**Distribution.** Europe, Transcaucasia, and North Africa, but needs reassessment.

**Remarks.** The *Merodon aureus* group comprises a number of different subgroups and species complexes (Veselić et al. 2017). All the identifications of this species are in need of verification to avoid confusion with other species of this complex. We follow Thompson (2019) and consider *Merodon aeneus* Megerle in Meigen, 1822 a junior synonym of *Merodon aureus*.

**Merodon (Merodon) avidus (Rossi, 1790)**

**Reference.**Peck (1988) listed it only from Armenia as *Merodon avidus*, but also listed it as *Merodon spinipes* (Fabricius, 1794); Gudjabidze (2002) as *Merodon spinipes*.

**Distribution.** Mediterranean Basin.

**Remarks.***Merodon avidus* is a species complex with taxonomic difficulties and a considerable morphological variability (Milankov et al. 2001, 2009; Ståhls et al. 2009; Popović et al. 2015; Ačanski et al. 2016). The color variability has been explained by the differential availability of trophic resources during the larval stage (Hurkmans 1993), but difficulties in distinguishing the species of this complex based on morphological characters remain. All the identifications of the species of this complex need verification.

**Merodon (Merodon) caucasicus Portschinsky, 1877**

**Reference.**Portschinsky (1877); Paramonov (1926b) as *Merodon batumicus* Paramonov, 1926; Levitin (1962) as *Lampetia caucasica* Porth.; Peck (1988) as *Merodon batumicus* Paramonov, 1926 and also as *Merodon caucasicus* Portschinsky, 1877; Gudjabidze (2002) as *Merodon batumicus* Paramonov, 1925 [sic] and also as *Merodon caucasicus* Potschinkyi, 1881 [sic]; Barkalov and Mutin (2018).

**New records.** GEORGIA • 2♂ 2♀; L33, 22 Jul 2018, X. Mengual leg.; ZFMK-DIP-00053917 = ZFMK-TIS-8005523, ZFMK-DIP-00053996 = ZFMK-TIS-8003434, ZFMK-DIP-00053918 = ZFMK-TIS-8005529, ZFMK-DIP-00053919 = ZFMK-TIS-8005534; • 2♀; L35, 24 Jul 2018, X. Mengual leg.; ZFMK-DIP-00053912 = ZFMK-TIS-8005514, ZFMK-DIP-00053997 = ZFMK-TIS-8003449; • 9♂ 1♀; L50, 4 Aug 2001, J.-H. Stuke leg.; ZFMK-DIP-00057975, ZFMK-DIP-00057976, ZFMK-DIP-00057977, ZFMK-DIP-00057978, ZFMK-DIP-00057979, ZFMK-DIP-00057980, ZFMK-DIP-00057981, ZFMK-DIP-00057982, ZFMK-DIP-00057983, ZFMK-DIP-00057984; • 2♀; L72, 29 Jun–13 Jul 2018, malaise trap, GGBC-members leg.; MTD-Dip-A-R-4560, ZFMK-TIS-8002768.

**Genetics.** We sequenced five specimens (MN622003, MN622004, MN622005, MN622006, MN622007) that differ from 0% to 0.61% in their COI sequence. This species is not present in BOLD or GenBank, so this are the first COI sequences for this taxon. The closest COI sequence in BOLD systems to the *Merodon caucasicus* sequences is one of *Merodon mariae* Hurkmans, 1993 (3.21–3.82% difference).

**Distribution.** Balkan Peninsula and Transcaucasia.

**Remarks.***Merodon batumicus* Paramonov, 1926 is now considered a junior synonym of *M. caucasicus* (proposed by Popov 2007; A. Vujić pers. comm. in Smit and Langeveld 2018). *Merodon batumicus* was described from Batumi area in Georgia (Paramonov 1926b) and reported for this country by Peck (1988) and Gudjabidze (2002).

**Merodon (Merodon) cinereus (Fabricius, 1794)**

**Reference.**Peck (1988); Gudjabidze (2002) as *Merodon cinereus* Fabricius, 1777 [sic].

**New records.** GEORGIA • 1♂; L15, 26 Jun 2018, S. Bot leg.

**Distribution.** Needs reassessment.

**Remarks.***Merodon cinereus* is a species complex (Milankov et al. 2008; Francuski et al. 2011; Šašić et al. 2016) and all the identifications of this species complex are in need of verification.

**Merodon (Merodon) crassifemoris Paramonov, 1925**

**Reference.**Barkalov and Mutin (2018).

**Distribution.** Mediterranean Basin, Crimea, and Transcaucasia.

**Remarks.**Barkalov and Mutin (2018) listed this species from Transcaucasia, but Speight (2018a) listed it only from Azerbaijan.

**Merodon (Merodon) femoratus Sack, 1913**

**Reference.**Peck (1988); Gudjabidze (2002) as *Merodon femoralis* Sack, 1932 [sic].

**Distribution.** Mediterranean Basin, Crimea and Transcaucasia.

**Merodon (Merodon) gudaurensis Portschinsky, 1877**

**Reference.**Portschinsky (1877); Peck (1988); Gudjabidze (2002) as *Merodon gudauriensis* Potshinskyi, 1881 [sic].

**Distribution.** Georgia.

**Merodon (Merodon) kiritshenkoi (Stackelberg, 1960)**

**Reference.**Peck (1988); Barkalov and Mutin (2018).

**Distribution.** Northern Caucasus and Transcaucasia.

**Remarks.** The type locality of this species is in North Ossetia-Alania (Northern Caucasus), but Peck (1988) and Barkalov and Mutin (2018) listed it also from Transcaucasia.

**Merodon (Merodon) loewi Van der Goot, 1964**

**Reference.**Peck (1988) listed it only from Armenia; Gudjabidze (2002); Barkalov and Mutin (2018).

**Distribution.** Europe, southern parts of European Russia, Transcaucasia, Turkey, and Israel.

**Merodon (Merodon) moenium Hoffmannsegg in Meigen, 1822**

**New records.** GEORGIA • 1♂; L4, 18 Jun 2018, S. Bot leg.; • 3♂; L7, 19 Jun 2018, S. Bot leg.; •1♂; L8, 20 Jun 2018, S. Bot leg.; • 4♂; L10, 22 Jun 2018, S. Bot leg.; • 1♀; L50, 4 Aug 2001, J.-H. Stuke leg.; ZFMK-DIP-00057998.

**Remarks.** This species belongs to the *avidus* species complex and the identification using adult morphology is not straightforward (see remarks under *Merodon avidus*). Spring generations of *Merodon avidus* are very similar to those of *M. moenium* (Ačanski et al. 2016). Reported for Georgia for the first time.

**Merodon (Merodon) nanus (Sack, 1931)**

**Reference.**Peck (1988); Gudjabidze (2002) as *Merodon nanus* Sack, 1932 [sic]; Barkalov and Mutin (2018).

**Distribution.** Needs reassessment, but its presence confirmed from Greece, Armenia, Iran, and Middle East.

**Remarks.***Merodon nanus* is a species complex (Vujić et al. 2015; Tubić et al. 2018) and all the records of this species complex are in need of verification.

**Merodon (Merodon) natans (Fabricius, 1794)**

**Reference.**Speight (2018a).

**Distribution.** Mediterranean Basin and Caucasus Mountains.

**Remarks.** Speceis very similar to Merodon (Merodon) pulveris Vujić and Radenković in Radenković et al. 2011 (Radenković et al. 2011).

**Merodon (Merodon) nigritarsis Rondani, 1845**

**Reference.**Peck (1988) as *Merodon spinipes nigritarsis* Rondani, 1845.

**Distribution.** Europe, Transcaucasia, and Turkey, but needs reassessment.

Remarks: *M. nigritarsis* is part of the *nigritarsis* species group. It is unclear which species name should be applied to the specimens mentioned by Peck (1988). All records of this species group are in need of verification.

**Merodon (Merodon) obscuritarsis Strobl in Czerny & Strobl, 1909**

**Reference.**Barkalov and Mutin (2018).

**Distribution.** Needs reassessment, but recorded from Spain and France. Barkalov and Mutin (2018) mentioned also Transcaucasia and northern Africa as part of its range.

**Remarks.**Marcos-García et al. (2007) stated that *M. tricinctus* Sack, 1913 is closely related to *M. obscuritarsis* and can be a synonym, but further studies are needed. Barkalov and Mutin (2018) reported both *M. obscuritarsis* and *Merodon tricinctus* from the Transcaucasia.

**Merodon (Merodon) portschinskyi (Stackelberg, 1924)**

**Reference.**Peck (1988); Gudjabidze (2002) as *Merodon portshinskyi* Sthakelberg, 1956 [sic]; Speight (2018a).

**New records.** GEORGIA • 2♂ 3♀; L4, 18 Jun 2018, S. Bot leg.; • 1♂; L5, 18 Jun 2018, S. Bot leg.; • 1♂ 1♀; L15, 26 Jun 2018, S. Bot leg.; • 1♂; L19, 29 Jun 2018, S. Bot leg.; • 6♂; L20, 1 Jul 2018, S. Bot leg.; • 4♂; L20, 2 Jul 2018, S. Bot leg.

**Distribution.** Northern Caucasus and Transcaucasia.

**Merodon (Merodon) pruni (Rossi, 1790)**

**Reference.**Peck (1988).

**Distribution.** Western Palaearctic, including Turkmenistan and Iraq.

**Merodon (Merodon) ruficornis Meigen, 1822**

**Reference.**Peck (1988); Gudjabidze (2002); Barkalov and Mutin (2018); Speight (2018a).

**Distribution.** Cetral and Southern Europe, Balkan Peninsula and Ukraine (see Vujić et al. 2012).

**Remarks.***M. ruficornis* is part of a species complex. Several species of this complex occur or are likely to occur in Georgia, but *M. ruficornis* itself is only known from Europe (Vujić et al. 2012). All the records of this species are in need of verification.

**Merodon (Merodon) rufipes Sack, 1913**

**Reference.**Gudjabidze (2002) as *Merodon rufipes* Sack, 1932 [sic].

**Distribution.** Bulgaria, Ukraine, and Georgia.

**Merodon (Merodon) tricinctus Sack, 1913**

**Reference.**Peck (1988) listed it only from Armenia; Barkalov and Mutin (2018); Speight (2018a).

**Distribution.** Western Palaearctic.

**Remarks.** Marcos-Garcia et al. (2007) stated that *M. tricinctus* is closely related to *M. obscuritarsis* and they can be synonyms, but further studies are needed.

**Merodon (Merodon) velox Loew, 1869**

**Reference.**Peck (1988).

**Distribution.** Balkan Peninsula, Greece, Turkey, and Transcaucasia.

***Mesembrius peregrinus* (Loew, 1846)**

**Reference.**Peck (1988); Gudjabidze (2002) as Helophilus (Mesembriuc) peregrinus Loew, 1846 [sic]; Speight (2018a).

**New records.** GEORGIA • 1♀; L60, 26 Jul 2001, J.-H. Stuke leg.; ZFMK-DIP-00057985.

**Distribution.** Palaearctic.

***Microdon analis* (Macquart, 1842) / *Microdon major* Andries, 1912**

**Reference.**Levitin (1962) as *Microdon eggeri* Mik.; Peck (1988) as *Microdon eggeri*Mik, 1897; Gudjabidze (2002) as *Microdon eggeri* Mick, 1897 [sic].

**New records.** GEORGIA • 1♂; L16, 26 Jun 2018, S. Bot leg.; •1♀; L70, 30 Jun–14 Jul 2018, malaise trap, GGBC-members leg.; ZFMK-TIS-8002706.

**Genetics.** We sequenced one specimen (MN622008), and its COI barcode is very similar to other published sequences of *M. analis* (99.85% similarity), *M. mutabilis* (98.92% similarity) and *M. major* (96.94% similarity). The BIN for this taxon is BOLD:ABA2554.

**Distribution.** Palaearctic, but needs reassessment for each species of this complex.

**Remarks.**Doczkal and Schmid (1999) synonymised *M. eggeri* under *M. analis*. *Microdon analis* and *Microdon major* can only be distinguished using features of its developmental stages (Schmid 2004). It is unclear if one of the two or both species occur in Georgia.

***Microdon mutabilis* (Linnaeus, 1758) / *Microdon myrmicae*Schönrogge et al., 2002**

**Reference.**Tóth (1986) as *Microdon mutabilis* (Linnaeus, 1758); Peck (1988) as *M. mutabilis*; Gudjabidze (2002) as *M. mutabilis*; Barkalov and Mutin (2018) as *M. mutabilis*.

**New records.** GEORGIA • 1♂; L6, 19 Jun 2018, S. Bot leg.; • 1♂; L12, 24 Jun 2018, S. Bot leg.; • 1♂; L20, 30 Jun 2018, S. Bot leg.; • 1♂; L20, 1 Jul 2018, S. Bot leg.

**Distribution.** Palaearctic, but needs reassessment for each species of this complex.

**Remarks.***Microdon mutabilis* and *Microdon myrmicae* can only be distinguished using features of its developmental stages (Schönrogge et al. 2002). It is unclear if one of the two or both species occur in Georgia.

***Milesia crabroniformis* (Fabricius, 1775)**

**Reference.**Peck (1988); Gudjabidze (2002) as *Milesia crabroniformis* Linnaeus, 1758 [sic].

**New records.** GEORGIA • 3♂ 4♀; L24, 17 Jul 2018, X. Mengual leg.; ZFMK-DIP-00053969 = ZFMK-TIS-8003454, ZFMK-DIP-00053628, ZFMK-DIP-00053629, ZFMK-DIP-00053631, ZFMK-DIP-00053632 = ZFMK-TIS-8005543, ZFMK-DIP-00053633, ZFMK-DIP-00053634; • 1♂; L25, 18 Jul 2018, X. Mengual leg.; ZFMK-DIP-00053630 = ZFMK-TIS-8005535; • 1♀; L37, 25 Jul 2018, X. Mengual leg.; ZFMK-DIP-00053635; • 1♀; L42, 25 Jul 2018, A. Reimann leg.; MTD-Dip-A-R-4531; • 4♂ 1♀; L69, 23 Jul 2018, A. Reimann leg.; MTD-Dip-A-R-4505, MTD-Dip-A-R-4515, MTD-Dip-A-R-4516, ZFMK-GGBC8002669, MTD-Dip-A-R-4517.

**Genetics.** Three specimens were sequenced (MN622009, MN622010, MN622011), and their COI barcodes differ from 0% to 0.38%. BOLD currently lists two specimens with COI sequences from Portugal (99.54–99.85% similarity with our samples), but the data are not public prior to this publication.

**Distribution.** Central and Southern Europe, North Africa, Turkey and Georgia.

**Remarks.**Zaitzev (1912) listed from Abkhazia an unidentified species of *Milesia*, close to *M. cabroniformis* but with larger yellow pattern. The identity of this taxon remains unclear.

***Milesia semiluctifera* (Villers, 1798)**

**Reference.**Peck (1988); Seropian (2013b) as field observation; Speight (2018a).

**Distribution.** Europe, Middle East, Transcaucasia, east into Turkmenistan.

***Myathropa florea* (Linnaeus, 1758)**

**Reference.**Radde (1899) as *Helophilus floreus* (Linnaeus, 1758) and *Helophilus nigrotarsatus* Schiner, 1860; Levitin (1962) as *Myiatropa florea* L. [sic]; Tóth (1986); Peck (1988); Gudjabidze (2002) as *Myiatropa florae* Linnaeus, 1758 [sic].

**New records.** GEORGIA • 1♂; L3, 17 Jun 2018, S. Bot leg.; • 1♀; L8, 20 Jun 2018, S. Bot leg.; • 3♂; L10, 22 Jun 2018, S. Bot leg.; • 2♀; L20, 1 Jul 2018, S. Bot leg.; • 1♂; L21, 3 Jul 2018, S. Bot leg.; • 3♂; L22, 3 Jul 2018, S. Bot leg.; • 3♀; L24, 17 Jul 2018, X. Mengual leg.; ZFMK-DIP-00053746 = ZFMK-TIS-8005563, ZFMK-DIP-00053750 = ZFMK-TIS-8005564, ZFMK-DIP-00054089 = ZFMK-TIS-8000972; • 1♂; L31, 23 Jul 2018, A. Reimann leg.; MTD-Dip-A-R-4509; • 2♀; L31, 23 Jul 2018, A. Reimann leg.; MTD-Dip-A-R-4521; • 1♂; L33, 22 Jul 2018, X. Mengual leg.; ZFMK-DIP-00053748 = ZFMK-TIS-8005556; • 1♀; L35, 24 Jul 2018, J. Thormann leg.; ZFMK-DIP-00054090 = ZFMK-TIS-8003928; • 1♂ 4♀; L37, 25 Jul 2018, X. Mengual leg.; ZFMK-DIP-00053743, ZFMK-DIP-00053745, ZFMK-DIP-00053747, ZFMK-DIP-00053749 = ZFMK-TIS-8005738, ZFMK-DIP-00053994 = ZFMK-TIS-8003423; • 1♂; L38, 25 Jul 2018, B. Thormann leg.; ZFMK-DIP-00054091 = ZFMK-TIS-8004229; • 2♂; L38, 25 Jul 2018, X. Mengual leg.; ZFMK-DIP-00053742 = ZFMK-TIS-8005555, ZFMK-DIP-00053744; • 1♂ 2♀; L42, 25 Jul 2018, A. Reimann leg.; MTD-Dip-A-R-4538; • 1♂; L48, 24 Jul 2001 J.-H. Stuke leg.; ZFMK-DIP-00058001; • 1♀; L49, 4 Aug 2001, J.-H. Stuke leg.; ZFMK-DIP-00058003; • 1♀; L53, 1 Aug 2001, J.-H. Stuke leg.; ZFMK-DIP-00058002; • 1♂; L57, 3 Aug 2001, J.-H. Stuke leg.; ZFMK-DIP-00058255; • 1♂ 1♀; L69, 18 Jul 2018, A. Reimann leg.; MTD-Dip-A-R-4499, ZFMK-TIS-8002663.

**Genetics.** Four specimens were successfully sequenced (MN622012, MN622013, MN622014, MN622015) and their COI barcodes differ 0.15–0.61%. Currently there are three BINs in BOLD systems with specimens identified as *M. florea*: BOLD:ADQ8445, BOLD:ADR1776, and BOLD:AAP9713. Our specimens belong to the last BIN.

**Distribution.** Palaearctic.

***Myolepta dubia* (Fabricius, 1805)**

**Reference.**Peck (1988) as *Myolepta luteola* (Gmelin, 1790); Gudjabidze (2002) as *M. luteola*; Reemer et al. (2005); Barkalov and Mutin (2018).

**Distribution.** Europe, European parts of Russia and Transcaucasia.

**Remarks.**Thompson and Pont (1994) noted that the name *Musca luteola* Gmelin, 1790 was preoccupied by *Musca luteola* Scopoli, 1763 and suggested the name *Thereva dubia* Fabricius, 1805 [= *Myolpeta dubia* (Fabricius, 1805)] as the next available name for this taxon.

Reemer et al. (2005) mentioned the possibility that part of the records of *M. dubia* from Transcaucasia (Stackelberg and Richter 1968 from Azerbaijan) may belong to *Myolepta trojana* Reemer and Hauser in Reemer, Hauser & Speight, 2005, as only one single specimen from Georgia was identified as *M. dubia*.

***Myolepta nigritarsis* Coe, 1957**

**Reference.**Peck (1988).

**Distribution.** Europe, European parts of Russia, and Transcaucasia.

***Myolepta obscura* Becher, 1882**

**Reference.**Peck (1988).

**Distribution.** Central Europe, Balkan Peninsula, Turkey, and Transcaucasia.

***Myolepta potens* (Harris, 1779)**

**Reference.**Peck (1988); Speight (2018a).

**Distribution.** Europe, Turkey, and Transcaucasia.

**Remarks.**Reemer et al. (2005) suggested caution with records of this species from the Caucasus Region (see Stackelberg and Richter 1968) because the closely related species *Myolepta mada* Reemer and Hauser in Reemer, Hauser & Speight, 2005 may occur in this region, although it is currently known only from Azerbaijan.

The year of publication for this species is a convention. The original work by Harris (1776–1780) was published in five ‘decads’ or parts. Peck (1988) used the conventional date of 1780? with a question mark for decads 3, 4, and 5 based on Lisney (1960). Evenhuis (1997: page 343) established that the decad 4, where *Musca potens* is described on page 110, was dated as 1779 based on the latest date of the plates. Thus, the year of publication should be 1779.

***Myolepta trojana* Reemer and Hauser in Reemer, Hauser & Speight, 2005**

**New records.** GEORGIA • 2♂; L21, 3 Jul 2018, S. Bot leg.

**Distribution.** Greece, Turkey, Azerbaijan, and Iran.

**Remarks.** Reported for Georgia for the first time.

***Myolepta vara* (Panzer, 1798)**

**Reference.**Peck (1988).

**Distribution.** Europe and Transcaucasia.

**Remarks.**Reemer et al. (2005) studied material from Azerbaijan, but no published record exists explicitly from Georgia. Peck (1988) listed the Far East (Khabarovsk and Primorye Territories) in the range of this species, but these records needs verification (Reemer et al. 2005).

**Neoascia (Neoascia) annexa (Müller, 1776)**

**Reference.**Peck (1988) as *Neoascia floralis* (Meigen, 1822); Gudjabidze (2002) as *Neoascia floralis* Meigen, 1822 [sic]; Speight (2018a).

**New records.** GEORGIA • 1♀; L65, 23 Jul 2001, J.-H. Stuke leg.; ZFMK-DIP-00057973.

**Distribution.** Europe, European parts of Russia, and Transcaucasia.

**Remarks.** Thompson (1981) synonymised *Ascia floralis* Meigen, 1822 [= *Neoascia floralis* (Meigen, 1822)] under *Neoascia podagrica* (Fabricius, 1775), and explained that the name *floralis* Meigen was applied wrongly by some authors to *N. annexa*. Previous records of *N. annexa* need verification as they might belong to *Neoascia subannexa* Claußen and Hayat 1997.

**Neoascia (Neoascia) podagrica (Fabricius, 1775)**

**Reference.**Tóth (1986); Peck (1988); Gudjabidze (2002) as *Neoascia podagrica* Fabricius, 1794 [sic]; Barkalov and Mutin (2018).

**Distribution.** Palaearctic.

**Neoascia (Neoascia) tenur (Harris, 1779)**

**Reference.**Peck (1988) as *Neoascia dispar* (Meigen, 1822).

**New records.** GEORGIA • L52, 30 Jul 2001, J.-H. Stuke leg.; 1♀; ZFMK-DIP-00057974].

**Distribution.** Europe, European parts of Russia, Turkey, Transcaucasia, and into Siberia.

**Remarks.** Thompson (1981) synonymised *Ascia dispar* Meigen, 1822 [= *Neoascia dispar* (Meigen, 1822)] under *Neoascia meticulosa* (Scopoli, 1763), and explained that the name *dispar* Meigen was applied wrongly by some authors to *N. tenur*.

The year of publication for this species is a convention. The original work by Harris (1776–1780) was published in five ‘decads’ or parts. Peck (1988) used the conventional date of 1780? with a question mark for decads 3, 4, and 5 based on Lisney (1960). Evenhuis (1997: page 343) established that the decad 4, where *Musca tenur* is described on page 112, was dated as 1779 based on the latest date of the plates. Thus, the year of publication should be 1779.

**Neoascia (Neoascia) subannexa Claußen and Hayat 1997**

**New records.** GEORGIA • 1♂; L3, 17 Jun 2018, S. Bot leg.; • 1♂; L10, 22 Jun 2018, S. Bot leg.; • 1♀; L19, 29 Jun 2018, S. Bot leg.; • 1♀; L20, 1 Jul 2018, S. Bot leg.; • 1♀; L24, 17 Jul 2018, X. Mengual leg.; ZFMK-DIP-00053862; • 6♂1♀; L25, 18 Jul 2018, X. Mengual leg.; ZFMK-DIP-00053855, ZFMK-DIP-00053858, ZFMK-DIP-00053859 = ZFMK-TIS-8005596, ZFMK-DIP-00053860, ZFMK-DIP-00054092, ZFMK-DIP-00054094, ZFMK-DIP-00053864; • 2♂ 3♀; L26, 18 Jul 2018, X. Mengual leg.; ZFMK-DIP-00053856, ZFMK-DIP-00053857, ZFMK-DIP-00054093, ZFMK-DIP-00054095, ZFMK-DIP-00053861; • 1♀; L30, 19 Jul 2018, X. Mengual leg.; ZFMK-DIP-00053863 = ZFMK-TIS-8005602; • 1♀; L58, 27 Jul 2001, J.-H. Stuke leg.; ZFMK-DIP-00057972; • 2♂; L31, 23 Jul 2018, A. Reimann leg.; MTD-Dip-A-R-4529, ZFMK-GGBC8002675; • 1♂; L69, 18 Jul 2018, A. Reimann leg.; MTD-Dip-A-R-4505; • 2♀; L70, 30 Jun–14 Jul 2018, malaise trap, GGBC-members leg.; ZFMK-TIS-8002724, MTD-Dip-A-R-4596; • 4♂ 1♀; L71, 30 Jun–14 Jul 2018, malaise trap, GGBC-members leg.; ZFMK-TIS-8002761, ZFMK-TIS-8002762, ZFMK-TIS-8002763, ZFMK-DIP-00061309, ZFMK-DIP-00061312; • 3♂ 1♀; L71, 30 Jun–14 Jul 2018, malaise trap, GGBC-members leg.; MTD-Dip-A-R-4557, MTD-Dip-A-R-4580; • 1♂ 2♀; L72, 29 Jun–13 Jul 2018, malaise trap, GGBC-members leg.; ZFMK-TIS-8002786, ZFMK-DIP-00061310, ZFMK-DIP-00061311.

**Genetics.** We sequenced seven specimens (MN622016, MN622017, MN622018, MN622019, MN622020, MN622021, MN622022), whose COI barcodes differ 0–1.52%. This species was not yet registered in BOLD, and our COI sequences were similar (> 98.3%) to other private sequences in BOLD identified as *Neoascia annexa*.

**Distribution.** Turkey and Georgia.

**Remarks.** Reported for Georgia for the first time.

**Neoascia (Neoasciella) geniculata (Meigen, 1822)**

**Reference.**Gudjabidze (2002).

**Distribution.** Europe, into Russia to eastern Siberia.

**Neoascia (Neoasciella) interrupta (Megerle in Meigen, 1822)**

**Reference.**Peck (1988); Gudjabidze (2002); Speight (2018a).

**Distribution.** Europe into Russia to eastern Siberia, and Caucasus Region.

**Neoascia (Neoasciella) meticulosa (Scopoli, 1763)**

**Reference.**Peck (1988) as *Neoascia aenea* Meigen, 1822; Gudjabidze (2002) as *Neoascia aenea* Meigen, 1822; Speight (2018a).

**Distribution.** Palaearctic.

**Remarks.** Thompson (1981) synonymised *N. aenea* under *N. meticulosa*.

**Neoascia (Neoasciella) obliqua Coe, 1940**

**Reference.**Peck (1988) from Armenia; Gudjabidze (2002) as *Neoascia oblique* Coe [sic]; Speight (2018a).

**Distribution.** Europe, European parts of Russia, and Transcaucasia.

***Neocnemodon latitarsis* (Egger, 1865)**

**Reference.**Peck (1988); Gudjabidze (2002) as *Cnemodon latitarsis* Egger, 1776 [sic]; Barkalov and Mutin (2018); Speight (2018a).

**Distribution.** Europe, European parts of Russia, and Transcaucasia. Recorded in North America (New Brunswick) but not established (Skevington et al. 2019).

***Neocnemodon vitripennis* (Meigen, 1822)**

**Reference.**Peck (1988).

**Distribution.** Palaearctic.

***Orthonevra brevicornis* (Loew, 1843)**

**Reference.**Peck (1988); Gudjabidze (2002) as *Orthoneura brevicornis* Loew, 1848 [sic] and *Chrysogaster brevicornis* Loew, 1848 [sic]; Speight (2018a).

**Distribution.** Europe, Transcaucasia, eastwards into Siberia.

***Orthonevra elegans* (Wiedemann in Meigen, 1822)**

**Reference.**Peck (1988); Gudjabidze (2002).

**Distribution.** Palaearctic, except in the Mediterranean Basin.

***Orthonevra frontalis* (Loew, 1843)**

**Reference.**Peck (1988).

**Distribution.** Palaearctic.

***Orthonevra intermedia* Lundbeck, 1916**

**Reference.**Gudjabidze (2002) as *Chrysogaster intermedia* Lennaeus, 1758 [sic].

**Distribution.** Palaearctic.

***Orthonevra nobilis* (Fallén, 1817)**

**Reference.**Peck (1988); Gudjabidze (2002); Speight (2018a).

**New records.** GEORGIA • 1♀; L11, 29 Jun 2018, S. Bot leg.; • 3♀; L53, 1 Aug 2001, J.-H. Stuke leg.; ZFMK-DIP-00057994, ZFMK-DIP-00057995, ZFMK-DIP-00057996; • 1♀; L57, 3 Aug 2001, J.-H. Stuke leg.; ZFMK-DIP-00057997.

**Distribution.** Palaearctic.

***Orthonevra pilifacies* Stackelberg, 1952**

**Reference.**Peck (1988).

**Distribution.** Transcaucasia and Central Palaearctic into Afghanistan.

***Orthonevra plumbago* (Loew, 1840)**

**Reference.**Gudjabidze (2002) as *Orthonevra plumbago* Loew, 1848 [sic].

**Distribution.** Europe, European parts of Russia, and Georgia.

**Paragus (Pandasyophthalmus) constrictus Šimič, 1986**

**New records.** GEORGIA • 1♂; L10, 22 Jun 2018, S. Bot leg.; • 1♂; L49, 4 Aug 2001, J.-H. Stuke leg.; ZFMK-DIP-00057880; • 3♂; L59, 28 Jul 2001, J.-H. Stuke leg.; ZFMK-DIP-00057877, ZFMK-DIP-00057878, ZFMK-DIP-00057879.

**Distribution.** Palaearctic, but needs reassessment due to confusion with other similar species.

**Remarks.** Reported for Georgia for the first time.

**Paragus (Pandasyopthalmus) haemorrhous Megerle in Meigen, 1822**

**Reference.**Tóth (1986); Peck (1988).

**New records.** GEORGIA • 1♂; L4, 18 Jun 2018, S. Bot leg.; • 1♂; L10, 22 Jun 2018, S. Bot leg.; • 1♂; L20, 1 Jul 2018, S. Bot leg.; • 1♂; L31, 21 Jul 2018, J. Astrin leg.; ZFMK-TIS-8000135; • 1♂; L37, 25 Jul 2018, X. Mengual leg.; ZFMK-DIP-00054198; • 4♂ 1♀; L39, 23–26 Jul 2018, malaise trap, X. Mengual, M. Espeland, B. Thormann leg.; ZFMK-DIP-00054196 = ZFMK-TIS-8000869, ZFMK-DIP-00054199, ZFMK-DIP-00054200, ZFMK-DIP-00054201, ZFMK-DIP-00054202; • 1♂; L49, 4 Aug 2001, J.-H. Stuke leg.; ZFMK-DIP-00057856; • 1♂; L60 26 Jul 2001, J.-H. Stuke leg.; ZFMK-DIP-00057853; • 19♂; L66, 28 Jul 2001, J.-H. Stuke leg.; ZFMK-DIP-00057834, ZFMK-DIP-00057835, ZFMK-DIP-00057836, ZFMK-DIP-00057837, ZFMK-DIP-00057838, ZFMK-DIP-00057839, ZFMK-DIP-00057840, ZFMK-DIP-00057841, ZFMK-DIP-00057842, ZFMK-DIP-00057843, ZFMK-DIP-00057844, ZFMK-DIP-00057845, ZFMK-DIP-00057846, ZFMK-DIP-00057847, ZFMK-DIP-00057848, ZFMK-DIP-00057849, ZFMK-DIP-00057850, ZFMK-DIP-00057851, ZFMK-DIP-00057852; • 2♂; L68, 29 Jul 2001, J.-H. Stuke leg.; ZFMK-DIP-00057854, ZFMK-DIP-00057855; • 1♂; L72, 29 Jun–13 Jul 2018, malaise trap, GGBC-members leg.; ZFMK-TIS-8002789.

**Genetics.** We successfully sequenced three specimens (MN622025, MN622026, MN622032) and their COI barcodes differ from 0.3% to 0.61%. In BOLD there are three BINs with specimens identified as *P. haemorrhous*: BOLD:AAC2439, BOLD:ABZ4619, and BOLD:AAC2438.

**Distribution.** Holarctic and Afrotropical Regions.

**Paragus (Pandasyopthalmus) tibialis (Fallén, 1817)**

**Reference.**Bigot (1880) as *Orthonevra varipes* Bigot, 1880; Levitin (1962); Gudjabidze (2002).

**New records.** GEORGIA • 1sp; L31, 21 Jul 2018, J. Astrin leg.; ZFMK-TIS-8000135; • 1♂; L45, 25 Jul 2001, J.-H. Stuke leg.; ZFMK-DIP-00057868; • 1♂; L46, 24 Jul 2001, J.-H. Stuke leg.; ZFMK-DIP-00057860; • 2♂; L59, 28 Jul 2001 J.-H. Stuke leg.; ZFMK-DIP-00057859, ZFMK-DIP-00057869; • 8♂; L63, 23 Jul 2001, J.-H. Stuke leg.; ZFMK-DIP-00057858, ZFMK-DIP-00057861, ZFMK-DIP-00057862, ZFMK-DIP-00057863, ZFMK-DIP-00057864, ZFMK-DIP-00057865, ZFMK-DIP-00057866, ZFMK-DIP-00057867; • 1♂; L68, 29 Jul 2001, J.-H. Stuke leg.; ZFMK-DIP-00057857; • 1♀; L71, 30 Jun–14 Jul 2018, malaise trap, GGBC-members leg.; ZFMK-TIS-8002759.

**Genetics.** One specimen of this species was sequenced (MN622023), which has identical COI barcode as other specimens of *P. tibialis* previously published (AY174468, AY174465, AY476841 from the BIN BOLD:ABZ4619), but also has 100% similarity in the COI sequence with specimens of *P. coadunatus* Rondani, 1847 (AY174467) and *P. haemorrhous* (AY174470, AY174466, AY174469).

**Distribution.** Western Palaearctic, but needs reassessment.

**Paragus (Paragus) albifrons (Fallén, 1817)**

**Reference.**Peck (1988); Gudjabidze (2002); Speight (2018a).

**New records.** GEORGIA • 1♂; L47, 25 Jul 2001, J.-H. Stuke leg.; ZFMK-DIP-00057872; • 1♂; L58, 27 Jul 2001, J.-H. Stuke leg.; ZFMK-DIP-00057870; • 1♂; L68, 29 Jul 2001, J.-H. Stuke leg.; ZFMK-DIP-00057871.

**Distribution.** Palaearctic.

**Paragus (Paragus) bicolor (Fabricius, 1794)**

**Reference.**Peck (1988); Gudjabidze (2002).

**New records.** GEORGIA • 1♀; L32, 22 Jul 2018, X. Mengual leg.; ZFMK-DIP-00053870 = ZFMK-TIS-8005600; • 1♂; L47, 25 Jul 2001, J.-H. Stuke leg.; ZFMK-DIP-00057875; • 1♂; L49, 4 Aug 2001, J.-H. Stuke leg.; ZFMK-DIP-00057876; • 1♂; L63, 23 Jul 2001, J.-H. Stuke leg.; ZFMK-DIP-00057874.

**Genetics.** A single specimen was sequenced (MN622024), and its COI barcode is identical as one published sequence for a specimen identified as *Paragus testaceus* Meigen, 1822 (AY476848) and 99.42% similar to another specimen of *P. bicolor* (AY174462), or 99.83% similar to two private sequences of *P. bicolor*. The BIN BOLD:AAF8068 in BOLD systems comprises specimens of *P. testaceus* and *P. bicolor*.

**Distribution.** Holarctic.

**Paragus (Paragus) compeditus Wiedemann, 1830**

**Reference.**Peck (1988).

**Distribution.** Palaearctic and Afrotropical Regions.

**Paragus (Paragus) finitimus Goeldlin de Tiefenau, 1971**

**Reference.**Tóth (1986).

**Distribution.** Palaearctic.

**Paragus (Paragus) flammeus Goeldlin de Tiefenau, 1971**

**Reference.**Speight (2018a).

**Distribution.** Western and Central Palaearctic.

**Paragus (Paragus) kopdagensis Hayat & Claußen, 1997**

**Reference.**Speight (2018a) from Northern Caucasus.

**New records.** GEORGIA • 2♂; L57, 3 Aug 2001, J.-H. Stuke leg.; ZFMK-DIP-00057832, ZFMK-DIP-00057833.

**Distribution.** Turkey and Caucasus Region.

**Remarks.** Reported for Georgia for the first time.

**Paragus (Paragus) pecchiolii Rondani, 1857**

**New records.** GEORGIA • 1♂; L24, 17 Jul 2018, X. Mengual leg.; ZFMK-DIP-00054205 = ZFMK-TIS-8000969; • 1♀; L34, 22 Jul 2018, X. Mengual leg.; ZFMK-DIP-00054206 = ZFMK-TIS-8000989; • 1♂ 1♀; L39, 23–26 Jul 2018, malaise trap, X. Mengual, M. Espeland, B. Thormann leg.; ZFMK-DIP-00054203 = ZFMK-TIS-8000871, ZFMK-DIP-00054204 = ZFMK-TIS-8000872; • 2♂ 1♀; L72, 29 Jun–13 Jul 2018, malaise trap, GGBC-members leg.; ZFMK-TIS-8002787, ZFMK-DIP-00061313, ZFMK-TIS-8002788; • 2♂; L72, 29 Jun–13 Jul 2018, malaise trap, GGBC-members leg.; MTD-Dip-A-R-4562.

**Genetics.** We sequenced four specimens (MN622027, MN622028, MN622029, MN622030) and their COI barcodes differ from 0% to 0.15%, and they are > 99.2% similar to other published and non-publicly available COI sequences of *P. pecchiollii*. The BIN for our specimens is BOLD:ABA3664.

**Distribution.** Western Palaearctic.

**Remarks.** Reported for Georgia for the first time.

**Paragus (Paragus) quadrifasciatus Meigen, 1822**

**Reference.**Peck (1988); Gudjabidze (2002); Speight (2018a).

**New records.** GEORGIA • 1♀; L39, 23–26 Jul 2018, malaise trap, X. Mengual, M. Espeland, B. Thormann leg.; ZFMK-DIP-00054195 = ZFMK-TIS-8000868; • 1♂; L61, 22 Jul 2001, J.-H. Stuke leg.; ZFMK-DIP-00057882.

**Genetics.** A single female was sequenced (MN622031) and its COI barcode is very similar to other private sequences of *P. quadrifasciatus* in BOLD systems (> 99.7%). The BIN for our specimen is BOLD:ACG5063.

**Distribution.** Palaearctic.

***Parasyrphus annulatus* (Zetterstedt, 1838)**

**Reference.**Peck (1988); Gudjabidze (2002) as *Syrphus annulatus* Zetterstend, 1843 [sic]; Speight (2018a).

**New records.** GEORGIA • 1♀; L3, 17 Jun 2018, S. Bot leg.; • 1♂ 1♀; L7, 19 Jun 2018, S. Bot leg.

**Distribution.** Palaearctic.

***Parasyrphus nigritarsis* (Zetterstedt, 1843)**

**Reference.** Peck (1988)

**New records.** GEORGIA • 5♂; L17, 28 Jun 2018, S. Bot leg.

**Distribution.** Holarctic.

***Parasyrphus punctulatus* (Verrall, 1873)**

**Reference.**Tóth (1986); Peck (1988); Gudjabidze (2002) as *Syrphus punctullatus* Verall [sic]; Speight (2018a).

**Distribution.** Palaearctic, including Nepal.

***Parasyrphus vittiger* (Zetterstedt, 1843)**

**Reference.**Peck (1988); Speight (2018a).

**Distribution.** Europe, European parts of Russia into Siberia, and Caucasus Region.

***Parhelophilus frutetorum* (Fabricius, 1775)**

**Reference.**Peck (1988) as Helophilus (Parhelophilus) frutetorum (Fabricius, 1775); Speight (2018a).

**Distribution.** Europe, European parts of Russia into Siberia, and Transcaucasia.

***Parhelophilus versicolor* (Fabricius, 1794)**

**Reference.**Peck (1988)Helophilus (Parhelophilus) versicolor (Fabricius, 1794).

**Distribution.** Palaearctic.

**Pelecocera (Chamaesyrphus) scaevoides (Fallén, 1817)**

**Reference.**Peck (1988) as *Chamaesyrphus scaevoides* (Fallén, 1817); Gudjabidze (2002) as *Chamaesyrphus scaevoides* (Fallén, 1817); Speight (2018a).

**Distribution.** Europe, European parts of Russia, and Transcaucasia.

**Pelecocera (Pelecocera) tricincta Hoffmannsegg in Meigen, 1822**

**Reference.**Peck (1988); Speight (2018a).

**Distribution.** Europe, Transcaucasia, European parts of Russia into Siberia.

***Pipiza austriaca* Meigen, 1822**

**Reference.**Peck (1988).

**Distribution.** Needs reassessment after Vujić et al. (2013).

***Pipiza festiva* Meigen, 1822**

**Reference.**Peck (1988); Gudjabidze (2002); Barkalov and Mutin (2018); Speight (2018a).

**Distribution.** Palaearctic, but not in northern Africa.

***Pipiza lugubris* (Fabricius, 1775)**

**Reference.**Tóth (1986) as *Pipiza signata* Meigen, 1822.

**Distribution.** Europe and Georgia.

**Remarks.**Vujić et al. (2013) synonymised *P. signata* under *P. lugubris*. The specimen collected and studied by Tóth (1986) needs verification.

***Pipiza noctiluca* (Linnaeus, 1758)**

**Reference.**Tóth (1986); Peck (1988); Gudjabidze (2002).

**Distribution.** Probably Europe, Russia and Turkey, but needs reassessment after Vujić et al. (2013).

***Pipizella annulata* (Macquart, 1829)**

**Reference.**Peck (1988).

**Distribution.** Europe.

**Remarks.** According to Van Steenis and Lucas (2011), records of this species from Turkey might belong to *Pipizella orientalis* Van Steenis & Lucas, 2011. It seems reasonable that the material from Transcaucasia cited by Peck (1988) might also belong to *P. orientalis*.

***Pipizella cornuta* Kuznetzov, 1987**

**Reference.**Kuznetzov (1987); Van Steenis and Lucas (2011); Barkalov and Mutin (2018).

**New records.** GEORGIA • 2♂; L16, 27 Jun 2018, S. Bot leg.

**Distribution.** Northern Caucasus and Georgia.

**Remarks.**Kuznetzov (1987), in the original description of this species, listed the type material from North Ossetia, but the type material studied by Van Steenis and Lucas (2011) was collected in South Ossetia, as authors pointed out (Van Steenis and Lucas 2011). Consequently, the records from Northern Caucasus need verification.

***Pipizella curvitibia* Stackelberg, 1960**

**Reference.**Van Steenis and Lucas (2011).

**Distribution.** North-east Turkey and Transcaucasia.

***Pipizella divicoi* (Goeldlin de Tiefenau, 1974)**

**Reference.**Kuznetzov (1987); Peck (1988) as *Pipizella divicoi* (Goeldlin de Tiefenau, 1974) and as *Pipizella opaca* Violovitsh, 1981; Van Steenis and Lucas (2011); Barkalov and Mutin (2018).

**New records.** GEORGIA • 1♂; L19, 29 Jun 2018, S. Bot leg.; • 4♂ 2♀; L39, 23–26 Jul 2018, malaise trap, X. Mengual, M. Espeland, B. Thormann leg.; ZFMK-DIP-00054187 = ZFMK-TIS-8000862, ZFMK-DIP-00054188 = ZFMK-TIS-8000863, ZFMK-DIP-00054189, ZFMK-DIP-00054190, ZFMK-TIS-8000866, ZFMK-TIS-8000867; • 1♂; L49, 4 Aug 2001, J.-H. Stuke leg.; ZFMK-DIP-00057830; • 12♂; L53, 1 Aug 2001, J.-H. Stuke leg.; ZFMK-DIP-00057818, ZFMK-DIP-00057819, ZFMK-DIP-00057820, ZFMK-DIP-00057821, ZFMK-DIP-00057822, ZFMK-DIP-00057823, ZFMK-DIP-00057824, ZFMK-DIP-00057825, ZFMK-DIP-00057826, ZFMK-DIP-00057827, ZFMK-DIP-00057828, ZFMK-DIP-00057829.

**Genetics.** We sequenced four specimens (MN622033, MN622034, MN622035, MN622036) with identical COI barcode. Our COI barcodes are very similar to other sequences from different species in BOLD systems, such as *Pipizella zeneggenensis* (Goeldlin de Tiefenau, 1974) (99.69–99.83% similarity), *P. divicoi* (99.69%), and *P. viduata* (Linnaeus, 1758) (99.69%).

**Distribution.** Palaearctic, but not in northern Africa.

***Pipizella nataliae* Kuznetzov, 1990**

**New records.** GEORGIA • 1♂; L17, 18 Jun 2018, S. Bot leg.

**Distribution.** Northern Caucasus, Georgia, and Turkey.

**Remarks.** Reported for Georgia for the first time.

***Pipizella orientalis* Van Steenis & Lucas, 2011**

**Reference.**Van Steenis and Lucas (2011); Speight (2018a).

**New records.** GEORGIA • 2♂; L39, 23–26 Jul 2018, malaise trap, X. Mengual, M. Espeland, B. Thormann leg.; ZFMK-DIP-00054191 = ZFMK-TIS-8000864, ZFMK-DIP-00054192 = ZFMK-TIS-8000865; • 9♂; L53, 1 Aug 2001, J.-H. Stuke leg.; ZFMK-DIP-00057809, ZFMK-DIP-00057810, ZFMK-DIP-00057811, ZFMK-DIP-00057812, ZFMK-DIP-00057813, ZFMK-DIP-00057814, ZFMK-DIP-00057815, ZFMK-DIP-00057816, ZFMK-DIP-00057817.

**Genetics.** We were able to sequence two specimens of this taxon (MN622037, MN622038), which is not present in BOLD systems or GenBank. The two COI barcodes were identical between them and very similar to other *Pipizella* species (100% similarity with sequences of *Pipizella annulata* and 99.24% similar to *P. zeneggenensis*).

**Distribution.** Georgia and Turkey.

***Pipizella vandergooti* Van Steenis & Lucas, 2011**

**New records.** GEORGIA • 1♂ 1♀; L29, 19 Jul 2018, X. Mengual leg.; ZFMK-DIP-00053865 = ZFMK-TIS-8005569, ZFMK-DIP-00053866 = ZFMK-TIS-8005577; • 1♂; L57, 3 Aug 2001, J.-H. Stuke leg.; ZFMK-DIP-00057831; • 2♀; L70, 30 Jun–14 Jul 2018, malaise trap, GGBC-members leg.; ZFMK-TIS-8002725, ZFMK-TIS-8002726.

**Genetics.** Three specimens (a male and two females) were sequenced (MN622039, MN622040, MN622041) and their COI barcodes differ from 0% to 0.61%. This species was not present in BOLD systems or GenBank, and our sequences are identical (100% similarity) to COI sequences of specimens identified as *Pipizella pennina* (Goeldlin de Tiefenau, 1974) and *P. zeneggenensis*.

**Distribution.** Georgia and Turkey.

**Remarks.** We have assumed that the three collected females belong to this species based on the COI sequences and the co-occurrence with a male. Reported for Georgia for the first time.

***Pipizella viduata* (Linnaeus, 1758)**

**Reference.**Peck (1988) as *Pipizella varipes* (Meigen, 1822); Speight (2018a).

**Distribution.** Europe, North Africa, Transcaucasia, European parts of Russia into Siberia.

**Remarks.**Thompson et al. (1982) synonymised *P. varipes* under *P. viduata*.

***Pipizella virens* (Fabricius, 1805)**

**Reference.**Levitin (1962); Tóth (1986); Gudjabidze (2002) a *Pipizella virens* Fallen, 1817 [sic]; Barkalov and Mutin (2018).

**New records.** GEORGIA • 1♂ 1♀; L4, 18 Jun 2018, S. Bot leg.; • 1♂; L5, 18 Jun 2018, S. Bot leg.

**Distribution.** Palaearctic.

**Platycheirus (Platycheirus) albimanus (Fabricius, 1781)**

**Reference.**Tóth (1986); Peck (1988); Gudjabidze (2002) as *Platycheirus albimanus* (Fabricius, 1721) [sic].

**New records.** GEORGIA • 1♂; L2, 16 Jun 2018, S. Bot leg.; • 2♀; L3, 17 Jun 2018, S. Bot leg.; • 1♀; L5, 18 Jun 2018, S. Bot leg.; • 1♂ 2♀; L6, 19 Jun 2018, S. Bot leg.; • 1♀; L11, 23 Jun 2018, S. Bot leg.; • 1♂; L12, 24 Jun 2018, S. Bot leg.; • 2♂; L16, 27 Jun 2018, S. Bot leg.; • 1♂ 1♀; L19, 29 Jun 2018, S. Bot leg.; • 1♂; L20, 1 Jul 2018, S. Bot leg.; • 2♂; L29, 19 Jul 2018, X. Mengual leg.; ZFMK-DIP-00054027 = ZFMK-TIS-8001013, ZFMK-DIP-00053966 = ZFMK-TIS-8005527; • 2♀; L33, 23 Jul 2018, X. Mengual leg.; ZFMK-DIP-00053967 = ZFMK-TIS-8005532, ZFMK-DIP-00053968; • 1♀; L46, 24 Jul 2001, J.-H. Stuke leg.; ZFMK-DIP-00057331; • 1♀; L54, 2 Aug 2001, J.-H. Stuke leg.; ZFMK-DIP-00057332; • 3♀; L55, 31 Jul 2001, J.-H. Stuke leg.; ZFMK-DIP-00057335, ZFMK-DIP-00057336, ZFMK-DIP-00057337; • 9♂ 2♀; L57, 3 Aug 2001, J.-H. Stuke leg.; ZFMK-DIP-00057320, ZFMK-DIP-00057321, ZFMK-DIP-00057322, ZFMK-DIP-00057323, ZFMK-DIP-00057324, ZFMK-DIP-00057325, ZFMK-DIP-00057326, ZFMK-DIP-00057327, ZFMK-DIP-00057328, ZFMK-DIP-00057329, ZFMK-DIP-00057330; • 2♀; L57, 2 Aug 2001, J.-H. Stuke leg.; ZFMK-DIP-00057333, ZFMK-DIP-00057334; • 2♂ 1♀; L70, 30 Jun–14 Jul 2018, malaise trap, GGBC-members leg.; ZFMK-TIS-8002720, ZFMK-TIS-8002721, ZFMK-TIS-8002722; • 2♂ 2♀; L70, 30 Jun–14 Jul 2018, malaise trap, GGBC-members leg.; MTD-Dip-A-R-4590, MTD-Dip-A-R-4591; • 1♂ 1♀; L71, 30 Jun–14 Jul 2018, malaise trap, GGBC-members leg.; MTD-Dip-A-R-4594, ZFMK-TIS-8002756.

**Genetics.** We were able to sequence six specimens (MN622042, MN622043, MN622044, MN622045, MN622046, MN622047), all with identical COI barcodes. In BOLD systems there are two BINs with specimens of *P. albimanus*: BOLD:AAL7898 comprises specimens from Europe and northeast North America; and BOLD:ABY4282 has specimens from northwest North America.

**Distribution.** Holarctic and Philippines.

**Platycheirus (Platycheirus) ambiguus (Fallén, 1817)**

**Reference.**Tóth (1986); Peck (1988).

**Distribution.** Palaearctic, but it needs reassessment.

**Platycheirus (Platycheirus) angustipes Goeldlin de Tiefenau, 1974**

**New records.** GEORGIA • 1♂ 1♀; L52, 30 Jul 2001, J.-H. Stuke leg.; ZFMK-DIP-00057338, ZFMK-DIP-00012138 = ZFMK-TIS-2556546.

**Distribution.** Inadequately known, but reported from mountains of Central Europe and Pyrenees.

**Remarks.** Reported for Georgia for the first time.

**Platycheirus (Platycheirus) clypeatus (Meigen, 1822)**

**Reference.**Peck (1988); Gudjabidze (2002).

**New records.** GEORGIA • 1♀; L47, 25 Jul 2001, J.-H. Stuke leg.; ZFMK-DIP-00057342; • 4♂ 3♀; L53, 1 Aug 2001, J.-H. Stuke leg.; ZFMK-DIP-00057345, ZFMK-DIP-00057346, ZFMK-DIP-00057347, ZFMK-DIP-00057348, ZFMK-DIP-00057341, ZFMK-DIP-00057343, ZFMK-DIP-00057344.

**Distribution.** Holarctic.

**Platycheirus (Platycheirus) complicatus (Becker, 1889)**

**New records.** GEORGIA • 1♀; L3, 17 Jun 2018, S. Bot leg.; • 1♀; L19, 29 Jun 2018, S. Bot leg.

**Distribution.** Central Europe, eastwards to Siberia and Japan.

**Remarks.** Reported for Georgia for the first time.

**Platycheirus (Platycheirus) discimanus Loew, 1871**

**Reference.**Peck (1988).

**Distribution.** Holarctic.

**Platycheirus (Platycheirus) fulviventris (Macquart, 1829)**

**New records.** GEORGIA • 1♀; L47, 25 Jul 2001, J.-H. Stuke leg.; ZFMK-DIP-00057350; • 1♂; L48, 24 Jul 2001, J.-H. Stuke leg.; ZFMK-DIP-00057349.

**Distribution.** Palaearctic, but not in northern Africa.

**Remarks.** Reported for Georgia for the first time.

**Platycheirus (Pachysphyria) immaculatus Ôhara, 1980**

**New records.** GEORGIA • 2♀; L3, 17 Jun 2018, S. Bot leg.; • 1♀; L4, 18 Jun 2018, S. Bot leg.

**Distribution.** Palaeractic, including Nepal, but not in the Iberian Peninsula or in northern Africa.

**Remarks.** Reported for Georgia for the first time.

**Platycheirus (Platycheirus) immarginatus (Zetterstedt, 1849)**

**Reference.**Peck (1988); Gudjabidze (2002) as *Platycheirus immarginatus* Zetterstendt, 1843 [sic].

**Distribution.** Needs reassessment.

**Platycheirus (Platycheirus) manicatus (Meigen, 1822)**

**Reference.**Peck (1988); Gudjabidze (2002) as *Platycheirus manicatus* Meigen, 1822 and as *Platycheirus maniceris* Meigen, 1822 [sic].

**New records.** GEORGIA • 1♂; L4, 18 Jun 2018, S. Bot leg.; • 1♂; L6, 19 Jun 2018, S. Bot leg.; • 1♀; L11, 23 Jun 2018, S. Bot leg.; • 1♀; L11, 29 Jun 2018, S. Bot leg.; • 1♂ 2♀; L12, 24 Jun 2018, S. Bot leg.; • 1♂; L14, 25 Jun 2018, S. Bot leg.; • 1♀; L16, 27 Jun 2018, S. Bot leg.; • 2♂; L17, 28 Jun 2018, S. Bot leg.; • 2♀; L19, 29 Jun 2018, S. Bot leg.; • 2♀; L52, 30 Jul 2001, J.-H. Stuke leg.; ZFMK-DIP-00057354, ZFMK-DIP-00057355; • 1♀; L54, 2 Aug 2001, J.-H. Stuke leg.; ZFMK-DIP-00057356; • 3♀; L55, 31 Jul 2001, J.-H. Stuke leg.; ZFMK-DIP-00057352, ZFMK-DIP-00057353, ZFMK-DIP-00057357; • 1♂; L57, 2 Aug 2001, J.-H. Stuke leg.; ZFMK-DIP-00057351.

**Distribution.** Palaearctic, Greenland, and Alaska.

**Platycheirus (Platycheirus) migriaulii Stuke & Nielsen, 2002**

**Reference.**Stuke and Nielsen (2002); Speight (2018a).

**New records.** GEORGIA • 1♂; L17, 28 Jun 2018, S. Bot leg.

**Distribution.** Georgia.

**Platycheirus (Platycheirus) nielseni Vockeroth, 1990**

**New records.** GEORGIA • 1♂; L15, 26 Jun 2018, S. Bot leg.; • 1♂ 2♀; L52, 30 Jul 2001, J.-H. Stuke leg.; ZFMK-DIP-00057378, ZFMK-DIP-00057379, ZFMK-DIP-00057380.

**Distribution.** Holarctic.

**Remarks.** Reported for Georgia for the first time.

**Platycheirus (Platycheirus) peltatus (Meigen, 1822)**

**Reference.**Tóth (1986); Peck (1988); Gudjabidze (2002).

**New records.** GEORGIA • 1♂; L19, 29 Jun 2018, S. Bot leg.

**Distribution.** Central and Northern Europe, eastwards to Siberia and Japan.

**Platycheirus (Platycheirus) podagratus (Zetterstedt, 1838)**

**New records.** GEORGIA • 1♂ 1♀; L52, 30 Jul 2001, J.-H. Stuke leg.; ZFMK-DIP-00057382, ZFMK-DIP-00057381.

**Distribution.** Holarctic.

**Remarks.** Reported for Georgia for the first time.

**Platycheirus (Platycheirus) similis Barkalov & Nielsen, 2007**

**New records.** GEORGIA • 1♂; L11, 23 Jun 2018, S. Bot leg.; • 1♂; L20, 1 Jul 2018, S. Bot leg.

**Distribution.** Northern Caucasus.

**Remarks.** Reported for Georgia for the first time.

**Platycheirus (Platycheirus) scutatus (Meigen, 1822)**

**Reference.**Peck (1988).

**New records.** GEORGIA • 1♀; L57, 3 Aug 2001, J.-H. Stuke leg.; ZFMK-DIP-00057383.

**Distribution.** Palaearctic and western North America.

**Remarks.** Females of the *scutatus* species group are difficult to identify (Doczkal et al. 2002; but see Van Steenis and Goeldlin de Tiefenau 1998), and this record might need verification.

**Platycheirus (Platycheirus) tarsalis (Schummel, 1837)**

**New records.** GEORGIA • 2♂ 1♀; L3, 17 Jun 2018, S. Bot leg.; • 1♂; L6, 19 Jun 2018, S. Bot leg.; • 1♂; L8, 20 Jun 2018, S. Bot leg.; • 1♂; L10, 22 Jun 2018, S. Bot leg.; • 1♀; L19, 29 Jun 2018, S. Bot leg.; • 1♂; L20, 30 Jun 2018, S. Bot leg.; • 1♀; L20, 1 Jul 2018, S. Bot leg.; • 1♀; L35, 24 Jul 2018, X. Mengual leg.; ZFMK-DIP-00054049 = ZFMK-TIS-8000950.

**Genetics.** We sequenced one specimen (MN622048), which belongs to the BIN BOLD:ABZ5039. The BIN has a maximum distance between specimens of 0.46% (P-distance) and 1.8% (p-distance) with the nearest neighbour in BOLD systems, *Platycheirus manicatus* (BOLD:ACF0224).

**Distribution.** Palaearctic, but not in northern Africa.

**Remarks.** Reported for Georgia for the first time.

***Pocota personata* (Harris, 1779)**

**Reference.**Peck (1988); Speight (2018a).

**New records.** GEORGIA • 1♂; L19, 29 Jun 2018, S. Bot leg.

**Distribution.** Europe, European parts of Russia, and Transcaucasia.

**Remarks.** The year of publication for this species is a convention. The original work by Harris (1776–1780) was published in five ‘decads’ or parts. Peck (1988) used the conventional date of 1780? with a question mark for decads 3, 4, and 5 based on Lisney (1960). Evenhuis (1997: page 343) established that the decad 3, where *Musca personatus* is described on page 79, was dated as 1779 based on the latest date of the plates. Thus, the year of publication should be 1779.

***Psilota anthracina* Meigen, 1822**

**Reference.**Peck (1988).

**Distribution.** Europe, European parts of Russia and Transcaucasia, but needs reassessment.

***Pyrophaena rosarum* (Fabricius, 1787)**

**Reference.**Tóth (1986); Peck (1988).

**Distribution.** Holarctic, but not in northern Africa.

***Rhingia campestris* Meigen, 1822**

**Reference.**Tóth (1986); Peck (1988); Gudjabidze (2002); Speight (2018a).

**New records.** GEORGIA • 1♀; L3, 17 Jun 2018, S. Bot leg.; • 1♂ 2♀; L15, 26 Jun 2018, S. Bot leg.; • 1♀; L33, 23 Jul 2018, X. Mengual leg.; ZFMK-DIP-00054112 = ZFMK-TIS-8000988; • 1♀; L52, 30 Jul 2001, J.-H. Stuke leg.; ZFMK-DIP-00058253.

**Genetics.** One specimen was successfully sequenced (MN622049), and its BIN is BOLD:ABZ3049. Our sequence is exactly the same as other sequences of *R. campestris* previously published.

**Distribution.** Palaearctic.

***Rhingia rostrata* (Linnaeus, 1758)**

**Reference.**Levitin (1962); Tóth (1986); Peck (1988); Gudjabidze (2002) as *Rhingia rostrata* Linnaeus, 1758 [sic]; Speight (2018a).

**New records.** GEORGIA • 1♂ 1♀; L1, 15 Jun 2018, S. Bot leg.; • 1♀; L39, 23–26 Jul 2018, X. Mengual, M. Espeland, B. Thormann leg.; ZFMK-DIP-00054113 = ZFMK-TIS-8000879; • 1♀; L71, 30 Jun–14 Jul 2018, malaise trap, GGBC-members leg.; ZFMK-TIS-8002734; • 1♂; L72, 29 Jun–13 Jul 2018, malaise trap, GGBC-members leg.; ZFMK-TIS-8002770, MTD-Dip-A-R-4574.

**Genetics.** We sequenced three specimens (MN622050, MN622051, MN622052) and their COI barcodes differ 0.15–0.3%. The BIN for these specimens is BOLD:AAV1208 and its nearest neighbour in BOLD systems is *Rhingia nasica* Say, 1823(BOLD:AAG4646; 4.8% p-distance).

**Distribution.** Europe, Transcaucasia, and European parts of Russia into Siberia.

***Riponnensia longicornis* (Loew, 1843)**

**Reference.**Peck (1988) as *Orthonevra longicornis* (Loew, 1843).

**Distribution.** Mediterranean Basin.

***Riponnensia splendens* (Meigen, 1822)**

**Reference.**Peck (1988) as *Orthonevra splendens* (Meigen, 1822); Gudjabidze (2002) as *Orthonevra splendens* Meigen, 1822 [sic]; Speight (2018a).

**Distribution.** Western Palaearctic.

***Rohdendorfia alpina* Sack, 1938**

**Reference.**Barkalov and Mutin (2018); Speight (2018a); Mengual and Barkalov (2019).

**Distribution.** Mountains of Central Europe, Caucasus, and Altai.

**Remarks.**Barkalov and Mutin (2018) and Speight (2018a) listed this species from Transcaucasia, but it was never reported previously from Georgia to our knowledge. Mengual and Barkalov (2019) reported it for Georgia for the first time.

**Scaeva (Scaeva) albomaculata (Macquart, 1842)**

**Reference.**Kuznetzov (1985); Tóth (1986) as *Scaeva albomaculata* Macquart, 1842 [sic]; Peck (1988); Gudjabidze (2002) as *Scaeva albomaculata* Macquart, 1827 [sic]; Barkalov and Mutin (2018); Speight (2018a).

**New records.** GEORGIA • 1♀; L34, 22 Jul 2018, X. Mengual leg.; ZFMK-DIP-00054100 = ZFMK-TIS-8000994.

**Genetics.** One specimen was sequenced (MN622053) and its COI barcode is very similar (98.73–99.69% similarity) to other sequences in BOLD of *S. albomacualta*. The intraspecific p-distance overlaps the interspecific p-distance with *Scaeva pyrastri* (Linnaeus, 1758) (98.62–98.81% similarity) and both species share the same BIN, BOLD:AAF2374.

**Distribution.** Palaearctic, but not in Central and Northern Europe.

**Scaeva (Scaeva) pyrastri (Linnaeus, 1758)**

**Reference.**Radde (1899) as *Syrphus pyraster* L.; Levitin (1962); Kuznetzov (1985); Peck (1988); Gudjabidze (2002) as *Scaeva pyrastri* Linnaeus, 1758 [sic].

**New records.** GEORGIA • *1; L1, 16 Jun 2018, S. Bot obs.; • *1; L18, 28 Jun 2018, S. Bot obs.; • 1♀; L20, 1 Jul 2018, S. Bot leg.; • 1♀; L29, 19 Jul 2018, X. Mengual leg.; ZFMK-DIP-00053852 = ZFMK-TIS-8005595; • 1♀; L34, 22 Jul 2018, X. Mengual leg.; ZFMK-DIP-00053853 = ZFMK-TIS-8005601; • 1♀; L57, 3 Aug 2001 J.-H. Stuke leg.; ZFMK-DIP-00058247.

**Genetics.** Two specimens were sequenced (MN622054, MN622055) and their COI sequences differ 0.15%. See Genetics under *Scaeva albomaculata*.

**Distribution.** Palaearctic, India, and western North America.

**Scaeva (Semiscaeva) dignota (Rondani, 1857)**

**Reference.**Kuznetzov (1985) as *Scaeva odessana* (Paramonov, 1924); Peck (1988).

**New records.** GEORGIA • 1♀; L1, 16 Jun 2018, S. Bot leg.; • 1♂ 1♀; L3, 17 Jun 2018, S. Bot leg.; • 1♂; L8, 20 Jun 2018, S. Bot leg.; • 1♀; L16, 27 Jun 2018, S. Bot leg.; • 8♂ 3♀; L17, 28 Jun 2018, S. Bot leg.; • *50♂; L17, 28 Jun 2018, S. Bot obs.; • 1♀; L51, 24 Jul 2001, J.-H. Stuke leg.; ZFMK-DIP-00057873.

**Distribution.** Western Palaearctic.

**Remarks.** See remarks under Scaeva (Semiscaeva) lagodechiensis Kuznetzov, 1985. Dušek and Láska (1985) synonymised *Catabomba odessana* Paramonov, 1924 under *S. dignota*.

**Scaeva (Semiscaeva) lagodechiensis Kuznetzov, 1985**

**Reference.**Kuznetzov (1985); Barkalov and Mutin (2018).

**Distribution.** Georgia.

**Remarks.**Kuznetzov (1985) described this species based on three males from Lagodekhi (Georgia). This species is very similar to *Scaeva dignota*. The differences with *S. dignota* given in Kuznetzov (1985) are subtle and sometimes they also apply to individuals identified in this paper as *S. dignota*. More research is needed but *S. lagodechiensis* might be a junior synonym of *S. dignota*.

**Scaeva (Semiscaeva) opimia (Walker, 1852)**

**Reference.**Kuznetzov (1987) as *Scaeva lunata* (Wiedemann, 1830); Peck (1988) as *Scaeva lunata* (Wiedemann, 1830).

**Distribution.** Transcaucasia, Afghanistan, China, and Indomalayan Region.

**Remarks.**Mengual et al. (2018) explained that *Scaeva opimia* (Walker, 1852) is the right name to apply to this taxon, not *Scaeva lunata*.

**Scaeva (Semiscaeva) selenitica (Meigen, 1822)**

**Reference.**Levitin (1962); Kuznetzov (1985) as *Scaeva selenitica* and *Scaeva rossica* Kuznetzov, 1985; Tóth (1986); Peck (1988); Gudjabidze (2002) as *Scaeva selenitika* Meigen, 1822 [sic].

**New records.** GEORGIA • 1♂; L4, 18 Jun 2018, S. Bot leg.; • 1♀; L8, 20 Jun 2018, S. Bot leg.; • 1♂; L10, 22 Jun 2018, S. Bot leg.

**Distribution.** Palaearctic.

***Sericomyia bequaerti* (Hervé-Bazin, 1913)**

**Reference.**Peck (1988) as *Arctophila bequaerti* Hervé-Bazin, 1913; Gudjabidze (2002) as *Arctophila bequarti* Have-Basin, 1914 [sic]; Barkalov and Mutin (2018); Speight (2018a).

**New records.** GEORGIA • 1♀; L19, 29 Jun 2018, S.Bot leg.

**Distribution.** Balkan Peninsula, Turkey, south-western Russia, Ukraine, and Transcaucasia.

**Remarks.**Skevington and Thompson (2012) synonymised *Arctophila* Schiner, 1860 and *Conosyrphus* Frey, 1915 under *Sericomyia* Meigen, 1803.

***Sericomyia bombiformis* (Fallén, 1810)**

**Reference.**Peck (1988) as *Arctophila bombiformis* (Fallén, 1810); Gudjabidze (2002) as *Arctophila bomboformis* (Fallen, 1817) [sic].

**Distribution.** Europe, Turkey, and Georgia.

***Sericomyia silentis* (Harris, 1778)**

**Reference.**Levitin (1962) as *Cinxia borealis ciscaucasica* Stackelberg, 1927; Peck (1988) as *Sericomyia silentis* and *Sericomyia silentis ciscaucasica* (Stackelberg, 1927); Gudjabidze (2002) as *Sericomyia borealis ciscausica* Shtackelberg, 1976 [sic]; Speight (2018a).

**New records.** GEORGIA • *1♂; L15, 26 Jun 2018, S. Bot obs.; • 1♀; L15, 26 Jun 2018, S. Bot leg.; • 1♀; L16, 27 Jun 2018, S. Bot leg.

**Distribution.** Palaearctic, but not in northern Africa.

**Remarks.** The year of publication for this species is a convention. Peck (1988) used the conventional dates based on Lisney (1960): 1776 for decad 1, 1776? for decad 2, and 1780? for decads 3, 4, and 5. Evenhuis (1997: page 342) found that the decad 2, where *Musca silentis* is described on page 59, was dated as 1778 in the “*Discours préliminaires*” to the *Encyclopédie méthodique par ordre des matières* – *Insectes*. Thus, the year of publication should be 1778.

***Sericomyia volucellina* Portschinsky, 1881**

**Reference.**Peck (1988) as *Conosyrphus volucellinus* (Portschinsky, 1881); Gudjabidze (2002) as *Conosyrphus volucelluns* Portshinsky, 1881 [sic] and as *Conosyrphus volucellinus* Portshinski, 1881 [sic]; Barkalov and Mutin (2018); Speight (2018a).

**New records.** GEORGIA • 1♂; L11, 29 Jun 2018, S.Bot leg.; • 1♂; L57, 2 Aug 2001, J.-H. Stuke leg.; ZFMK-DIP-00058000.

**Distribution.** Transcaucasia and Turkey.

***Spazigaster ambulans* (Fabricius, 1798)**

**Reference.**Peck (1988); Barkalov and Mutin (2018); Speight (2018a).

**New records.** GEORGIA • 1♀; L4, 18 Jun 2018, S. Bot leg.; • 1♀; L11, 29 Jun 2018, S. Bot leg.; • 8♂; L12, 24 Jun 2018, S. Bot leg.; • 1♀; L33, 22 Jul 2018, X. Mengual leg.; ZFMK-DIP-00054102 = ZFMK-TIS-8000877; • 4♂ 9♀; L52, 30 Jul 2001, J.-H. Stuke leg.; ZFMK-DIP-00057433, ZFMK-DIP-00057434, ZFMK-DIP-00057435, ZFMK-DIP-00057445, ZFMK-DIP-00057436, ZFMK-DIP-00057437, ZFMK-DIP-00057438, ZFMK-DIP-00057439, ZFMK-DIP-00057440, ZFMK-DIP-00057441, ZFMK-DIP-00057442, ZFMK-DIP-00057443, ZFMK-DIP-00057444.

**Genetics.** We sequenced one specimen (MN622056), and its COI barcode is identical as other sequenced specimens listed in BOLD and very similar (> 98.62% similarity) to other non-public and published sequences of this species. The BIN for this taxon is BOLD:AAZ5247.

**Distribution.** Mountains of Central Europe, Balkans, Carpathians, Turkey and the Caucasus Region.

**Sphaerophoria (Sphaerophoria) bengalensis Macquart, 1842**

**Reference.**Bańkowska (1964) as *Sphaerophoria turkmenica* Bańkowska, 1964; Peck (1988) as *Sphaerophoria turkmenica*; Gudjabidze (2002) as *Sphaerophoria turkmenica* Bankovska, 1964 [sic]; Speight (2018a) listed it only from Armenia and Azerbaijan.

**New records.** GEORGIA • 1♂; L31, 20 Jul 2018, X. Mengual leg.; ZFMK-DIP-00053867 = ZFMK-TIS-8005599; • 1♂; L50, 4 Aug 2001, J.-H. Stuke leg.; ZFMK-DIP-00058250; • 2♂; L59, 28 Jul 2001, J.-H. Stuke leg.; ZFMK-DIP-00058248, ZFMK-DIP-00058249.

**Genetics.** We sequenced one specimen (MN622058) and its COI barcode is very similar (99.85%) with a sequence of *Sphaerophoria scripta* (Linnaeus, 1758).

**Distribution.** Middle East, Arabian Peninsula, Transcaucasia, Central and Eastern Palaearctic, Pakistan, and northern India.

**Sphaerophoria (Sphaerophoria) boreoalpina Goeldlin de Tiefenau, 1989**

**New records.** GEORGIA • 1♂; L52, 30 Jul 2001, J.-H. Stuke leg.; ZFMK-DIP-00057908.

**Distribution.** Northern Europe, Alps, and Altai Mountains.

**Remarks.** Reported for Georgia for the first time.

**Sphaerophoria (Sphaerophoria) chongjini Bańkowska, 1964**

**New records.** GEORGIA • 2♂; L31, 20 Jul 2018, X. Mengual leg.; ZFMK-DIP-00054057 = ZFMK-TIS-8000874, ZFMK-DIP-00053868 = ZFMK-TIS-8005598; • 2♂; L72, 29 Jun–13 Jul 2018, malaise trap, GGBC-members leg.; ZFMK-TIS-8002779, ZFMK-DIP-00061315; • 2♂; L72, 29 Jun–13 Jul 2018, malaise trap, GGBC-members leg.; MTD-Dip-A-R-4584.

**Genetics.** Three male specimens (MN622059, MN622060, MN622061) were sequenced and they differ very little (0–0.15%). Our COI barcodes are identical to another specimen of *S. chongjini* in BOLD systems, but also has 100% similarity with a barcode of *Sphaerophoria taeniata* (Meigen, 1822).

**Distribution.** Northern and Central Europe, Ukraine, and Russia into Japan.

**Remarks.** Reported for Georgia for the first time.

**Sphaerophoria (Sphaerophoria) infuscata Goeldlin de Tiefenau, 1974**

**New records.** GEORGIA • 2♂; L52, 30 Jul 2001 J.-H. Stuke leg.; ZFMK-DIP-00057909, ZFMK-DIP-00057910.

**Distribution.** Central Europe, Alps, and Pyrenees.

**Remarks.** Reported for Georgia for the first time.

**Sphaerophoria (Sphaerophoria) interrupta (Fabricius, 1805)**

**Reference.**Levitin (1962) as *Sphaerophoria menthastri* L.; Tóth (1986) as *Sphaerophoria menthastri* (Linnaeus, 1758); Peck (1988) as *S. menthastri*; Gudjabidze (2002) as *Sphaerophoria mentastri* Linnaeus, 1758 [sic] and *Sphaerophoria picta* Meigen, 1882 [sic]; Speight (2018a).

**Distribution.** Europe, Transcaucasia, and European parts of Russia into Siberia.

**Remarks.**Goeldlin de Tiefenau (1989) explained that the name *menthastri* Linnaeus cannot be applied to this taxon and reinstated the name *interrupta* Fabricius for *menthastri* sensu auctores nec L. Peck (1988) listed *Syrphus pictus* Meigen, 1822 as synonym of *Sphaerophoria menthastri* (Linnaeus, 1758).

**Sphaerophoria (Sphaerophoria) laurae Goeldlin de Tiefenau, 1989**

**Reference.**Speight (2018a) listed it from the Caucasus.

**Distribution.** Northern Europe, Alps, Pyrenees, Caucasus, and Altai Mountains.

**Sphaerophoria (Sphaerophoria) philanthus (Meigen, 1822)**

**Reference.**Peck (1988); Gudjabidze (2002) as *Sphaerophoria sarmatica* Bankovska, 1964 [sic] and as and *Sphaerophoria dubia* Zetterstendt, 1849 [sic].

**Distribution.** Needs reassessment, but known from Northern and Central Europe and North America.

**Remarks.***Sphaerophoria sarmatica* Bańkowska, 1964 and and *Sphaerophoria dubia* Zetterstedt, 1849 were listed as synonyms of *S. philanthus* by Peck (1988).

**Sphaerophoria (Sphaerophoria) rueppellii (Wiedemann, 1830)**

**Reference.**Bańkowska (1964); Peck (1988); Gudjabidze (2002).

**New records.** GEORGIA • 1♂; L48, 24 Jul 2001, J.-H. Stuke leg.; ZFMK-DIP-00057916; • 1♀; L50, 4 Aug 2001, J.-H. Stuke leg.; ZFMK-DIP-00057918; • 1♂; L53, 1 Aug 2001, J.-H. Stuke leg.; ZFMK-DIP-00057915; • 1♀; L59, 28 Jul 2001, J.-H. Stuke leg.; ZFMK-DIP-00057919; • 1♂; L63, 23 Jul 2001, J.-H. Stuke leg.; ZFMK-DIP-00057914; • 3♂ 1♀; L67, 29 Jul 2001, J.-H. Stuke leg.; ZFMK-DIP-00057911, ZFMK-DIP-00057912, ZFMK-DIP-00057913, ZFMK-DIP-00057917; • 1♀; L72, 29 Jun–13 Jul 2018, malaise trap, GGBC-members leg.; ZFMK-TIS-8002777.

**Genetics.** We sequenced a single specimen (MN622062) and its COI barcode was identical to other published sequences of the same species, but also 100% similar to sequences of *Sphaerophoria scripta* (Linnaeus, 1758).

**Distribution.** Palaearctic and Afrotropical Region.

**Sphaerophoria (Sphaerophoria) scripta (Linnaeus, 1758)**

**Reference.**Levitin (1962); Tóth (1986); Peck (1988); Gudjabidze (2002) as *S. scripta*.

**New records.** GEORGIA • 1♂; L1, 15 Jun 2018, S. Bot leg.; • *20; L1, 16 Jun 2018, S. Bot obs.; • 2♂ 1♀; L2, 16 Jun 2018, S. Bot leg.; • 1♂; L4, 18 Jun 2018, S. Bot leg.; • *15; L8, 20 Jun 2018, S. Bot obs.; • 2♂ *20; L10, 22 Jun 2018, S. Bot leg. & obs.; • *10; L11, 23 Jun 2018, S. Bot obs.; • 2♀; L15, 26 Jun 2018, S. Bot leg.; • *10; L19, 29 Jun 2018, S. Bot obs.; • 1♂; L20, 1 Jul 2018, S. Bot leg.; • 1♀; L24, 17 Jul 2018, X. Mengual leg.; ZFMK-DIP-00054073 = ZFMK-TIS-8000970; • 2♀; L26, 18 Jul 2018, X. Mengual leg.; ZFMK-DIP-00054069, ZFMK-DIP-00054077; • 1♂; L27, 19 Jul 2018, X. Mengual leg.; ZFMK-DIP-00053777; • 1♀; L28, 19 Jul 2018, X. Mengual leg.; ZFMK-DIP-00054072 = ZFMK-TIS-8000979; • 1♂ 1♀; L29, 19 Jul 2018, X. Mengual leg.; ZFMK-DIP-00053778 = ZFMK-TIS-8005603, ZFMK-DIP-00054066 = ZFMK-TIS-8001010; • 4♂ 1♀; L30, 19 Jul 2018, X. Mengual leg.; ZFMK-DIP-00053779, ZFMK-DIP-00053780, ZFMK-DIP-00053781, ZFMK-DIP-00053782, ZFMK-DIP-00053783; • 1♀; L31, 20 Jul 2018, J. Astrin leg.; ZFMK-TIS-8000113; • 3♂ 1♀; L31, 20 Jul 2018, X. Mengual leg.; ZFMK-DIP-00054058, ZFMK-DIP-00054061, ZFMK-DIP-00054062, ZFMK-DIP-00054078; • 2♀; L32, 22 Jul 2018, X. Mengual leg.; ZFMK-DIP-00054065 = ZFMK-TIS-8001007, ZFMK-DIP-00054070 = ZFMK-TIS-8001005; • 2♂ 1♀; L32, 22 Jul 2018, X. Mengual leg.; ZFMK-DIP-00053770, ZFMK-DIP-00053771, ZFMK-DIP-00053784; • 4♂ 1♀; L33, 22 Jul 2018, X. Mengual leg.; ZFMK-DIP-00054064, ZFMK-DIP-00053772, ZFMK-DIP-00053773, ZFMK-DIP-00053774, ZFMK-DIP-00054068; • 1♂; L34, 22 Jul 2018, X. Mengual leg.; ZFMK-DIP-00053775; • 2♂; L35, 24 Jul 2018, X. Mengual leg.; ZFMK-DIP-00054059, ZFMK-DIP-00053776; • 1♂ 2♀; L37, 25 Jul 2018, B. Thormann leg.; ZFMK-DIP-00054060 = ZFMK-TIS-8004102, ZFMK-DIP-00054071 = ZFMK-TIS-8004112, ZFMK-DIP-00054076 = ZFMK-TIS-8004103; • 2♂; L37, 25 Jul 2018, X. Mengual leg.; ZFMK-DIP-00054063, ZFMK-DIP-00053769; • 1♂; L38, 25 Jul 2018, X. Mengual leg.; ZFMK-DIP-00053768 = ZFMK-TIS-8005597; • 3♀; L39, 23–26 Jul 2018, malaise trap, X. Mengual, M. Espeland, B. Thormann leg.; ZFMK-DIP-00054067, ZFMK-DIP-00054074, ZFMK-DIP-00054075; • 2♂; L42, 25 Jul 2018, A. Reimann leg.; MTD-Dip-A-R-4536; MTD-Dip-A-R-4543; • 2♂; L42, 25 Jul 2018, B. Rulik leg.; MTD-Dip-A-R-4546, ZFMK-TIS-8002678; • 2♂; L47, 25 Jul 2001, J.-H. Stuke leg.; ZFMK-DIP-00058252, ZFMK-DIP-00057907; • 1♂; L48, 24 Jul 2001, J.-H. Stuke leg.; ZFMK-DIP-00057904; • 1♂; L49, 4 Aug 2001, J.-H. Stuke leg.; ZFMK-DIP-00058251; • 1♂; L51, 24 Jul 2001, J.-H. Stuke leg.; ZFMK-DIP-00057887; • 1♂; L53, 1 Aug 2001, J.-H. Stuke leg.; ZFMK-DIP-00057893; • 1♂; L56, 30 Jul 2001, J.-H. Stuke leg.; ZFMK-DIP-00057902; • 3♂; L59, 28 Jul 2001, J.-H. Stuke leg.; ZFMK-DIP-00057884, ZFMK-DIP-00057889, ZFMK-DIP-00057890; • 2♂; L60, 26 Jul 2001, J.-H. Stuke leg.; ZFMK-DIP-00057892, ZFMK-DIP-00057905; • 9♂; L63, 23 Jul 2001, J.-H. Stuke leg.; ZFMK-DIP-00057883, ZFMK-DIP-00057885, ZFMK-DIP-00057891, ZFMK-DIP-00057894, ZFMK-DIP-00057896, ZFMK-DIP-00057897, ZFMK-DIP-00057898, ZFMK-DIP-00057899, ZFMK-DIP-00057900; • 1♂; L64, 23 Jul 2001, J.-H. Stuke leg.; ZFMK-DIP-00057901; • 1♂; L65, 23 Jul 2001, J.-H. Stuke leg.; ZFMK-DIP-00057888; • 3♂; L67, 29 Jul 2001, J.-H. Stuke leg.; ZFMK-DIP-00057886, ZFMK-DIP-00057903, ZFMK-DIP-00057906; • 1♂; L69, 18 Jul 2018, A. Reimann leg.; MTD-Dip-A-R-4503; • 4♂ 1♀; L70, 30 Jun–14 Jul 2018, malaise trap, GGBC-members leg.; ZFMK-TIS-8002707, ZFMK-TIS-8002708, ZFMK-DIP-00061316, ZFMK-DIP-00061317, ZFMK- TIS-8002709; • 2♀; L70, 30 Jun–14 Jul 2018, malaise trap, GGBC-members leg.; MTD-Dip-A-R-4588; • 1♂ 1♀; L71, 30 Jun–14 Jul 2018, malaise trap, GGBC-members leg.; ZFMK-TIS-8002747, ZFMK-TIS-8002744; • 3♂; L72, 29 Jun–13 Jul 2018, malaise trap, GGBC-members leg.; ZFMK-TIS-8002774, ZFMK-TIS-8002775, ZFMK-DIP-00061314; • 2♂;L72, 29 Jun–13 Jul 2018, malaise trap, GGBC-members leg.; MTD-Dip-A-R-4559.

**Genetics.** We sequenced ten specimens of this species (MN622057, MN622063, MN622064, MN622065, MN622066, MN622067, MN622068, MN622069, MN622070, MN622071), and their COI sequences differ 0–0.83%. The intraspecific distance in this genus overlaps with the interspecific distance of the COI barcodes. As a prove, the BIN BOLD:AAA7374 has specimens from more than 30 different species and it suggests that the COI sequence alone is not useful to separate *Sphaerophoria* species.

**Distribution.** Palaearctic, Greenland, Kashmir, and Nepal.

**Sphegina (Asiosphegina) sibirica Stackelberg, 1953**

**Reference.**Tóth (1986); Peck (1988); Speight (2018a).

**New records.** GEORGIA • 2♂; L10, 22 Jun 2018, S. Bot leg.; • 1♂; L16, 27 Jun 2018, S. Bot leg.; • 1♂; L20, 30 Jun 2018, S. Bot leg.; • 2♂; L21, 3 Jul 2018, S. Bot leg.

**Distribution.** Northern and Central Europe, Transcaucasia, European parts of Russia to Far East.

**Sphegina (Sphegina) alaoglui Hayat, 1997**

**New records.** GEORGIA • 2♂; L3, 17 Jun 2018, S. Bot leg.; • 1♂; L20, 1 Jul 2018, S. Bot leg.; 1♂ 1♀; L71, 30 Jun–14 Jul 2018, malaise trap, GGBC-members leg.; ZFMK-TIS-8002760, ZFMK-TIS-8002810; • 1♂; L71, 30 Jun–14 Jul 2018, malaise trap, GGBC-members leg.; MTD-Dip-A-R-4561.

**Genetics.** A male and a female were sequenced (MN622072, MN622073) and their COI barcodes are 99.696% similar. This species was not registered in BOLD systems or GenBank, but our COI sequences are very similar to sequences of *Sphegina elegans* Schummel, 1843 (96.31–96.64% similarity).

**Distribution.** Turkey and Northern Caucasus.

**Remarks.** Species similar to Sphegina (Sphegina) elegans Schummel, 1843. Mutin (2001a) synonymised *Sphegina pontica* Mutin, 1998 under *S. alaoglui*. Reported for Georgia for the first time.

**Sphegina (Sphegina) clunipes (Fallén, 1816)**

**Reference.**Tóth (1986); Peck (1988); Gudjabidze (2002); Barkalov and Mutin (2018); Speight (2018a).

**New records.** GEORGIA • 3♂; L1, 16 Jun 2018, S. Bot leg.; • 1♀; L3, 17 Jun 2018, S. Bot leg.; • 1♀; L5, 18 Jun 2018, S. Bot leg.; • 1♂; L6, 19 Jun 2018, S. Bot leg.

**Distribution.** Palaearctic, but not in northern Africa.

**Sphegina (Sphegina) elegans Schummel, 1843**

**Reference.**Peck (1988) as *Sphegina kimakowiczi* (Strobl, 1897); Speight (2018a).

**Distribution.** Europe, European parts of Russia, Turkey, and Caucasus Mountains.

**Remarks.**Thompson and Torp (1986) synonymised *S. kimakowiczi* under *S. elegans*.

**Sphegina (Sphegina) obscurifacies Stackelberg, 1956**

**Reference.**Speight (2018a) listed it from the Caucasus.

**Distribution.** Northern Europe, Caucasus Region, Russia and Korea.

**Remarks.** See Speight (2018a) and Mutin (2001b) for the common confusion of this taxon with Sphegina (Sphegina) claviventris Stackelberg, 1956.

**Sphegina (Sphegina) verecunda Collin, 1937**

**Reference.**Peck (1988); Gudjabidze (2002) as *Sphegina verecunda* Collin, 1931 [sic].

**Distribution.** Central Europe and Transcaucasia.

***Sphiximorpha subsessilis* (Illiger in Rossi, 1807)**

**Reference.**Peck (1988); Speight (2018a).

**Distribution.** Western Palaearctic.

***Spilomyia diophthalma* (Linnaeus, 1758)**

**Reference.**Stackelberg (1958); Levitin (1962); Peck (1988); Gudjabidze (2002) as *Spilomyia diophtalma* Linnaeus, 1758 [sic]; Speight (2018a).

**Distribution.** Northern and Central Europe, Turkey, Transcaucasia, through Russia to Sakhalin.

***Spilomyia manicata* (Rondani, 1865)**

**Reference.**Stackelberg (1958); Levitin (1962); Peck (1988); Van Steenis (2000); Gudjabidze (2002) as *Spilomyia manicata* Rondani, 1862 [sic]; Barkalov and Mutin (2018); Speight (2018a).

**New records.** GEORGIA • 1♂; L37, 25 Jul 2018, B. Thormann leg.; ZFMK-DIP-00054101 = ZFMK-TIS-8004097.

**Genetics.** The collected male was sequenced (MN622074) and its COI barcode is identical to another sequence published in BOLD systems of *S. manicata* from Norway (Sample ID: NorSy408; BIN: BOLD:ACC9767).

**Distribution.** Europe, European parts of Russia, and Caucasus Region.

***Spilomyia saltuum* (Fabricius, 1794)**

**Reference.**Stackelberg (1958); Peck (1988); Barkalov and Mutin (2018); Speight (2018a).

**New records.** GEORGIA • 1♂; L64, 23 Jul 2001, J.-H. Stuke leg.; ZFMK-DIP-00012151 = ZFMK-TIS-2558698.

**Distribution.** Central Europe, Mediterranean Basin, and the Caucasus Region.

***Spilomyia triangulata* Van Steenis, 2000**

**Reference.**Stackelberg (1958) as *Spilomyia digitata* (Rondani, 1865); Peck (1988) as *Spilomyia digitata*.

**Distribution.** Alps, North Macedonia, Greece, Turkey, and Transcaucasia.

**Remarks.**Van Steenis (2000) described this taxon from the eastern part of the Mediterranean Basin (with a record from France), and among the paratypes he listed material collected in Gelendzhik (Northern Caucasus); likely the same specimens that Stackelberg (1958) reported as *S. digitata* from Gelendzhik and, later on, Peck (1988) listed from Transcaucasia.

***Syritta pipiens* (Linnaeus, 1758)**

**Reference.**Levitin (1962); Tóth (1986); Peck (1988); Gudjabidze (2002).

**New records.** GEORGIA • 1♂; L2, 16 Jun 2018, S. Bot leg.; • 1♀; L3, 17 Jun 2018, S. Bot leg.; • 1♂ 1♀; L11, 23 Jun 2018, S. Bot leg.; • 1♀; L19, 29 Jun 2018, S. Bot leg.; • *2; L22, 3 Jul 2018, S. Bot obs.; • 1♂; L23, 5 Jul 2018, S. Bot leg.; • 2♀; L33, 22 Jul 2018, X. Mengual leg.; ZFMK-DIP-00053849 = ZFMK-TIS-8005542, ZFMK-DIP-00054110; • 3♂; L39, 23–26 Jul 2018, X. Mengual, M. Espeland, B. Thormann leg.; ZFMK-DIP-00054108, ZFMK-DIP-00054109, ZFMK-DIP-00054111 = ZFMK-TIS-8000878; • 1♂; L46, 24 Jul 2001, J.-H. Stuke leg.; ZFMK-DIP-00058059; • 1♂ 2♀; L48, 24 Jul 2001, J.-H. Stuke leg.; ZFMK-DIP-00058057, ZFMK-DIP-00058063, ZFMK-DIP-00058065; • 1♂; L49, 4 Aug 2001, J.-H. Stuke leg.; ZFMK-DIP-00058060; • 7♂; L58, 27 Jul 2001, J.-H. Stuke leg.; ZFMK-DIP-00058048, ZFMK-DIP-00058049, ZFMK-DIP-00058050, ZFMK-DIP-00058051, ZFMK-DIP-00058052, ZFMK-DIP-00058053, ZFMK-DIP-00058054; • 2♂; L59, 28 Jul 2001, J.-H. Stuke leg.; ZFMK-DIP-00058055, ZFMK-DIP-00058056; • 1♀; L60, 26 Jul 2001, J.-H. Stuke leg.; ZFMK-DIP-00058066; • 1♂; L63, 23 Jul 2001, J.-H. Stuke leg.; ZFMK-DIP-00058058; • 1♀; L66, 28 Jul 2001, J.-H. Stuke leg.; ZFMK-DIP-00058064; • 2♂; L68, 29 Jul 2001, J.-H. Stuke leg.; ZFMK-DIP-00058061, ZFMK-DIP-00058062; • 1♀; L72, 29 Jun–13 Jul 2018, malaise trap, GGBC-members leg.; ZFMK-TIS-8002773.

**Genetics.** We sequenced three specimens (MN622075, MN622076, MN622077) and their COI barcodes differ 0.15–0.46%. The Barcode Index Number Registry lists a BIN for this taxon (BOLD:AAC6291) with an average 0.2% p-distance variation within the BIN (1.93% maximum p-distance) and 5.08% p-distance to the nearest neighbour, *Syritta fasciata* (Wiedemann, 1830) (BOLD:AAY9920).

**Distribution.** Most of Palaearctic, most of Nearctic, South America, and Indomalayan Region.

***Syrphocheilosia claviventris* (Strobl, 1910)**

**New records.** GEORGIA • 2♂ 23♀; L52, 30 Jul 2001, J.-H. Stuke leg.; ZFMK-DIP-00057446, ZFMK-DIP-00057447, ZFMK-DIP-00057448, ZFMK-DIP-00057449, ZFMK-DIP-00057450, ZFMK-DIP-00057451, ZFMK-DIP-00057452, ZFMK-DIP-00057453, ZFMK-DIP-00057454, ZFMK-DIP-00057455, ZFMK-DIP-00057456, ZFMK-DIP-00057457, ZFMK-DIP-00057458, ZFMK-DIP-00057459, ZFMK-DIP-00057460, ZFMK-DIP-00057461, ZFMK-DIP-00057462, ZFMK-DIP-00057463, ZFMK-DIP-00057464, ZFMK-DIP-00057465, ZFMK-DIP-00057466, ZFMK-DIP-00057467, ZFMK-DIP-00057468, ZFMK-DIP-00057469, ZFMK-DIP-00057470.

**Distribution.** Alps, northern Turkey, and Transcaucasia.

**Remarks.**Peck (1988) and Barkalov and Mutin (2018) listed this species from Transcaucasia, and Speight (2018a) included the Caucasus in its distributional range, but it has never been previously reported from Georgia to our knowledge. Reported for Georgia for the first time.

***Syrphus ribesii* (Linnaeus, 1758)**

**Reference.**Radde (1899); Levitin (1962); Tóth (1986); Peck (1988); Gudjabidze (2002) as *Syrphus ribessii* (Linnaeus, 1758) [sic].

**New records.** GEORGIA • 1♂; L1, 15 Jun 2018, S. Bot leg.; • 1♂; L1, 16 Jun 2018, S. Bot leg.; • 1♂; L3, 17 Jun 2018, S. Bot leg.; • 3♂; L4, 18 Jun 2018, S. Bot leg.; • 1♀; L8, 20 Jun 2018, S. Bot leg.; • 3♀; L10, 22 Jun 2018, S. Bot leg.; • 1♀; L11, 29 Jun 2018, S. Bot leg.; • 1♀; L17, 28 Jun 2018, S. Bot leg.; • 1♂ 1♀; L19, 29 Jun 2018, S. Bot leg.; • 1♂; L20, 1 Jul 2018, S. Bot leg.; • *5; L21, 1 Jul 2018, S. Bot obs.; • 1♂; L21, 3 Jul 2018, S. Bot leg.; • 2♂ 4♀; L24, 17 Jul 2018, X. Mengual leg.; ZFMK-DIP-00053829, ZFMK-DIP-00053830, ZFMK-DIP-00054029 = ZFMK-TIS-8000962, ZFMK-DIP-00053831 = ZFMK-TIS-8005590, ZFMK-DIP-00053832, ZFMK-DIP-00054033 = ZFMK-TIS-8000968; • 2♀; L28, 19 Jul 2018, X. Mengual leg.; ZFMK-DIP-00053838, ZFMK-DIP-00054031 = ZFMK-TIS-8000978; • 1♂; L29, 19 Jul 2018, X. Mengual leg.; ZFMK-DIP-00053828; • 1♂; L31, 21 Jul 2018, J. Astrin leg.; ZFMK-TIS-8000144; • 1♀; L31, 17–29 Jul 2018, B. Wipfler leg.; ZFMK-DIP-00054209; • 2♂; L31, 20 Jul 2018, X. Mengual leg.; ZFMK-DIP-00054037, ZFMK-DIP-00054038; • 1♂ 2♀; L33, 22 Jul 2018, X. Mengual leg.; ZFMK-DIP-00053824, ZFMK-DIP-00053837, ZFMK-DIP-00053839 = ZFMK-TIS-8005583; • 3♀; L33, 23 Jul 2018, X. Mengual leg.; ZFMK-DIP-00054030, ZFMK-DIP-00054036, ZFMK-DIP-00054028 = ZFMK-TIS-8000985; • 5♂; L34, 22 Jul 2018, X. Mengual leg.; ZFMK-DIP-00053823, ZFMK-DIP-00053825, ZFMK-DIP-00053826, ZFMK-DIP-00053827 = ZFMK-TIS-8005582, ZFMK-DIP-00054032 = ZFMK-TIS-8000993; • 1♂; L35, 24 Jul 2018, B. Thormann leg.; ZFMK-DIP-00054034 = ZFMK-TIS-8003835; • 1♀; L35, 24 Jul 2018, X. Mengual leg.; ZFMK-DIP-00053836; • 2♀; L36, 24 Jul 2018, X. Mengual leg.; ZFMK-DIP-00053835, ZFMK-DIP-00054035 = ZFMK-TIS-8000999; • 1♂ 2♀; L37, 25 Jul 2018, X. Mengual leg.; ZFMK-DIP-00053822, ZFMK-DIP-00053833, ZFMK-DIP-00053834; • 1♂ 1♀; L57, 3 Aug 2001, J.-H. Stuke leg.; ZFMK-DIP-00057930, ZFMK-DIP-00057920; • 14♂ 20♀; L70, 30 Jun–14 Jul 2018, malaise trap, GGBC-members leg.; ZFMK-TIS-8002694, ZFMK-TIS-8002695, ZFMK-TIS-8002696, ZFMK-DIP-00061318, ZFMK-DIP-00061319, ZFMK-DIP-00061320, ZFMK-DIP-00061321, ZFMK-DIP-00061322, ZFMK-DIP-00061323, ZFMK-DIP-00061324, ZFMK-DIP-00061325, ZFMK-DIP-00061326, ZFMK-DIP-00061327, ZFMK-DIP-00061328, ZFMK-TIS-8002697, ZFMK-TIS-8002698, ZFMK-TIS-8002699, ZFMK-DIP-00061329, ZFMK-DIP-00061330, ZFMK-DIP-00061331, ZFMK-DIP-00061332, ZFMK-DIP-00061333, ZFMK-DIP-00061334, ZFMK-DIP-00061335, ZFMK-DIP-00061336, ZFMK-DIP-00061337, ZFMK-DIP-00061338, ZFMK-DIP-00061339, ZFMK-DIP-00067220, ZFMK-DIP-00067221, ZFMK-DIP-00067222, ZFMK-DIP-00067223, ZFMK-DIP-00067224, ZFMK-DIP-00067225; • 8♂; L70, 30 Jun–14 Jul 2018, malaise trap, GGBC-members leg.; MTD-Dip-A-R-4550; • 15♀; L70, 30 Jun–14 Jul 2018, malaise trap, GGBC-members leg.; MTD-Dip-A-R-4551; • 3♂; L71, 30 Jun–14 Jul 2018, malaise trap, GGBC-members leg.; ZFMK-TIS-8002736, ZFMK-DIP-00067226, ZFMK-DIP-00067227.

**Genetics.** Eleven specimens were sequenced (MN622078, MN622079, MN622080, MN622081, MN622082, MN622083, MN622084, MN622085, MN622086, MN622087, MN622088) and their COI barcodes differ 0–0.46%. The Barcode Index Number Registry lists a BIN for this taxon (BOLD:AAA4570) with an average p-distance variation of 1.29% within the BIN (4.23% max) and a p-distance of 3.02% to the nearest neighbour in BOLD systems, *Syrphus torvus* Osten Sacken, 1875 (BOLD:AAC6088).

**Distribution.** Holarctic.

***Syrphus torvus* Osten Sacken, 1875**

**Reference.**Tóth (1986); Peck (1988); Gudjabidze (2002).

**New records.** GEORGIA • 2♂; L3, 17 Jun 2018, S. Bot leg.; • 1♂; L4, 18 Jun 2018, S. Bot leg.; • 1♀; L5, 18 Jun 2018, S. Bot leg.; • 1♂; L7, 19 Jun 2018, S. Bot leg.; • 1♂ 2♀; L8, 20 Jun 2018, S. Bot leg.; • 1♂; L10, 22 Jun 2018, S. Bot leg.; • 1♀; L19, 29 Jun 2018, S. Bot leg.; • 1♂ 1♀; L28, 19 Jul 2018, X. Mengual leg.; ZFMK-DIP-00053813, ZFMK-DIP-00053819; • 3♂ 1♀; L29, 19 Jul 2018, X. Mengual leg.; ZFMK-DIP-00053814 = ZFMK-TIS-8005589, ZFMK-DIP-00053815, ZFMK-DIP-00053816, ZFMK-DIP-00053821; • 2♂; L31, 23 Jul 2018, A. Reimann leg.; MTD-Dip-A-R-4526; • 2♂; L31, 20 Jul 2018, X. Mengual leg.; ZFMK-DIP-00053817 = ZFMK-TIS-8005581, ZFMK-DIP-00054048; • 1♀; L33, 22 Jul 2018, X. Mengual leg.; ZFMK-DIP-00053820, ZFMK-DIP-00054047 = ZFMK-TIS-8000987; • 1♀; L33, 23 Jul 2018, X. Mengual leg.; ZFMK-DIP-00054047 = ZFMK-TIS-8000987; • 1♂; L34, 22 Jul 2018, X. Mengual leg.; ZFMK-DIP-00053818; • 1♂; L57, 3 Aug 2001, J.-H. Stuke leg.; ZFMK-DIP-00057929; • 1♂ 1♀; L69, 18 Jul 2018, A. Reimann leg.; MTD-Dip-A-R-4501, ZFMK-TIS-8002664; • 10♂ 4♀; L70, 30 Jun–14 Jul 2018, malaise trap, GGBC-members leg.; ZFMK-TIS-8002686, ZFMK-TIS-8002687, ZFMK-TIS-8002688, ZFMK-DIP-00067232, ZFMK-DIP-00067233, ZFMK-DIP-00067234, ZFMK-DIP-00067235, ZFMK-DIP-00067236, ZFMK-DIP-00067237, ZFMK-DIP-00067238, ZFMK-TIS-8002685, ZFMK-DIP-00067228, ZFMK-DIP-00067229, ZFMK-DIP-00067230; • 2♀; L70, 30 Jun–14 Jul 2018, malaise trap, GGBC-members leg.; MTD-Dip-A-R-4577; • 6♂; L70, 30 Jun–14 Jul 2018, malaise trap, GGBC-members leg.; MTD-Dip-A-R-4569.

**Genetics.** Five specimens were successfully sequenced (MN622089, MN622090, MN622091, MN622092, MN622093) and their COI barcodes differ 0–0.46%. The Barcode Index Number Registry lists one BIN for this taxon, BOLD:AAC6088.

**Distribution.** Palaearctic, Western North America, Taiwan, northern India, Nepal, and Thailand.

***Syrphus vitripennis* Megerle in Meigen, 1822**

**Reference.**Levitin (1962) as *Syrphus vitripennis* Mg.; Tóth (1986) as *Syrphus vitripennis* Meigen, 1822; Peck (1988); Gudjabidze (2002).

**New records.** GEORGIA • 2♂ 1♀; L2, 16 Jun 2018, S. Bot leg.; • 1♂ 2♀; L3, 17 Jun 2018, S. Bot leg.; • 1♂; L10, 22 Jun 2018, S. Bot leg.; • 1♀; L12, 24 Jun 2018, S. Bot leg.; • 1♂; L20, 2 Jul 2018, S. Bot leg.; • 1♂ 6♀; L24, 17 Jul 2018, X. Mengual leg.; ZFMK-DIP-00053848 = ZFMK-TIS-8005591, ZFMK-DIP-00053844, ZFMK-DIP-00053845, ZFMK-DIP-00053846, ZFMK-DIP-00053847, ZFMK-DIP-00054040 = ZFMK-TIS-8000965, ZFMK-DIP-00054043 = ZFMK-TIS-8000956; • 1♀; L25, 18 Jul 2018, X. Mengual leg.; ZFMK-DIP-00053843 = ZFMK-TIS-8005585; • 1♀; L31, 21 Jul 2018, J. Astrin leg.; ZFMK-TIS-8000137; • 1♀; L31, 23 Jul 2018, A. Reimann leg.; MTD-Dip-A-R-4514; • 1♂; L34, 22 Jul 2018, X. Mengual leg.; ZFMK-DIP-00054045 = ZFMK-TIS-8000991; • 1♂; L35, 24 Jul 2018, prey of spider *Misumena vatia* Clerck, 1757 [ZFMK Ar20766], H.-J. Krammer leg.; ZFMK-DIP-00054211; • 1♂ 2♀; L37, 25 Jul 2018, X. Mengual leg.; ZFMK-DIP-00054042, ZFMK-DIP-00054041, ZFMK-DIP-00053842; • 1♂; L37, 25 Jul 2018, B. Thormann leg.; ZFMK-DIP-00054046 = ZFMK-TIS-8004101; • 1♂ 2♀; L38, 25 Jul 2018, X. Mengual leg.; ZFMK-DIP-00054039 = ZFMK-TIS-8001002, ZFMK-DIP-00053841, ZFMK-DIP-00054044 = ZFMK-TIS-8001000; • 1♂; L42, 25 Jul 2018, B. Rulik leg.; MTD-Dip-A-R-4549; • 1♂ 1♀; L42, 25 Jul 2018, A. Reimann leg.; MTD-Dip-A-R-4535, ZFMK-TIS-8002676; • 1♂ 1♀; L42, 25 Jul 2018, A. Reimann leg.; MTD-Dip-A-R-4544; • 1♀; L43, 18 Jul 2018, J. Astrin leg.; ZFMK-TIS-8000010; • 1♂; L46, 24 Jul 2001, J.-H. Stuke leg.; ZFMK-DIP-00057926; • 1♀; L49, 4 Aug 2001, J.-H. Stuke leg.; ZFMK-DIP-00057921; • 1♂; L50, 4 Aug 2001, J.-H. Stuke leg.; ZFMK-DIP-00057923; • 1♀; L53, 1 Aug 2001, J.-H. Stuke leg.; ZFMK-DIP-00057927; • 3♂ 1♀; L57, 3 Aug 2001, J.-H. Stuke leg.; ZFMK-DIP-00057922, ZFMK-DIP-00057924, ZFMK-DIP-00057925, ZFMK-DIP-00057928; • 2♂ 2♀; L70, 30 Jun–14 Jul 2018, malaise trap, GGBC-members leg.; ZFMK-TIS-8002689, ZFMK-TIS-8002690, ZFMK-TIS-8002691, ZFMK-TIS-8002692; • 2♂; L70, 30 Jun–14 Jul 2018, malaise trap, GGBC-members leg.; MTD-Dip-A-R-4586; • 2♀; L70, 30 Jun–14 Jul 2018, malaise trap, GGBC-members leg.; MTD-Dip-A-R-4582; • 1♀; L70, 30 Jun–14 Jul 2018, malaise trap, GGBC-members leg.; ZFMK-TIS-8002691; • 1♂; L71, 30 Jun–14 Jul 2018, malaise trap, GGBC-members leg.; ZFMK-TIS-8002735; • 1♀; L72, 29 Jun–13 Jul 2018, malaise trap, GGBC-members leg.; ZFMK-TIS-8002785.

**Genetics.** We sequenced 12 specimens (MN622094, MN622095, MN622096, MN622097, MN622098, MN622099, MN622100, MN622101, MN622102, MN622103, MN622104, MN622105) and their COI barcodes differ 0–0.23%. The Barcode Index Number Registry lists a BIN for this taxon (BOLD:AAB5577) with 0.74% p-distance variation within the BIN (2.57% max), but this BIN also has specimens identified as *Syrphus rectus* Osten Sacken, 1875.

**Distribution.** Palaearctic, Western North America, and Taiwan.

**Remarks.** The specimen ZFMK-TIS-8002691 fits the description of Syrphus rectus subsp. bretoletensis Goeldlin de Tiefenau, 1996 with possession of almost entirely yellow legs and wings with extensive areas bare of microtrichia. The taxonomic status of this subspecies is still in discussion, whether it is a valid subspecies of the North American *Syrphus rectus* Osten Sacken, 1875 or another species (Speight 2018a). Ssymank et al. (1999) tentatively synonymised *S. rectus bretolensis* with *S. vitripennis*, but without explanation. The obtained DNA barcode for ZFMK-TIS-8002691 is identical to the other COI sequences of *S. vitripennis*, and we consider this specimen as *S. vitripennis*.

***Temnostoma bombylans* (Fabricius, 1805)**

**Reference.**Tóth (1986).

**Distribution.** Palaearctic.

***Temnostoma meridionale* Krivosheina & Mamayev, 1962**

**Reference.**Levitin (1962); Peck (1988); Gudjabidze (2002) as *Temnostoma meridionale* Krivocheina et Mamajev [sic]; Krivosheina (2005); Barkalov and Mutin (2018); Speight (2018a).

**Distribution.** Europe, European parts of Russia, and Transcaucasia.

***Temnostoma vespiforme* (Linnaeus, 1758)**

**Reference.**Levitin (1962); Peck (1988); Gudjabidze (2002); Speight (2018a).

**New records.** GEORGIA • 1♂; L3, 17 Jun 2018, S. Bot leg.; • 1♀; L8, 20 Jun 2018, S. Bot leg.

**Distribution.** Needs reassessment; Holarctic.

***Triglyphus primus* Loew, 1840**

**Reference.**Speight (2018a).

**Distribution.** Europe, Russia, Transcaucasia, and Korea.

***Tropidia scita* (Harris, 1778)**

**Reference.**Peck (1988); Speight (2018a).

**Distribution.** Palaearctic, but not in northern Africa.

**Remarks.** The year of publication for this species is a convention. Peck (1988) used the conventional dates based on Lisney (1960): 1776 for decad 1, 1776? for decad 2, and 1780? for decads 3, 4, and 5. Evenhuis (1997: page 342) found that the decad 2, where *Musca scitus* is described on page 41, was dated as 1778 in the “*Discours préliminaires*” to the *Encyclopédie méthodique par ordre des matières* – *Insectes*. Thus, the year of publication should be 1778.

***Volucella bombylans* (Linnaeus 1758)**

**Reference.**Portschinsky (1877); Radde (1899) as Volucella bombylans var. plumata (De Geer, 1776) and as Volucella bombylans var. caucasica Portschinsky, 1877; Peck (1988); Gudjabidze (2002) as *Volucella bombylans* Zetterstendt, 1843 [sic]; Speight (2018a).

**New records.** GEORGIA • 1♂ 1♀ *1 var. plumata; L3, 17 Jun 2018, S. Bot leg. & obs.; • 1♂ (abdomen almost completely white haired); L4, 18 Jun 2018, S. Bot leg.; • 1♀ var. plumata; L4, 18 Jun 2018, S. Bot leg.; • 1♂ *30 var. plumata; L6, 19 Jun 2018, S. Bot leg.; • 1♂ 1♀ var. plumata; L10, 22 Jun 2018, S. Bot leg.; • 1♀ var. plumata; L15, 26 Jun 2018, S. Bot leg.; • 1♂ 1♀ var. haemorrhoidalis; L16, 27 Jun 2018, S. Bot leg.; • 1♂ *20 var. plumata; L19, 29 Jun 2018, S. Bot leg.; • *10 var. plumata; L21, 1 Jul 2018, S. Bot obs.; • 1♂; L28, 19 Jul 2018, X. Mengual leg.; ZFMK-DIP-00053656 = ZFMK-TIS-8005539; • 3♂ 5♀; L33, 22 Jul 2018, X. Mengual leg.; ZFMK-DIP-00053657, ZFMK-DIP-00053660, ZFMK-DIP-00053971 = ZFMK-TIS-8003426, ZFMK-DIP-00053658, ZFMK-DIP-00053659, ZFMK-DIP-00053664 = ZFMK-TIS-8005547, ZFMK-DIP-00053972 = ZFMK-TIS-8003427, ZFMK-DIP-00053973 = ZFMK-TIS-8003435; • 3♀; L35, 24 Jul 2018, X. Mengual leg.; ZFMK-DIP-00053661, ZFMK-DIP-00053662, ZFMK-DIP-00053970 = ZFMK-TIS-8003450; • 1♂; L36, 24 Jul 2018, X. Mengual leg.; ZFMK-DIP-00053663; • 1♂; L57, 2 Aug 2001, J.-H. Stuke leg.; ZFMK-DIP-00058270; • 1♂; L57, 3 Aug 2001, J.-H. Stuke leg.; ZFMK-DIP-00058269; • 1♀; L71, 30 Jun–14 Jul 2018, malaise trap, GGBC-members leg.; ZFMK-TIS-8002727.

**Genetics.** Three specimens were sequenced (MN622106, MN622107, MN622108) with identical COI barcode. The Barcode Index Number Registry lists a BIN for this taxon (BOLD:AAB8627) with 0.96% p-distance variation within the BIN members (3.1% max) and 5.17% p-distance to the nearest neighbour, *Volucella inanis* (Linnaeus 1758) (BOLD:AAZ4733). The BIN for *V. bombylans* also has other species, indicating an overlap between intra- and interspecific distances.

**Distribution.** Holarctic.

***Volucella inanis* (Linnaeus 1758)**

**Reference.**Levitin (1962); Peck (1988); Gudjabidze (2002).

**New records.** GEORGIA • 1♂; L29, 19 Jul 2018, X. Mengual leg.; ZFMK-DIP-00053654 = ZFMK-TIS-8005538; • 1♂; L37, 25 Jul 2018, X. Mengual leg.; ZFMK-DIP-00053655 = ZFMK-TIS-8005546; • 1♂; L49, 4 Aug 2001, J.-H. Stuke leg.; ZFMK-DIP-00058082.

**Genetics.** We sequenced two specimens (MN622109, MN622110) and their COI barcode were identical. The Barcode Index Number Registry lists a BIN for this taxon (BOLD:AAZ4733) with an average p-distance variation of 0.04% within the BIN members (0.31% max).

**Distribution.** Palaearctic.

***Volucella inflata* (Fabricius, 1794)**

**Reference.**Tóth (1986); Peck (1988); Gudjabidze (2002) as *Volucella inflata* (Fallen, 1817) [sic]; Speight (2018a).

**Distribution.** Europe, European parts of Russia, and Transcaucasia.

***Volucella pellucens* (Linnaeus, 1758)**

**Reference.**Levitin (1962); Peck (1988); Gudjabidze (2002); Speight (2018a).

**New records.** GEORGIA • *1♂; L1, 16 Jun 2018, S. Bot obs.; • *1♂; L8, 20 Jun 2018, S. Bot obs.; • 1♂ *1♂; L16, 27 Jun 2018, S. Bot leg. & obs.; • 1♂; L20, 30 Jun 2018, S. Bot leg.; • 1♂; L28, 19 Jul 2018, X. Mengual leg.; ZFMK-DIP-00053667; • 1♂ 1♀; L35, 24 Jul 2018, X. Mengual leg.; ZFMK-DIP-00053668 = ZFMK-TIS-8005541, ZFMK-DIP-00053974 = ZFMK-TIS-8003451; • 3♀; L36, 24 Jul 2018, X. Mengual leg.; ZFMK-DIP-00053669, ZFMK-DIP-00053670, ZFMK-DIP-00053671; • 1♀; L37, 25 Jul 2018, B. Thormann leg.; ZFMK-DIP-00054052 = ZFMK-TIS-8004115; • 1♀; L37, 25 Jul 2018, J. Thormann leg.; ZFMK-DIP-00054050 = ZFMK-TIS-8004077; • 2♀; L38, 25 Jul 2018, X. Mengual leg.; ZFMK-DIP-00053666 = ZFMK-TIS-8005548, ZFMK-DIP-00054051 = ZFMK-TIS-8001003; • 1♀; L42, 25 Jul 2018, A. Reimann leg.; MTD-Dip-A-R-4532.

**Genetics.** A single specimen was successfully sequenced (MN622111). The Barcode Index Number Registry lists a BIN for this taxon (BOLD:AAH7775) with an average p-distance of 0.04% within the BIN (0.43% max) and a p-distance of 2.31% to the nearest neighbour, *Volucella zonaria* (Poda, 1761) (BOLD:AAH7785).

**Distribution.** Palaearctic and Indomalayan Region.

***Volucella zonaria* (Poda, 1761)**

**Reference.**Radde (1899); Levitin (1962); Peck (1988); Gudjabidze (2002).

**New records.** GEORGIA • 1♂; L21, 2 Jul 2018, S. Bot leg.; • *1♂; L22, 3 Jul 2018, S. Bot obs.; • 1♂; L24, 17 Jul 2018, X. Mengual leg.; ZFMK-DIP-00053665 = ZFMK-TIS-8005540; • 1♂ 1♀; L38, 25 Jul 2018, B. Thormann leg.; ZFMK-DIP-00054053 = ZFMK-TIS-8004232, ZFMK-DIP-00054054 = ZFMK-TIS-8004231; • 1♀; L51, 24 Jul 2001, J.-H. Stuke leg.; ZFMK-DIP-00058080; • 1♀; L64, 23 Jul 2001, J.-H. Stuke leg.; ZFMK-DIP-00058081; • 1♂; L42, 25 Jul 2018, A. Reimann leg.; MTD-Dip-A-R-4533.

**Genetics.** The two specimens sequenced (MN622112, MN622113) have identical COI barcode. The Barcode Index Number Registry lists a BIN for this taxon (BOLD:AAH7785) with no variation within the BIN members and a p-distance of 2.31% with the nearest neighbour, *Volucella pellucens* (BOLD:AAH7775).

**Distribution.** Palaearctic, but not in Northern Europe.

**Xanthandrus (Xanthandrus) comtus (Harris, 1778)**

**Reference.**Levitin (1962) as *Xanthandrus comptus* Harr. [sic]; Tóth (1986); Peck (1988); Gudjabidze (2002) as *Xanthandrus compus* Harris, 1776 [sic]; Speight (2018a).

**New records.** GEORGIA • 1♂; L10, 22 Jun 2018, S. Bot leg.; • 1♀; L11, 29 Jun 2018, S. Bot leg.; • 2♀; L15, 26 Jun 2018, S. Bot leg.; • 1♀; L16, 27 Jun 2018, S. Bot leg.; • 1♂; L19, 29 Jun 2018, S. Bot leg.; • 1♂ *4; L20, 1 Jul 2018, S. Bot leg.; • 1♀; L20, 2 Jul 2018, S. Bot leg.; • 6♂; L24, 17 Jul 2018, X. Mengual leg.; ZFMK-DIP-00053677, ZFMK-DIP-00053678, ZFMK-DIP-00053679, ZFMK-DIP-00053680, ZFMK-DIP-00053681, ZFMK-DIP-00054086 = ZFMK-TIS-8000963; • 1♂ 1♀; L25, 18 Jul 2018, X. Mengual leg.; ZFMK-DIP-00053683, ZFMK-DIP-00053682 = ZFMK-TIS-8005550; • 2♀; L31, 23 Jul 2018, A. Reimann leg.; MTD-Dip-A-R-4525; • 2♂ 1♀; L31, 23 Jul 2018, A. Reimann leg.; MTD-Dip-A-R-4512; • 1♀; L31, 23 Jul 2018, A. Reimann leg.; ZFMK-TIS-8002672; • 1♂; L31, 21 Jul 2018, J. Astrin leg.; ZFMK-TIS-8000126; • 1♀; L35, 24 Jul 2018, X. Mengual leg.; ZFMK-DIP-00054088; • 2♂ 2♀; L36, 24 Jul 2018, X. Mengual leg.; ZFMK-DIP-00053675, ZFMK-DIP-00053676 = ZFMK-TIS-8005558, ZFMK-DIP-00053674, ZFMK-DIP-00054085 = ZFMK-TIS-8000995; • 1♀; L40, 18 Jul 2018, B. Thormann leg.; ZFMK-DIP-00054087 = ZFMK-TIS-8002957; • 2♂ 2♀; L70, 30 Jun–14 Jul 2018, malaise trap, GGBC-members leg.; MTD-Dip-A-R-4585, ZFMK-TIS-8002701, MTD-Dip-A-R-4581, ZFMK-TIS-8002700; • 1♂ 1♀; L71, 30 Jun–14 Jul 2018, malaise trap, GGBC-members leg.; MTD-Dip-A-R-4566, ZFMK-TIS-8002737; • 2♀; L72, 29 Jun–13 Jul 2018, malaise trap, GGBC-members leg.; MTD-Dip-A-R-4575, ZFMK-TIS-8002771.

**Genetics.** We sequenced seven specimens (MN622114, MN622115, MN622116, MN622117, MN622118, MN622119, MN622120) and their COI barcodes differ 0–0.46%. Our COI barcodes are very similar to previously published COI sequnces of the same species (99.54–100% similarity), but they are also 100% similar to a private sequence of *Xanthandrus babyssa* (Walker, 1849) from Madeira, Portugal.

**Distribution.** Palaearctic.

**Remarks.** The year of publication for this species is a convention. Peck (1988) used the conventional dates based on Lisney (1960): 1776 for decad 1, 1776? for decad 2, and 1780? for decads 3, 4, and 5. Evenhuis (1997: page 342) found that the decad 2, where *Musca comtus* is described on page 47, was dated as 1778 in the “*Discours préliminaires*” to the *Encyclopédie méthodique par ordre des matières* – *Insectes*. Thus, the year of publication should be 1778.

***Xanthogramma citrofasciatum* (De Geer, 1776)**

**Reference.**Tóth (1986); Peck (1988); Gudjabidze (2002); Speight (2018a).

**Distribution.** Europe, Transcaucasia, European parts of Russia into Siberia.

**Remarks.**Thompson et al. (1982) synonymised *Musca citrofasciata* De Geer, 1776 under *Musca festiva* Linnaeus, 1758 (= *Xanthogramma festiva*) and explained that the name *festiva* was wrongly applied to a species of the genus *Chrysotoxum* Meigen, 1803 by several authors. Iliff and Chandler (2000) proposed to keep the usage of Chrysotoxum festivum (Linnaeus, 1758) and Xanthogramma citrofasciatum (De Geer, 1776) and designated neotypes. The International Commission on Zoological Nomenclature (ICZN) voted to preserve the neotypes and names’ usage as proposed by Iliff and Chandler (2000) (ICZN 2001).

***Xanthogramma dives* (Rondani, 1857)**

**New records.** GEORGIA • 1♀; L71, 30 Jun–14 Jul 2018, malaise trap, GGBC-members leg.; ZFMK-TIS-8002731; • 1♀; L72, 29 Jun–13 Jul 2018, malaise trap, GGBC-members leg.; ZFMK-TIS-8002766.

**Genetics.** The two collected specimens were sequenced (MN622121, MN622122) and their COI barcodes are identical. Our COI sequences are identical (100% similarity) with published sequences of *Xanthogramma pedissequum* (Harris, 1778) and *X. stackelbergi* Violovich, 1975.

**Distribution.** Europe, but it needs reassessment due to confusion with *X. stackelbergi* and *X. pedissequum* (Speight 2018a).

**Remarks.** As shown by Nedeljković et al. (2018: fig. 40), *X. dives*, *X. stackelbergi* and *X. pedissequum* share some COI haplotypes and the separation of these taxa using barcoding is not straightforward. Reported for Georgia for the first time.

***Xanthogramma maculipenne* Mik, 1887**

**Reference.**Peck (1988).

**Distribution.** Former Yugoslavia, Iran and Transcaucasia.

**Remarks.**Speight (2018a) did not list this taxon as a European species as it has not been mentioned in any recent literature from the former Yugoslavia. Mik (1887) described this species from an undefined number of males and females collected in Göygöl (= Helenendorf = Khanlar), Azerbaijan. He stated that his new species was similar to *X. pedissequum* (as *ornatum* Meigen) but also mentioned their differences: four yellow maculae on the pleuron (like in *X. dives* and *X. stackelbergi*; see Van Steenis et al. 2014), almost entirely dark metatibia, wing with two dark maculae (one at the apical part of the cell r_2+3_ and another one reaching cells r_1_ and r_4+5_), and the coloration of the membrane between abdominal tergites and sternites (yellow between the tergite 2 and sternite 2, and also yellow on basal half between tergite 3 and sternite 3). Violovitsh (1975) keyed out *X. maculipenne* near *Xanthogramma evanescens* Becker in Becker & Stein, 1913, a taxon described from Morocco, based on a dark apical macula on the wing; but *X. dives* was not included in Violovitsh (1975) as it was considered a junior synonym of X. *pedissequum* at that time. Based on the original description, this taxon is very similar to *X. dives*. The study of the type material is needed to resolve and confirm the identity of this taxon.

***Xanthogramma pedissequum* (Harris, 1778)**

**Reference.**Levitin (1962) as *Xanthogramma ornatum* Mg.; Tóth (1986); Peck (1988); Gudjabidze (2002) as *Xanthogramma pedisequm* Harris, 1776 [sic].

**Distribution.** Europe, but it needs reassessment due to confusion with *X. stackelbergi* and *X. dives*.

**Remarks.***Xanthogramma ornatum* (Meigen, 1822) was listed as a synonym by Peck (1988).

The year of publication for this species was a convention. Peck (1988) used the conventional dates as established by Lisney (1960): 1776 for decad 1, 1776? for decad 2, and 1780? for decads 3, 4, and 5. Evenhuis (1997: page 342) found that the decad 2, where *Musca pedissequus* is described on page 61, was dated as 1778 in the “*Discours préliminaires*” to the *Encyclopédie méthodique par ordre des matières* – *Insectes*. Thus, the year of publication should be 1778.

***Xanthogramma stackelbergi* Violovich, 1975**

**New records.** GEORGIA • 1♀; L35, 24 Jul 2018, X. Mengual leg.; ZFMK-DIP-00054098 = ZFMK-TIS-8000953.

**Genetics.** We sequenced the single collected female (MN622123). See comments on Genetics and Remarks under *X. dives*.

**Distribution.** Needs reassessment, but known from Europe, Crimea, and European parts of Russia.

**Remarks.** Reported for Georgia for the first time.

**Xylota (Xylota) abiens Wiedemann in Meigen, 1822**

**Reference.**Peck (1988); Speight (2018a).

**Distribution.** Palaearctic.

**Xylota (Xylota) florum (Fabricius, 1805)**

**Reference.**Peck (1988); Speight (2018a).

**Distribution.** Northern and Central Europe, Transcaucasia, eastwards into Siberia.

**Xylota (Xylota) ignava (Panzer, 1798)**

**Reference.**Peck (1988); Barkalov and Mutin (2018).

**New records.** GEORGIA • 1♂ 1♀ *1; L10, 22 Jun 2018, S. Bot leg. & obs.

**Distribution.** Palaearctic, but not in northern Africa.

**Xylota (Xylota) segnis (Linnaeus, 1758)**

**Reference.**Levitin (1962) as *Zelima segnis* L.; Tóth (1986); Peck (1988); Gudjabidze (2002); Barkalov and Mutin (2018); Speight (2018a).

**New records.** GEORGIA • 1♂ *1; L21, 2 Jul 2018, S. Bot leg. & obs.; • 6♂ 2♀; L24, 17 Jul 2018, X. Mengual leg.; ZFMK-DIP-00053761, ZFMK-DIP-00053762, ZFMK-DIP-00053763, ZFMK-DIP-00053764 = ZFMK-TIS-8005567, ZFMK-DIP-00053765, ZFMK-DIP-00054105 = ZFMK-TIS-8000961, ZFMK-DIP-00053767, ZFMK-DIP-00054104 = ZFMK-TIS-8000958; • 1♂; L25, 18 Jul 2018, X. Mengual leg.; ZFMK-DIP-00054107; • 2♂; L31, 23 Jul 2018, A. Reimann leg.; MTD-Dip-A-R-4520; • 2♀; L37, 25 Jul 2018, X. Mengual leg.; ZFMK-DIP-00053766 = ZFMK-TIS-8005575, ZFMK-DIP-00054106; • 2♀; L69, 23 Jul 2018, A. Reimann leg.; MTD-Dip-A-R-4518, ZFMK-TIS-8002673.

**Genetics.** Three specimens were sequenced (MN622124, MN622125, MN622126) and their COI barcodes are identical. The Barcode Index Number Registry lists a BIN for this taxon (BOLD:AAG4673) with an average p-distance of 0.03% among the BIN members (0.49% max) and a p-distance of 5.26% to the nearest neighbour in BOLD systems, *Xylota coquilletti* Hervé-Bazin, 1914 (BOLD:AAZ0875).

**Distribution.** Palaearctic and eastern North America.

**Xylota (Xylota) sylvarum (Linnaeus, 1758)**

**Reference.**Levitin (1962) as *Zelima silvarum* L. [sic]; Tóth (1986); Peck (1988) as *Xylota silvarum* Linnaeus, 1758 [sic]; Gudjabidze (2002).

**New records.** GEORGIA • 3♂ 1♀; L24, 17 Jul 2018, X. Mengual leg.; ZFMK-DIP-00053751, ZFMK-DIP-00053752, ZFMK-DIP-00053979 = ZFMK-TIS-8003455, ZFMK-DIP-00053760; • 2♀; L31, 23 Jul 2018, A. Reimann leg.; MTD-Dip-A-R-4511, ZFMK-TIS-8002671; • 1♀; L31, 21 Jul 2018, D. Tarkhnishvili leg.; ZFMK-TIS-8000092; • 1♂; L37, 25 Jul 2018, X. Mengual leg.; ZFMK-DIP-00053756; • 1♀; L38, 25 Jul 2018, X. Mengual leg.; ZFMK-DIP-00053757; • 1♂ 1♀; L69, 18 Jul 2018, A. Reimann leg.; MTD-Dip-A-R-4519, MTD-Dip-A-R-4500.

**Genetics.** Two specimens were sequenced (MN622127, MN622128) and their COI barcodes are identical. The Barcode Index Number Registry lists a BIN for this taxon (BOLD:AAZ8002) with an average p-distance of 0.15% within the BIN (1.37% max).

**Remarks.** See under *X. xanthocnema* Collin, 1939.

**Distribution.** Palaearctic, but not in northern Africa.

**Xylota (Xylota) tarda Meigen, 1822**

**Reference.**Levitin (1962) as *Zelima tarda* Mg.; Peck (1988); Gudjabidze (2002); Barkalov and Mutin (2018); Speight (2018a).

**New records.** GEORGIA • 4♂; L3, 17 Jun 2018, S. Bot leg.; • 1♂; L8, 20 Jun 2018, S. Bot leg.

**Distribution.** Palaearctic, but not in northern Africa.

**Remarks.** The new specimens reported here are remarkably larger in size compared to Western European specimens.

**Xylota (Xylota) xanthocnema Collin, 1939**

**Reference.**Peck (1988); Speight (2018a).

**New records.** GEORGIA • 1♂ 2♀; L24, 17 Jul 2018, X. Mengual leg.; ZFMK-DIP-00053753, ZFMK-DIP-00053758, ZFMK-DIP-00054018 = ZFMK-TIS-8000957; • 1♂ 1♀; L25, 18 Jul 2018, X. Mengual leg.; ZFMK-DIP-00053754 = ZFMK-TIS-8005566, ZFMK-DIP-00053978 = ZFMK-TIS-8003447; • 1♀; L30, 19 Jul 2018, X. Mengual leg.; ZFMK-DIP-00053759 = ZFMK-TIS-8005574; • 1♂; L31, 23 Jul 2018, A. Reimann leg.; MTD-Dip-A-R-4522; • 1♂; L36, 24 Jul 2018, X. Mengual leg.; ZFMK-DIP-00053755; • 1♀; L38, 25 Jul 2018, J. Thormann leg.; ZFMK-DIP-00054103 = ZFMK-TIS-8004026; • 2♀; L71, 30 Jun–14 Jul 2018, malaise trap, GGBC-members leg.; MTD-Dip-A-R-4565, ZFMK-TIS-8002755.

**Genetics.** Four specimens with identical COI barcode were sequenced (MN622129, MN622130, MN622131, MN622132). Our barcodes are very similar (98.32–100% similarity) to other sequences of *X. xanthocnema*, and quite similar to sequences of other species, such as *X. florum* (97.05% similarity) or *X. sylvarum* (96.56% similarity).

**Distribution.** Europe, European parts of Russia, and Transcaucasia.

**Remarks.** The specimens reported here do have partially black metatibiae. Using the comprehensive identification key to *Xylota* species by Speight (2017) they would key out as *Xylota sylvarum*, but the male genitalia clearly confirm their identity as *X. xanthocnema*. In overall appearance these specimens are smaller than those of *X. sylvarum* and the golden abdominal hairs are lighter and restricted to a smaller area. All the material previously identified as *X. sylvarum* from this region needs to be re-evaluated.

### Unrecognized taxa

Gudjabidze (2002) has many systematic and nomenclatural errors. We have tried to correct them updating the nomenclature and assuming some freedom regarding the authorship stated by Gudjabidze. There are three species names that we could not place in the current systematics of Syrphidae and thus, we left them as *nomen dubium*. These taxa are cited by Gudjabidze (2002) as *Syrphus campestris* Verrall, *Chrysotoxum macqarti* Loew, 1848 and *Arctophila musatovi* (Fallen, 1817) [sic]. For the first name we have no suggestion; we believe that the second name refers to *Chrysogaster macquarti* Loew, 1843 (see next paragraph); and the third name might refer to *Syrphus mussitans* Fabricius, 1777, a junior synonym of *Sericomyia superbiens* (Müller, 1776).

Peck (1988) cited *Chrysogaster macquarti* Loew, 1843 from Transcaucasia and Gudjabidze (2002) cited it as *Chrysogaster macquart* Loew, 1848 [sic] and as *Chrysotoxum macqarti* Loew, 1848 [sic]. According to Maibach et al. (1994b)*Chrysogaster macquarti* Loew, 1843 is a composite taxon in which *Melanogaster aerosa* (Loew, 1843) and *Melanogaster parumplicata* (Loew, 1840) were confused. Thus, we decided to exclude from the present work the citations of *C. macquarti* by Peck (1988) and Gudjabidze (2002), as it is not possible to corroborate the identity of this material and it could either be *M. aerosa* or *M. parumplicata* or even both.

Speight (2018a) stated that *Syritta vittata* Portschinsky, 1875 reaches the south-east edge of Europe, in the Caucasus. Lyneborg and Barkemeyer (2005) gave the following distribution range: from South Russia over the Central Asiatic republics to Iran and westernmost Pakistan. Lyneborg and Barkemeyer (2005) studied two specimens (male and female) from “Asia Centr.”, Chiva. This location may refer to Khiva, Uzbekistan, and the locality in South Russia studied by Lyneborg and Barkemeyer (2005) should be Sarepta (now Krasnoarmeysky Rayon, a district of Volgograd). Volvograd Oblast is part of the Northern Caucasus, but the northern part of the Northern Caucasus is part of the typically called European parts of Russia and is far from the Caucasus Mountain range (Greater Caucasus). Thus, we decided to not include this species in the present checklist, but we acknowledge the possibility that this species can occur in Georgia.

## Discussion

The flower fly fauna of Georgia is quite similar to the fauna found in Central Europe, with some species endemic of Transcaucasia and a few more species occurring also in Turkey and Iran (see Kustov 2006). The DNA COI sequences obtained for the present work are the first DNA barcodes of Syrphidae ever published from Georgia and enlarge the knowledge on the molecular variability for the studied species.

Five very common Palaearctic species were collected abundantly, i.e., *Melanostoma mellinum* (218 specimens), *Syrphus ribesii* (109), *Sphaerophoria scripta* (94), *Eristalis tenax* (88), and *Melanostoma scalare* (86). Two species recorded for the first time from Georgia were collected in relatively large numbers, i.e., *Neoascia subannexa* (36) and *Syrphocheilosia claviventris* (25). These taxa are not ubiquitous but can be found in relatively large numbers locally on sites they occur. This prompts us to continue our survey of the Syrphidae fauna in Georgia and in the Caucasus Region in the coming years, as we expect more species to be recorded from this country.

## Supplementary materials

Supplementary material 1 Neighbor-joining tree using Jukes-Cantor model of the 328 COI sequences of the Syrphidae from Georgia, sequenced in our study Data type: molecular data https://binary.pensoft.net/file/385817This dataset is made available under the Open Database License (http://opendatacommons.org/licenses/odbl/1.0/). The Open Database License (ODbL) is a license agreement intended to allow users to freely share, modify, and use this Dataset while maintaining this same freedom for others, provided that the original source and author(s) are credited.Ximo Mengual, Sander Bot, Tinatin Chkhartishvili, André Reimann, Jana Thormann, Laura von der Mark
