# Supplementary material for: Checklist of hover flies (Diptera, Syrphidae) of the Republic of Georgia
Source: Zookeys. 2020 Mar 2;916:1–123. doi: 10.3897/zookeys.916.47824 (PMC7062849; doi:10.3897/zookeys.916.47824)
Supplement: Supplementary material 1 [file zookeys-916-001-s001.pdf]

Supplementary material

Checklist of hoverflies (Diptera: Syrphidae) of Georgia

Ximo Mengual[1], Sander Bot[2], Tinatin Chkhartishvili[3], André Reimann[4], Jana Thormann[1], Laura von der Mark[1]

[1] Zoologisches Forschungsmuseum Alexander Koenig, Leibniz-Institut für Biodiversität der Tiere, Adenauerallee 160, D-53113 Bonn, Germany. Email: X.Mengual@leibniz-zfmk.de; J.Thormann@leibniz-zfmk.de; L.vonderMark@leibniz-zfmk.de

[2] Kerklaan 30E, 9751 NN Haren, the Netherlands. E-mail: botsander@gmail.com

[3] Insititute of Zoology, Ilia State University, Chavchavadze Avenue 32, 0179, Tbilisi, Georgia. Email: tinatin.chkhartishvili.1@iliauni.edu.ge

[4] Senckenberg Naturhistorische Sammlungen Dresden, Museum für Tierkunde, Königsbrücker Landstraße 159, D-01109, Dresden,Germany. Email: andre.reimann@senckenberg.de

Supplementary file 1: Figure 1. Neighbor-joining tree using Jukes-Cantor model of the 328 COI sequences of the Syrphidae from Georgia, sequenced in our study.

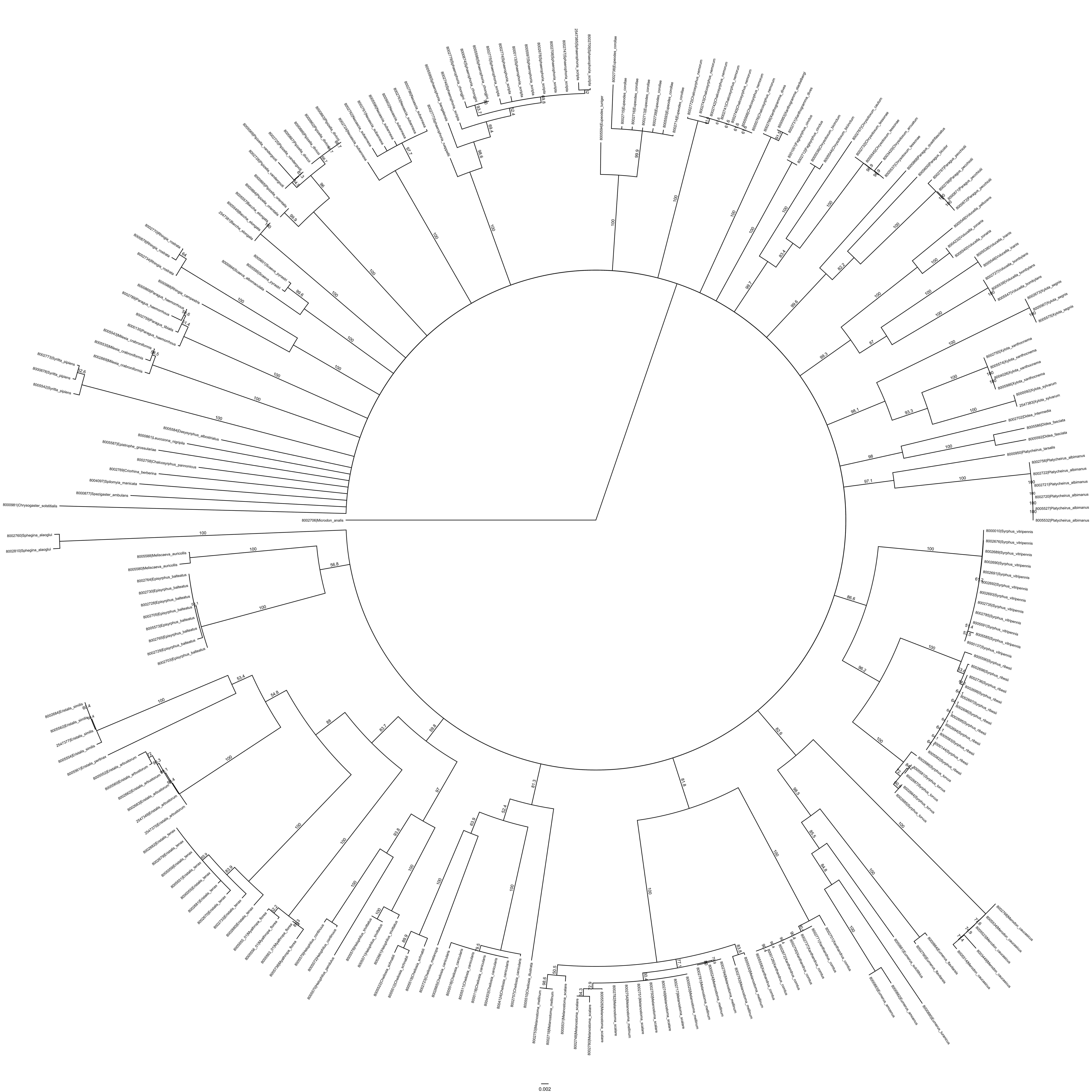
8002706|Microdon_analis
8000981|Chrysogaster_solstitialis
8000877|Spazigaster_ambulans
8004097|Spilomyia_manicata
8002769|Criorhina_berberina
8002758|Chalcosyrphus_pannonicus
8005587|Epistrophe_grossulariae
8000861|Leucozona_nigripila
8005584|Dasysyrphus_albostriatus
8005542|Syritta_pipiens
8000878|Syritta_pipiens
8002773|Syritta_pipiens
8002760|Sphegina_alaoglui
8002810|Sphegina_alaoglui
8005588|Meliscaeva_auricollis
8005580|Meliscaeva_auricollis
8002764|Episyrphus_balteatus
8002730|Episyrphus_balteatus
8002728|Episyrphus_balteatus
8002705|Episyrphus_balteatus
8005573|Episyrphus_balteatus
8002765|Episyrphus_balteatus
8002729|Episyrphus_balteatus
8002703|Episyrphus_balteatus
8002702|Didea_intermedia
8005586|Didea_fasciata
8005592|Didea_fasciata
8000950|Platycheirus_tarsalis
8002756|Platycheirus_albimanus
8002722|Platycheirus_albimanus
8002721|Platycheirus_albimanus
8002720|Platycheirus_albimanus
8005527|Platycheirus_albimanus
8005532|Platycheirus_albimanus
8000010|Syrphus_vitripennis
8002676|Syrphus_vitripennis
8002689|Syrphus_vitripennis
8002690|Syrphus_vitripennis
0.002
